# Polymer Photoelectrodes for Solar Fuel Production: Progress and Challenges

**DOI:** 10.1021/acs.chemrev.1c00971

**Published:** 2022-06-14

**Authors:** Madasamy Thangamuthu, Qiushi Ruan, Peter Osei Ohemeng, Bing Luo, Dengwei Jing, Robert Godin, Junwang Tang

**Affiliations:** † Department of Chemical Engineering, University College London, Torrington Place, London WC1E 7JE, U.K.; ‡ School of Materials Science and Engineering, Southeast University, Nanjing 211189, China; § Department of Chemistry, The University of British Columbia, Okanagan Campus, 3247 University Way, Kelowna, BC V1V 1V7, Canada; ∥ School of Chemical Engineering and Technology, Xi’an Jiaotong University, Xi’an 710049, China; ⊥ International Research Center for Renewable Energy & State Key Laboratory of Multiphase Flow in Power Engineering, Xi’an Jiaotong University, Xi’an 710049, China

## Abstract

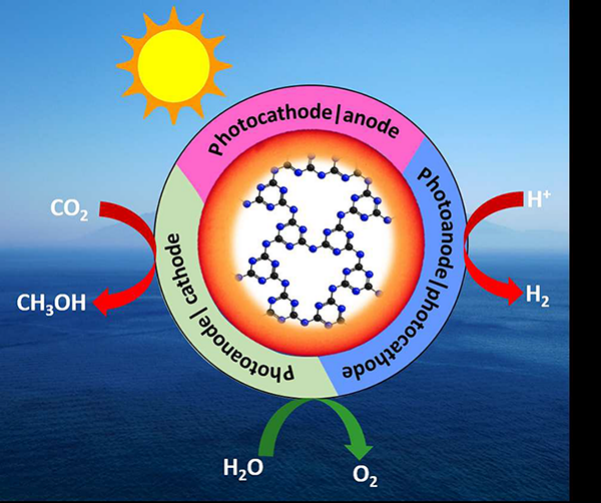

Converting solar energy to fuels has attracted substantial interest over the past decades because it has the potential to sustainably meet the increasing global energy demand. However, achieving this potential requires significant technological advances. Polymer photoelectrodes are composed of earth-abundant elements, e.g. carbon, nitrogen, oxygen, hydrogen, which promise to be more economically sustainable than their inorganic counterparts. Furthermore, the electronic structure of polymer photoelectrodes can be more easily tuned to fit the solar spectrum than inorganic counterparts, promising a feasible practical application. As a fast-moving area, in particular, over the past ten years, we have witnessed an explosion of reports on polymer materials, including photoelectrodes, cocatalysts, device architectures, and fundamental understanding experimentally and theoretically, all of which have been detailed in this review. Furthermore, the prospects of this field are discussed to highlight the future development of polymer photoelectrodes.

## 1 Need for solar fuels

The energy required for the planet is primarily derived from the sun through natural photosynthesis.^5^ Over time, energy demand has substantially increased due to the industrial revolution, the increasing population, and the increase in quality of life-related human activities such as transportation, electricity generation, and heating.^6^ At present, fossil fuels such as oil, coal, and natural gas together satisfy ∼80% of the global energy demand largely driven by characteristics such as high energy density, low cost, availability, ease of handling, storage, and transportation.^7^ Nevertheless, the combustion of fossil fuels releases a massive amount of CO_2_, a key greenhouse gas, into the atmosphere leading to global warming and climate change.^8^ On the other hand, worldwide energy consumption is projected to double by 2050 due to the rapid upsurge of the population.^9^ To address these issues, the development of sustainable energy technologies is essential.

Fascinatingly, the sun provides 1000 times higher energy (1.9 × 10^8^ TWh/yr) than the global energy consumption (1.3 × 10^5^ TWh/yr). To harness this primary energy, remarkable advances have been made to produce sustainable electricity by solar cells.^10^ Although solar electricity will surely play a key role in future energy infrastructure, there is a significant need to produce high energy density fuels and store them for prolonged time use.^11^ Thus, conversion of solar energy into chemical fuels, especially via hydrogen (H_2_) generation from earth-abundant water or high value-added chemical synthesis involving utilization of CO_2_, is highly desirable. Storing solar energy in the form of chemical bonds, similar to natural photosynthesis, is very attractive as it could be released upon demand. For instance, solar-derived green H_2_ has the potential to replace gray or blue H_2_, which has been vastly used for ammonia synthesis and in refineries.^12^ It is worth noting that the H_2_ specific mass energy (140 MJ kg^–1^) is much higher than that of natural gas (55 MJ kg^–1^), gasoline (34.2 MJ kg^–1^), diesel (45 MJ kg^–1^), and coal (24 MJ kg^–1^).^13^ Presently, the H_2_ fuel cell has been demonstrated to power aircraft, rail vehicles, buses, and passenger cars.^14^ Similarly, high-value chemicals can be produced from CO_2_ including liquid fuels, e.g., methanol and ethanol, which can be directly used in place of fossil fuels. More importantly, alcohols are easy to store in liquid form for an extended time and can be used right away with the existing energy distribution infrastructure. Therefore, this review mainly covers water splitting and CO_2_ reduction reactions using polymer photoelectrodes driven by solar energy.

### 1.1 Solar Fuel Production Techniques

To substitute both gray and blue H_2_ production technologies, electrocatalysis was proposed decades ago, which is purely driven by an external bias, using the appropriate electrocatalyst-loaded anode and cathode for separate H_2_ and O_2_ production.^15,16^ The faradaic efficiency (FE) and product selectivity depend on the electrocatalyst, electrolyte, and applied potential. Electrocatalytic water splitting and CO_2_ reduction have been reviewed extensively.^17−20^ Although this field has progressed, conventional electrolysis has high capital and operational cost. Some other methods such as thermolysis, photolysis, and biomass conversion techniques for green H_2_ production are also under active development, and the reader can find more details in these reviews.^21−24^

**Figure 1:**
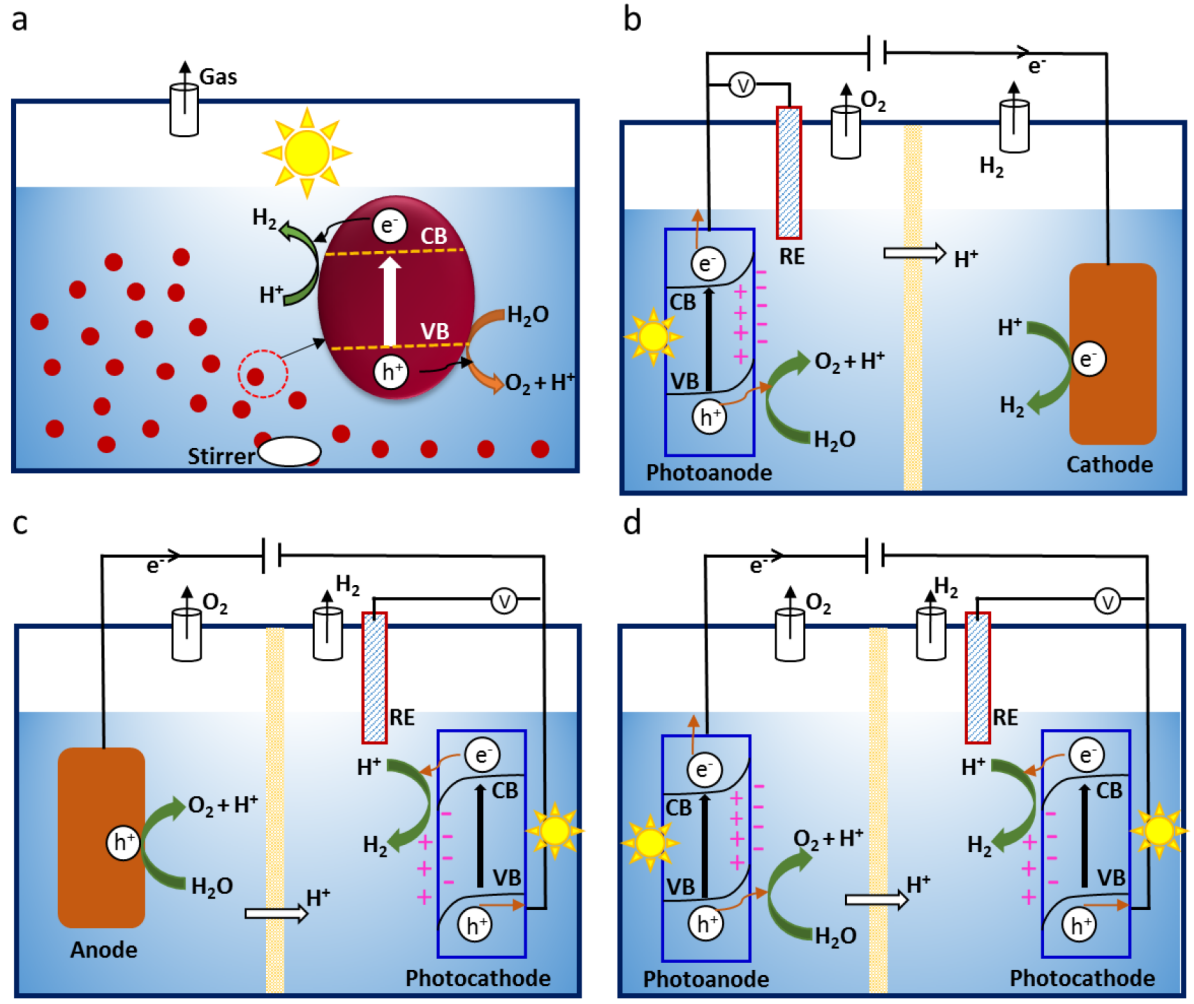
Solar fuel production approaches. (a) Suspension-based photocatalysis. (b) Photoanode|dark cathode. (c) Photocathode|dark anode. (d) Photoanode|Photocathode. Note: For CO_2_ reduction, the same design is applied with the exception of CO_2_ reduction rather than proton reduction on the cathodes. The bias in Figure 1d could be omitted if two photoelectrodes can form a Z-scheme.

Photocatalysis (suspension-based, Figure 1a) is highly desirable as it is driven only by sustainable solar energy, providing the potential to produce H_2_ and high-value chemicals competitively and economically. If the bandgap of the semiconductor is equal to or smaller than the irradiation energy, incoming light irradiation on a photocatalyst powder suspended in an aqueous solution photoexcite the valence band (VB) electrons to the conduction band (CB) of the semiconductor, leaving the corresponding holes in the VB. The photogenerated electrons and holes perform the reduction and oxidation reactions, respectively. Since the first report on photocatalytic water splitting, the single photoabsorber-based system has been vastly studied.^25,26^ In recent times, dual photoabsorber systems, also known as Z-scheme similar to the natural photosystem, have increased in popularity as they have several advantages such as better charge separation, more efficient solar energy harvesting, and the possibility of onsite separation of evolved gases. In such systems, the dual photoabsorbers are combined using an appropriate shuttle redox mediator to regenerate both photocatalysts in a cyclic process. More details about Z-scheme water splitting can be found in our recent review.^27^ Similarly, there are several reports^28−30^ on Z-scheme CO_2_ conversion into value-added fuels. The various Z-scheme approaches, photoreactor designs, and the discussion on large-scale solar fuel production are also available in the literature.^31−34^

The term photoelectrochemical process (PEC) in itself conveys the advantages that are chalked up to both electrocatalysis and photocatalysis. In PEC, a photocatalyst thin film is deposited on a conducting substrate to make what is called a photoelectrode (either the photoanode or photocathode). This is used to absorb the solar energy to generate electron–hole pairs, which can be separated by the internal electric field at the interface. In most cases, an external bias is applied to increase the electric field and help separate charges, in turn increasing the potential of the charges and promoting reactivity. The separated electrons and holes perform the reduction and oxidation reactions, respectively, either directly on the surface of the photocatalyst or on the surface of a cocatalyst.

### 1.2 PEC Approaches

On the basis of the photoelectrode configurations, three PEC approaches *viz*., (i) photoanode|dark cathode, (ii) photocathode|dark anode, and (iii) photoanode|photocathode have been used for solar fuel production. In the photoanode|dark cathode system (Figure 1b), the photoanode acts as the working electrode (WE) for the oxidation half-reaction and a nonphotoactive cathode acts as the counter electrode (CE) for the reduction half-reaction. A stable reference electrode (RE) is also used to control the applied external bias. These electrodes are immersed into the aqueous electrolyte solution, leading to the formation of a semiconductor–electrolyte interface. For the Fermi level of the photoanode to equilibrate with the electron electrochemical potential of the aqueous electrolyte, electrons are transferred from the photoanode (n-type semiconductors) to the electrolyte, which creates a space-charge region also known as an electrical double layer. This built-in potential at the interface induces the upward band bending of the semiconductor. Upon light irradiation, the VB electrons are excited to the CB of the semiconductor and pass to the CE (dark cathode) through an external circuit. These extracted photogenerated electrons perform the reduction reaction at the cathode. The holes left at the VB are transferred to the surface of the photoanode, where an oxidation half-reaction takes place. The n-type inorganic semiconductor-based photoanodes for PEC water splitting and CO_2_ conversion have been vastly reported.^35−41^ Compared with the inorganic counterpart, polymer-based photoanodes have not received the same attention yet and will be addressed in Section 2.

In the photocathode|dark anode approach (Figure 1c), the photocathode acts as the WE and the photoinactive anode as the CE. The rest of the conditions are similar to the photoanode|dark cathode configuration. The Fermi-level difference between the semiconductor and the electrolyte induces the electrochemical potential across the interface, known as a built-in electric field, causing the downward band bending of the semiconductor (p-type photocathode). Upon light irradiation of the photon energy higher than or equal to the bandgap, the photocathode semiconductor electrons are excited to the CB, leaving the corresponding holes in the VB. The built-in potential drives the photogenerated electrons to the surface of the semiconductor followed by interfacial charge transfer to molecules on its surface. An external bias can be applied to facilitate this process. Subsequently, the corresponding holes pass through the external circuit to the CE, where the oxidation reaction takes place. Several inorganic semiconductor-based photocathodes for solar fuel production have been studied and many reviews are available.^4,42,43^ Similarly, polymer-based photocathodes have been little reviewed and will be comprehensively discussed in Section 3.

The photoanode|photocathode configuration (Figure 1d), also known as a tandem cell, consists of an anode and cathode that are both photoactive. Often, n-type and p-type semiconductors are used as the photoanode and photocathode, respectively. If the CB of the photoanode semiconductor is more negative than the VB potential of the photocathode semiconductor, the couple can form a Z-scheme and the external bias is not required. The holes left in the VB of the photoanode and the electrons in the photocathode CB perform oxidation and reduction reactions, respectively. The readers can find more details about the inorganic metal oxide-based tandem-PEC approach for solar water splitting and CO_2_ conversion in the literature,^44−46^ which is beyond the scope of this review.

### 1.3 Polymer-Based Photoelectrodes

One of the main objectives of the polymer (including some examples of organic molecules in this review) based photoelectrodes is to reduce the overall fabrication cost. Standard inorganic materials such as compound semiconductors (groups III–V, Si, etc.),^43^ transition metal oxides (BiVO_4_, Fe_2_O_3_, TiO_2_, and Cu_2_O), sulfides (NiS, CdS), oxynitrides (BaNbO_2_N), and chalcogenides^47−51^ can be expensive and composed of less abundant elements. Next, the fabrication of inorganic photoelectrodes often involves complex deposition techniques such as atomic layer deposition (ALD), high-temperature sintering, electron-beam evaporation, and high energy sputtering, which increase the manufacturing cost and are less straightforward for large-scale production. The synthesis and preparation of polymer-based photoelectrodes are less complex and suitable for preparing large-scale devices as it mostly involves synthetic approaches at ambient conditions, followed by coating on a conductive substrate using low-cost methods such as spin coating, doctor blading, and printing.^52^ Another key advantage of the polymers or organic molecules is that their band positions can be readily tuned, much easier than the inorganic counterparts.^53^

The stability of the photoelectrodes is one crucial factor to consider, given the cost competition with conventional fossil fuel technologies. For instance, photoelectrodes must have a lifetime of 10 years with 10% solar to hydrogen conversion efficiency (STH) to produce H_2_ with a commercially competitive cost of 2–4 USD per kg.^54^ We believe that polymer-based photoelectrodes have the potential to achieve such high stability and efficiency after careful optimization. Organic semiconductors used in photoelectrodes include conducting polymers, oligomers, or self-assembled discrete molecules. Their electronic properties can be tuned more easily than inorganic semiconductors, and hence, diverse materials can be prepared to suit various desired oxidation and reduction reactions. For instance, the photocathode must have the CB potential more negative to the water/CO_2_ reduction potential to produce H_2_/carbon-fuel efficiently. Similarly, the photoanode must have the VB potential more positive than the water oxidation potential of +1.23 V vs RHE to produce O_2_.

In recent years, reports about polymer-based (including organic molecule-based) photoelectrodes for hydrogen evolution reaction (HER), oxygen evolution reaction (OER), and carbon dioxide reduction (CRR) have become more frequent, complementary to the widely used particle suspension system and inorganic counterparts. Many reviews have concentrated on the latter while few of them address the progress, challenges, and prospects of polymer photoelectrodes.^1−4^ Polymer-based photoelectrodes have the advantage of high optical absorption (absorption coefficient 10^5^ cm^–1^)^55^ to harvest solar light efficiently, and their bandgap (absorption spectrum) can be tuned by altering the synthesis conditions to, for example, introduce heteroatoms or change functional groups. Polymer photocatalysts have also shown good charge carrier mobility.^56^ All these advantages have been successfully demonstrated in organic photovoltaics (OPVs).^57^ For instance, the conjugated polymers and self-assembled molecules were substantially reported in OPVs and extended to solar fuel production using an OPV-biased-PEC system.^58,59^

Photoanodes are widely reported for water oxidation reactions, although a limited number of organic semiconductors have been used. This is due to the kinetic complexity of oxidation reactions compared to the water reduction reaction and the typical poor stability of organic photoanodes under oxidation conditions. The use of a polymer-based photoanode for PEC oxidation reaction originated from the modern studies of organic light-harvesting materials for photovoltaic applications. Loading a suitable cocatalyst on the surface of such a polymeric thin film enables photocatalytic water splitting.^60^ The traditional organic molecules such as phthalocyanine derivatives (Pc), and porphyrin/perylene derivatives (Por) have been reported for water oxidation and share structural similarity to the chlorophyll pigments used in natural photosynthesis. Recently, graphitic carbon nitride (CN_*x*_)-based photoanodes have been reported for solar fuel synthesis^61^ spurred by their stability, low cost, and ease of synthesis. However, sluggish water oxidation kinetics and fast charge-carrier recombination during the PEC reaction have compromised their efficiency, leading to poor photocurrent densities. Therefore, it is crucial to use advanced strategies such as intimate contact between the substrate and the film, maintaining porous structure, and doping as described in Section 2 to improve the photocurrent densities of polymer photoanodes.

Polymer-based photocathodes for solar energy conversion appear to be first reported in 1981.^62^ Following that, several conjugated polymers such as polythiophene, polypyrrole (PPy), and polyaniline (PANI)^63,64^ were reported. In particular, an early work on PEC water splitting using a poly(*p*-phenylene)-based photocathode to produce H_2_ was reported by Yanagida et al.,^65^ in which the polymers were prepared by *in situ* electrochemical polymerization using the respective monomers on conducting substrates. Although utilization of polymer-based photocathodes for solar fuel synthesis was pioneered about four decades ago, interest in this area was low for the next two decades. The progress of OPV, the limitations of inorganic metal oxides, and the urgent need for sustainable fuels have triggered the reconsideration of a wide range of materials for solar-driven catalysis, including polymer photoelectrodes. Recently, the fabrication of polymer photocathodes using CN_*x*_,^66^ covalent organic frameworks (COFs),^67^ metal–organic frameworks (MOFs),^68^ conjugated triazine frameworks (CTFs),^69^ and graphene^70^ have been explored for solar fuel synthesis. Notably, tuning the optical and catalytic properties by altering the synthesis conditions dramatically enhanced the PEC performances.^71^ Hence, it is essential to review the latest outcomes on PEC solar fuel synthesis using polymer-based photocathodes and address the challenges, as we do in Section 3.

The PEC activities of the rational polymer-based photoanodes or photocathodes can be enhanced by loading suitable oxidation or reduction electro/molecular catalysts, i.e., cocatalysts.^72^ The cocatalyst layer also protects the substrate underneath^73^ and improves the stability of the photoelectrodes together with the selectivity of the reaction.^74^ Noble metal-based cocatalysts such as Pt,^75^ Au,^76^ Ag,^77^ Pd,^78^ Rh,^79^ and Ir^80^ have been loaded on polymer photoelectrodes for solar fuel synthesis using photodeposition, impregnation, and *in situ* electrodeposition techniques to incorporate the metals into the polymer mix. These cocatalysts are expensive, and some are rare-earth elements and , hence, have limited use for large-scale practical applications. Recently, molecular-based cocatalysts attracted more attention as a suitable alternative to noble metals. Ruthenium (Ru)- and rhenium (Re)-based molecular catalysts, and scalable transition metals catalysts containing manganese (Mn) and cobalt (Co)-based have been progressively used for fabricating efficient polymer-based photoelectrodes. The latest research findings on molecular-based cocatalyst and transition metal-based cocatalysts toward performance improvement of polymer-based photoelectrodes for H_2_ production and CO_2_ reduction reactions have been summarized and presented in Section 4 of this review.

The efficiency of the polymer-based photoelectrodes is mainly determined by the photophysical properties of the polymers and their robustness during the reaction. Specifically, the charge carrier generation, separation, and transfer to the surface, as well as redox reactions at the active sites of the photoelectrodes cumulatively determine the quantum efficiency of the PEC reaction. Hence, a comprehensive understanding of charge carrier dynamics is crucial to the production of solar fuels. Despite several reports on the charge carrier dynamics of inorganic semiconductor-based photoelectrodes for water splitting and CO_2_ reduction, the mechanistic understanding using polymer-based photoelectrodes is rather limited and challenging because of the complex nature of the organic and aqueous interface, which is illustrated in Section 5. The theoritical modelling on polymer-based photoelectrodes is essential to predict the electronic and charge transfer properties of organic semiconductors, which is discussed in Section 6.

Finally, Section 7 concludes with the current understanding of polymer-based photoelectrodes, what are the leading questions that remain to be addressed, and lists out strategies to overcome long-standing challenges in fabricating efficient photoelectrodes, reducing fabrication costs, and increasing the stability.

## 2 Polymer-Based Photoanodes

As mentioned above, solar energy conversion using inorganic metal oxide-based photoanodes such as TiO_2_, SrTiO_3_, TaON, and ZnO for PEC H_2_ production^81^ and BiVO_4_, Fe_2_O_3_, and WO_3_ for PEC water splitting have been reported and reviewed extensively.^82,83^ Recently, earth-abundant elements *viz*. C, N, O, H-based organic semiconductors for solar fuel synthesis have received increasing attention as they are inexpensive. Even though, there are limited examples of polymers used as photoanodes. Here, CN_*x*_-based photoanodes followed by the polymers such as 3,4,9,10-perylenetetracarboxylic acid bisbenzimidazole, cobalt(II) phthalocyanine, poly[benzimidazobenzophenanthroline], and fluorinedibenzothiophene-S,S-dioxide-based conjugated polymer are reviewed. Albeit the polymers can be used as a single photoactive component, fabricating a bulk-heterojunction-based photoanode has more benefits including surface roughness, better charge separation, and increased stability as a photoanode for water splitting. The following subsections describe the different polymer materials being used for fabricating photoanodes.

### 2.1 CN_*x*_-Based Photoanode

**Figure 2:**
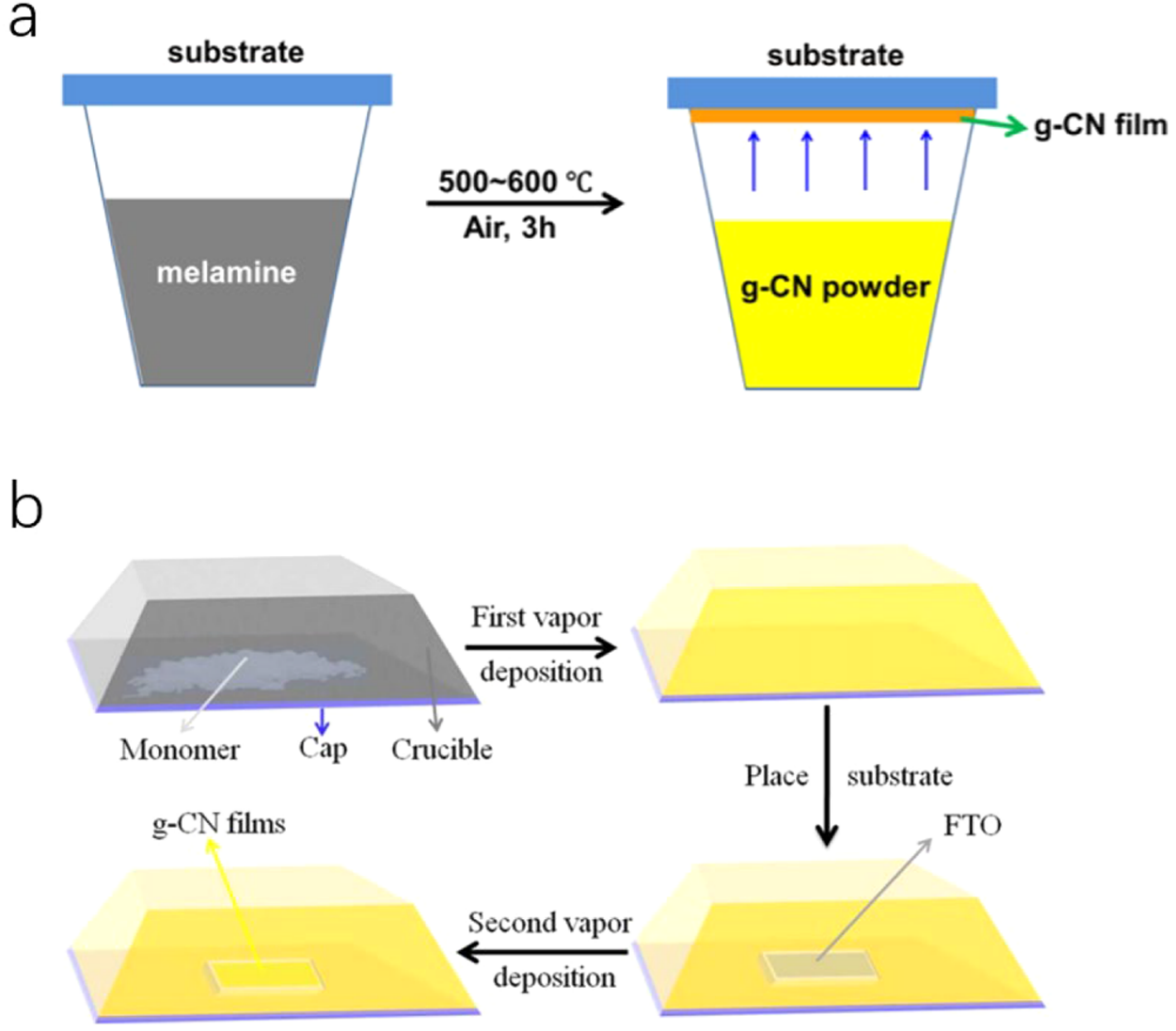
Schematic representation of the synthesis of CN_*x*_ using (a) a thermal vapor condensation method. Adapted with permission from ref (86). Copyright 2015 Elsevier. (b) Two-step vapor deposition method. Adapted with permission from ref (87). Copyright 2017 Elsevier.

Quite different from the suspension-based photocatalyst system, the PEC device relies on directional and long-distance charge transfer across the device. To improve the performance of the PEC system, the electrode/substrate contact, grain boundary contact, electrode surface band bending, and surface defects are crucial elements to be considered while preparing photoelectrodes. For example, CN_*x*_ nanosheets were deposited on FTO substrates via the spray coating method, which simply coated the suspension-like CN_*x*_ onto the substrate without modifying the contact, leading to a low photocurrent density of 0.0036 mA/cm^2^ @ 1.23 V vs RHE.^84^ A similar strategy of exfoliating CN_*x*_ in methanol with postannealing, photoelectrode fabricated afterward resulted in a low photocurrent density of 0.010 mA/cm^2^ @ 1.23 V vs Ag/AgCl as well.^85^ Therefore, a strategy of modifying the contact between the CN_*x*_ film and the substrate is crucial to obtain high photocurrent density. Thermal vapor condensation is a conventional film fabrication method (Figure 2a), which can grow compact g-CN *in situ* on an FTO substrate. Bian et al. used this method to fabricate uniform g-CN films at 600 °C using melamine as a single precursor, showing a photocurrent density of 0.12 mA/cm^2^ @ 1.35 V vs RHE with Na_2_S as the sacrificial reagent under AM 1.5 illumination.^86^ Similarly, two-step vapor deposition (Figure 2b) is an appropriate technique to obtain a CN_*x*_ film. Lv et al. used this process for depositing CN_*x*_ films with dicyanamide and obtained a photocurrent density of 0.063 mA/cm^2^ @ 1.23 V vs RHE under AM 1.5 illumination.^87^ Comparatively, the thermal vapor deposition method also enhances the quality of the CN_*x*_ film by growing CN_*x*_ directly on the substrate. All these factors, including contact between the film and the substrate, microstructure, and other parameters are detailed below to represent their influence on the PEC performance.

#### 2.1.1 Intimate Contact between the Film and the Substrate

The contact between the CN_*x*_ film and a substrate, usually FTO, is a crucial factor in determining the efficiency of the photoelectrode, and hence, several strategies have been used to obtain a better intimate contact. With the use of a facile solvothermal method with postheating, intimate contact between CN_*x*_ and the FTO substrate was achieved.^88^ In a report, the CN_*x*_ powder was deposited as a compact thin film on the SnO_2_ flake by electrophoretic deposition using DC power at a constant voltage of 30 V for 3–10 min.^89^ Prior to this, the SnO_2_ nanoflakes film was chemically treated in a 0.5 M NaOH solution to add OH^–^ surface functional groups which assisted in obtaining a dense and uniform coating of CN_*x*_ nanosheets. This led to a photocurrent density of 0.15 mA/cm^2^ @ 1.23 V vs RHE under AM 1.5 illumination. In another report, the OH^–^ groups on the FTO surface were shown to enable continuous grafting and polymerization of a melamine and cyanuric chloride (1:3 molar ratios) mixture, which was vaporized on FTO at 450 °C for 3 h, and the resultant 200 nm film exhibited a photocurrent density of 0.23 mA/cm^2^ @ 1.23 V vs RHE under AM 1.5 illumination.^90^ Apart from the OH^–^ group, Fang et al. showed that sulfur (S) initialized the intimate growth of CN_*x*_ films on FTO glass.^91^ The CN_*x*_ films synthesized via thermal evaporation show that the S existed at the interfaces between the CN_*x*_ and FTO, which was confirmed by X-ray photoelectron spectroscopy (XPS). The S facilitated charge migration between CN_*x*_ and the substrate. As a result, it contributed to an improved photocurrent (compared to the absence of S) of ca. 0.1 mA/cm^2^ at 1.23 V vs RHE under AM 1.5 illumination.

#### 2.1.2 Microstructure Control

The control of the microstructure of the film is another crucial factor to determine the efficiency of the photoelectrodes. In general, the photoelectrode fabrication process gives two types of film, i.e., compact or porous films. The compact film has fewer surface defects, higher crystallinity, controlled thickness, and a large electron transport distance.^92^ On the other hand, the porous structure has a short electron diffusion distance, large surface area, more active sites, and efficient light absorption.^93^ We discuss a few examples below to highlight the role of compact and porous CN_*x*_ films on PEC performance.

##### Compact Film

**Figure 3:**
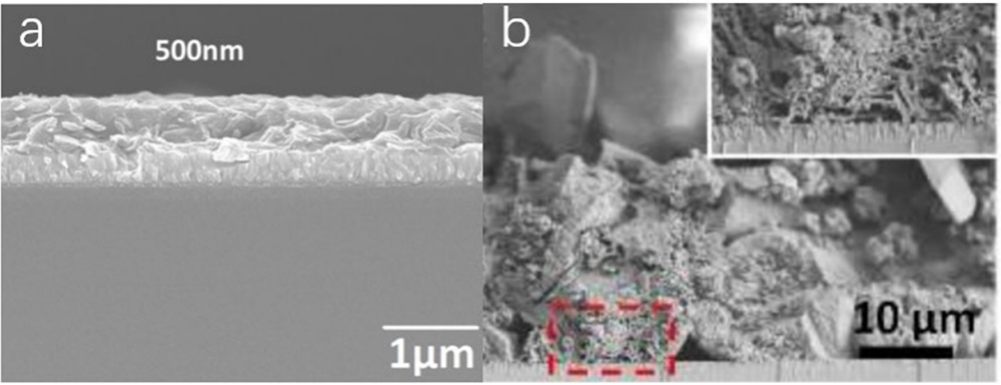
(a) Side view of compact CN_*x*_ film. Adapted with permission from ref (94). Copyright 2019 Royal Society of Chemistry. (b) Cross-section SEM image of the CN_*x*_ film. Adapted with permission from ref (95). Copyright 2018 John Wiley & Sons, Inc.

Ruan et al. reported a rapid thermal evaporation–condensation method to prepare high-quality, compact CN_*x*_ films, with controlled thickness from 500–1000 nm as shown in Figure 3a. Due to the reduced deep trap states, the film achieved a high open-circuit photovoltage of 0.3 V, which was much higher than the photovoltage obtained (0.04 V) for a porous CN_*x*_ film prepared by a traditional thermal evaporation method. Transient photovoltage measurements revealed that the electron diffusion length was nearly 1000 nm for a compact film and obtained a high photocurrent density of 0.180 mA/cm^2^ at 1.23 V vs RHE with a 150 W xenon lamp.^94^

Peng et al. also demonstrated a simple and versatile method to grow crystalline CN_*x*_ films with a closely packed layered structure on FTO via the seeded crystallization of CN monomers followed by their calcination at high temperature. Upon calcination, a strongly bonded CN_*x*_ layer on FTO was successfully obtained as shown in Figure 3b, with a thickness of roughly 30 μm.^95^ The resultant CN_*x*_ film exhibited impressive PEC performance with a photoanodic photocurrent of 0.116 mA/cm^2^ at 1.23 V vs RHE and up to a 1 V shift of the onset potential under one sun in 0.1 M KOH aqueous solution. Furthermore, IPCE values at 400 and 420 nm reached 8.5% and 3.6%, respectively. In summary, the method for synthesizing a compact film could improve the contact between the film and the substrate, thus enhancing charge transfer. However, increasing the film thickness beyond 30 μm led to the reduction in the photocurrent density to ∼0.1 mA/cm^2^ at 1.23 V vs RHE. Hence, it is worth noting that while preparing a compact film, the thickness of the film also must be considered.

##### Porous Film

Porous photoelectrode usually provides a short electron diffusion distance, large surface area, and a large number of active sites. A simple and versatile doctor blade method could be used to fabricate large-scale and highly porous CN_*x*_ films with controllable thickness, which can be transferred onto various substrates ranging from FTO, Al foil, porous TiO_2_, silicon wafer, and glass.^96^ Upon calcination at 550 °C for 4 h, the uniform CN_*x*_ layers with the high surface area were obtained according to the electrochemical surface area and dye adsorption measurements. Such a porous film (CN_*x*_/FTO) exhibited excellent dye degradation and some PEC performance of ∼0.012 mA/cm^2^ under white-light illumination at 1.23 V vs RHE in 0.1 M KOH.^96^ The obtained low photocurrent density may be due to the poor electrode-substrate contact. Similarly, highly porous CN_*x*_ layer/reduced graphene oxide (rGO) films on FTO substrate were prepared by using a doctor blade method. In this configuration, rGO acted as a support for the CN_*x*_ layer growth. The obtained film thickness was ∼60 μm. The PEC studies reveal that the rGO layer significantly improved the charge transfer and increased the electrochemically active surface area, leading to a dramatic enhancement of the PEC performance with a photocurrent of 0.072 mA/cm^2^ at 1.23 V versus RHE in a 0.1 M KOH solution and an external quantum efficiency (EQE) of 5.3% at 400 nm under one-sun illumination.^97^

**Figure 4:**
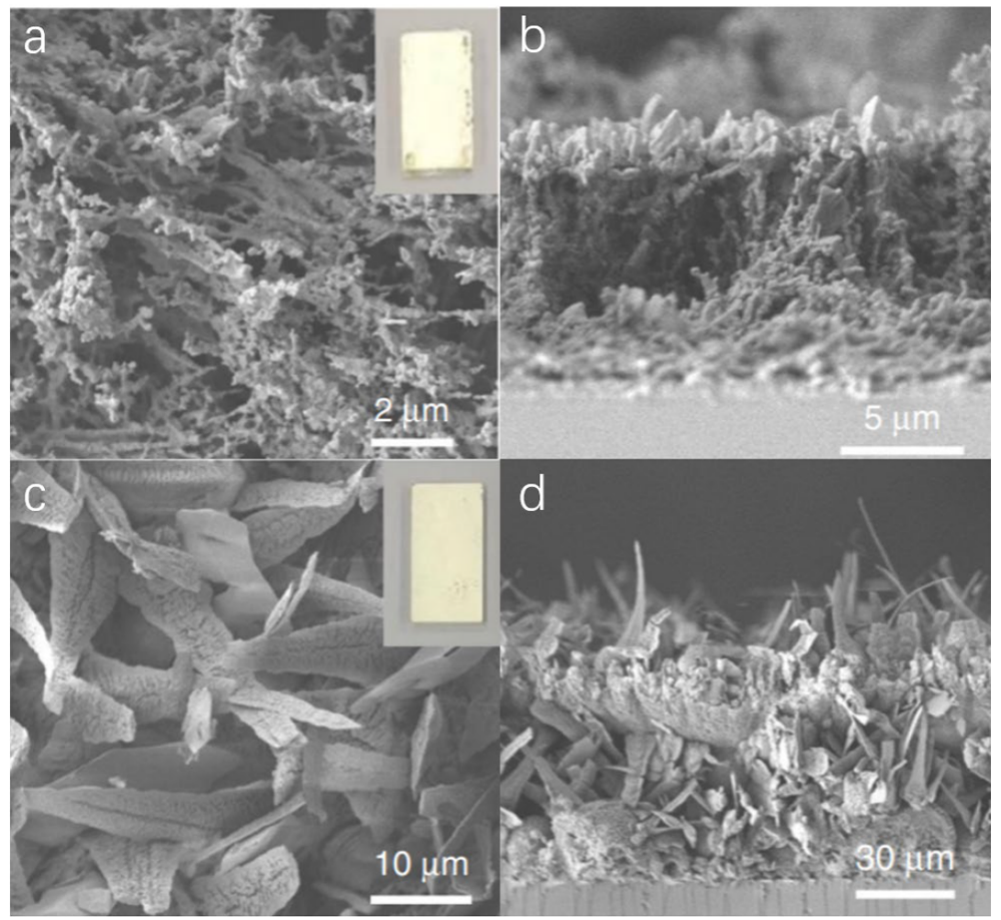
Top (a,c) and side (b,d) views of porous CN_*x*_ film. Adapted with permission from ref (98). Copyright 2020 Nature Springer.

The photocurrent density of the porous photoelectrodes can be enhanced by improving the intimate contact between the electrode/substrate and the porous photoactive layer. In a report, a robust method was used to rapidly grow CN_*x*_ monomers directly from a hot saturated solution of thiourea on various substrates. Upon calcination, a uniform CN_*x*_ layer with tuned structural and photophysical properties was obtained including the intimate contact with the substrate as shown in Figure 4. The film thickness was ranged from 10 to 50 μm. The detailed PEC and structural studies revealed good photoresponse up to 600 nm, excellent hole extraction efficiency (up to 62%), and strong adhesion of the CN_*x*_ layer to the substrate. The best CN_*x*_ photoanode demonstrated a benchmark photocurrent density of 0.353 mA/cm^2^ (51% faradaic efficiency for oxygen) and an EQE of 12% at 450 nm at 1.23 V versus RHE in an alkaline solution.^98^ Conclusively, this high performance is benefited from the porous structure and intimate contact between CN_*x*_ and FTO. A widely reported trend observed with CN_*x*_-based photoelectrodes, similar to their inorganic counterparts such as hematite and BiVO_4_, is that porous films likely possessed better performance than compact films.^99^ On the other hand, preparing highly crystallized CN_*x*_ photoelectrodes or single crystals with superior charge transfer ability is very challenging although it is more efficient.

#### 2.1.3 Other Strategies

In addition to the above facts, a few other strategies such as doping or structure modification also play a significant role in the optimization of the PEC charge transfer. Poly melamine-formaldehyde resin (PMF) (formed by reacting melamine and formaldehyde) was used as a precursor to dope carbon atoms into the tri-s-triazine units at 500 °C. The layer arrangement disappeared while tri-s-triazine repeating units became prominent in X-ray diffraction (XRD) patterns. The resultant film showed a high photocurrent density of 0.23 mA/cm^2^ at 1.23 V vs RHE under AM 1.5 illumination, which was believed to be associated with improved charge transfer kinetics in the bulk.^100^ CN_*x*_ was also doped with boron (B) using boric acid via a thermal vapor deposition method. The B atom incorporation primarily improved the transport of the minority charge carriers (holes) within the semiconductor, which was four times higher than that of the pristine CN_*x*_. In the presence of Na_2_S as a hole scavenger, the IPCE was nearly 4% at 400 nm with a photocurrent density of 0.055 mA/cm^2^ at 1.23 V vs RHE under AM 1.5 illumination.^101^ In another report, Zhang et al. doped CN_*x*_ with phosphorus (P) using BmimPF_6_ as the source by polycondensation and achieved 4 orders of magnitude enhanced electrical conductivity and five orders magnitude improved photocurrent generation compared to pristine CN_*x*_ due to the introduction of intermediate states between VB and CB and more efficient light harvesting.^102^ Xu et al. demonstrated a comelting strategy by combining molten sulfur and supramolecularly aligned liquid intermediates for *in situ* fabrication of phenyl-substituted CN_*x*_ (PhCN_*x*_) thin films. Photophysical studies revealed that a sub-band likely formed from oriented phenyl subarrays within the bandgap, which assisted the trapping of photogenerated holes, thus reducing charge recombination and promoting hole transfer to the electrolyte. The sum of improved optical absorption, electronic conductivity, and hole transfer synergistically increased the photocurrent by a factor of 20 under visible light illumination compared to the nonsulfur processed analogue.^103^ Ruan et al. reported a novel nanojunction architecture that was composed of a B-doped CN_*x*_ nanolayer and bulk CN_*x*_. It was fabricated by a rapid thermal evaporation quenching method. The top layer of the nanojunction had a depth of ca. 100 nm, and the bottom layer was ca. 900 nm. The nanojunction photoanode resulted in a 10-fold higher photocurrent than bulk CN_*x*_ with an excellent photocurrent density of 0.103 mA/cm^2^ at 1.23 V vs RHE under one sun condition and an extremely high IPCE of ca. 10% at 400 nm.^104^ The tightly packed CN_*x*_ layer prepared by depositing a supramolecular complex comprising melamine-bismuthiol blended with rGO (MSG) on FTO was found to improve the electron diffusion within the CN_*x*_ and hole extraction to the solution.^105^ A type-II heterojunction was then formed by depositing a second layer of CN_*x*_ using the melamine precursor (CN_*x*_-M) by thermal vapor condensation. The resulting FTO/CN_*x*_-MSG/CN_*x*_-M photoanode demonstrated a very high photocurrent density of 0.270 mA/cm^2^ in 0.1 M KOH solution at 1.23 V vs RHE.

### 2.2 Other Polymers (Including Organic Molecules)-Based Photoanodes

**Figure 5:**
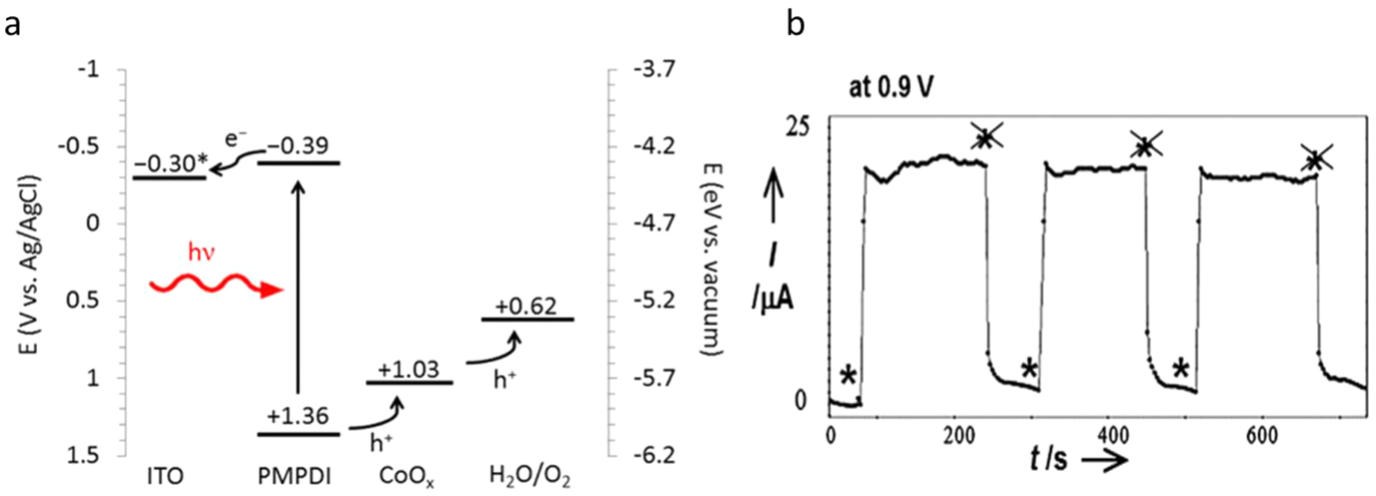
(a) Band diagram for the ITO/PMPDI/CoO_*x*_ system. Adapted from ref (107). Copyright 2014 American Chemical Society. (b) Currents with and without illumination, by PTTh-2/ITO glass in 0.1 M Na_2_SO_4_ at 0.9 V vs Ag/AgCl. Stars signify “light on”. Stars with crosses through them indicate, “light off”. Adapted with permission from ref (109). Copyright 2012 Wiley-VCH.

Decades ago, a thin film of chlorogallium phthalocyanine on a Au electrode was reported for PEC H_2_ evolution with an efficiency of 0.1%, and the enhanced activity was observed in the presence of Pt cocatalyst.^106^ Thereafter, a thin film of perylene diimide functionalized with phosphonate groups, *N*,*N*′-bis(phosphonomethyl)-3,4,9,10-perylenediimide (PMPDI), coated on an ITO photoanode was reported for water oxidation, in which CoO_*x*_ was used as a cocatalyst.^107^ Under visible-light irradiation, the ITO/PMPDI/CoO_*x*_ electrode produced a water oxidation photocurrent density of 0.150 mA/cm^2^ at 1.0 V applied bias vs Ag/AgCl with a FE of 80 ± 15% and internal quantum efficiency of 1% for O_2_ evolution. Figure 5a shows the working mechanism of the ITO/PMPDI/CoO_*x*_ photoanode for water oxidation. Recently, an n-type conjugated polymer poly[benzimidazobenzophenanthroline] (BBL)-based photoanode was prepared with exceptional stability and good electron mobility up to 0.1 cm^2^ V^–1^ s^–1^. The BBL polymer film was coated on an FTO substrate using the dispersion-spray method, which showed a morphology-dependent performance. In the presence of the hole acceptor (SO_3_^2–^), the BBL photoanode displayed photocurrents up to 0.23 ± 0.02 mA/cm^2^ at 1.23 V vs RHE under standard simulated solar illumination.^108^ The photocurrent was further enhanced to 0.26 ± 0.02 mA/cm^2^ by functionalizing the photoanode with 1 nm of TiO_2_ followed by a nickel–cobalt catalyst.

Earlier, porphyrins and metalloporphyrins were used for homogeneous photocatalysis to decompose organic pollutants in the contaminated water and air.^110^ However, it was limited to short time use because of the poor stability of the porphyrin molecules.^111^ Later, this issue was resolved by depositing the porphyrin molecules onto solid supports or integrating them with robust nanostructures. One early report on water oxidation using manganese porphyrin (MnPor) came in 1994, in which dimanganese complexes were obtained by linking the triphenylporphyrin (TPP) by an o-phenylene bridge.^112^ The corresponding photoanode fabricated using MnPor showed PEC water oxidation with a FE of 5–17% at an applied bias of 1.2–1.5 V. MnPor was also used as a cocatalyst for enhancing the PEC water oxidation reaction on polythiophene. The incorporation was achieved by oxidative electrochemical polymerization of terthiophene to poly(terthiophene) (PTTh) on ITO substrates in ethanol–dichloromethane (1:1) solution, in the presence of dissolved 5,10,15,20-tetra(4-sulfonophenyl) porphyrin tetrasodium salt.^109^ The resulting ITO/PTTh:MnPor photoanode showed a photocurrent of 0.023 mA (not normalized for the surface area) at +0.9 V vs Ag/AgCl (+1.51 V vs RHE) in a 0.1 M Na_2_SO_4_ (pH 7) electrolyte solution under the illumination of a halogen lamp (Figure 5b). Furthermore, it was interesting to see seawater oxidation with high selectivity for O_2_ in this study.

**Figure 6:**
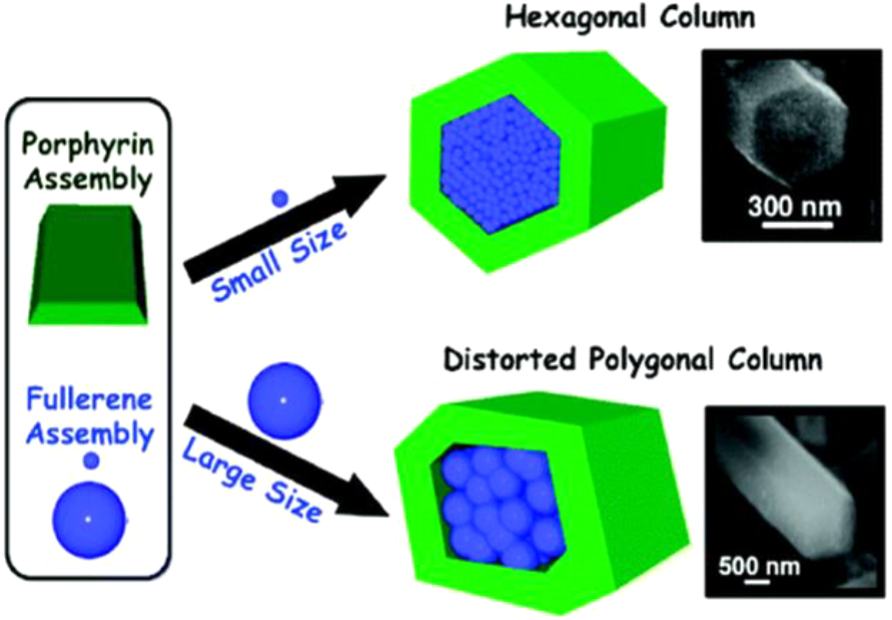
Schematic representation of the formation of the hexagonal C_60_-ZnP(Py)_4_ rod and the distorted polygonal C_60_*t*Bu-ZnP(Py)_4_ rod. Adapted from ref (113). Copyright 2009 American Chemical Society.

Structurally controlled zinc *meso*-tetra(4-pyridyl)porphyrin [ZnP(Py)_4_] nanorods were prepared by encapsulating fullerene derivatives (C_60_, C_60_ derivatives, and C_70_) by a solvent mixture technique in the presence of surfactant molecule cetyltrimethylammonium bromide (CTAB) in a DMF/acetonitrile mixture. Hexagonal nanotubes of ZnP(Py)_4_ with a large hollow structure was obtained, which became a nanorods shape while combining with fullerenes (Figure 6).^113^ Time-resolved fluorescence spectra showed efficient fluorescence quenching, suggesting the forward electron-transfer process from the singlet excited state of ZnP(Py)_4_ to fullerenes. The SnO_2_/fullerene-ZnP(Py)_4_ photoanode exhibited a photocurrent density of 1 mA/cm^2^ at 0 V vs RHE, the solar energy conversion efficiency of 0.63%, and an IPCE of 35%. One organic bilayer composed of 3,4,9,10-perylenetetracarboxylic acid bisbenzimidazole (PTCBI, an n-type semiconductor) and cobalt phthalocyanine (CoPc, a p-type semiconductor) prepared by vapor deposition was also reported for PEC water oxidation. This organic photoanode (ITO/PTCBI/CoPc) exhibited the water oxidation photocurrent density of 0.02 mA/cm^2^ at 1.2 V vs RHE.^114^ To improve the stability of the n-type organic fullerene derivative, PC_71_BM ([6,6]-phenyl C_71_ butyric acid methyl ester), an ultrathin ZnO (<2 nm) passivation layer was deposited with controlled thickness. The photogenerated holes from the PC_71_BM were efficiently transferred to the electrolyte through the ZnO passivation layer resulting in the photocurrent density of 0.06 mA/cm^2^ at 1.23 V vs RHE.^115^

A bulk heterojunction (BHJ) between p-type poly[(2,6-(4,8-bis(5-(2-ethylhexyl)thiophen-2-yl)-benzo[1,2-*b*:4,5-*b′*]dithiophene))-*alt*-(5,5-(1*′*,3*′*-di-2-thienyl-5*′*,7*′*-bis(2-ethylhexyl)benzo[1*′*,2*′*-*c*:4*′*,5*′*-*c′*]dithiophene-4,8-dione) (PBDB-T) and n-type 3,9-bis(2-methylene-(3-(1,1-dicyanomethylene)-indanone))-5,5,11,11-tetrakis(4-hexylphenyl)-dithieno[2,3-*d*:2*′*,3*′*-*d′*]-s indaceno[1,2-*b*:5,6-*b′*]dithiophene (ITIC) was reported for PEC water oxidation. This BHJ photoactive layer was preserved by nickel–iron-layered double hydroxides (NiFe-LDHs) to enhance the charge-separation efficiency and reduce the photocorrosion of the organic layer. In addition, GaIn was used as a mediator between BHJ and Ni foil for efficient charge transfer. This photoanode (ITO/PBDB-T/ITIC/GaIn@Ni/NiFe-LDHs) exhibited a record water oxidation photocurrent density of 15.1 mA/cm^2^ at 1.23 V vs RHE.^116^ Similarly, a BHJ-based photoanode was prepared using a covalent polymer network (CPN) and SnO_2_, and the photocurrent density of 3.3 mA/cm^2^ at 0.54 V vs RHE was obtained at pH 0.^117^ In addition, a BHJ made up of benzodithiophene-based polymer PBDTTTPD and naphthalenediimide-based polymer PNDITCVT was reported for PEC water oxidation. The BHJ after loading water oxidation catalyst Co_3_O_4_ showed a photocurrent density of 2 mA/cm^2^ at 1.23 V vs RHE at pH 9.0. In this study, the charge accumulation on the photoanode was found to be a big concern for stability. To overcome this issue, a hole extraction layer of poly(triaryl amine) (PTAA) was deposited, which facilitated the charge extraction and reduced the charge accumulation. This FTO/mZnO/PBDTTTPD-PNDITCVT/Co_3_O_4_/PTAA/LiO photoanode displayed a slightly improved photocurrent density to 2.3 mA/cm^2^ at 1.23 V vs RHE, more importantly at a low onset potential of 0.2 V vs RHE and an IPCE of 25%.^118^

Overall, the limited results on polymer photoanodes (Table 1) were reported, and the highest photocurrent density of 15.1 mA/cm^2^ at 1.23 V vs RHE was achieved on the ITO/PBDB-T/ITIC/GaIn@Ni/NiFe-LDHs photoanode,^116^ which is comparable to that reported on inorganic counterpart photoelectrodes.

**Table 1:** Polymer and Representative Organic Molecules-Based Photoanodes and Their PEC Performances

photoanode design	photocurrent density (mA/cm^2^ vs RHE)	PEC reaction	pH	reference
FTO/CN_*x*_ (TVC)	0.12 at 1.55 V	water splitting	11.6	(86)
FTO/SnO_2_/CN_*x*_ (EPD)	0.15 at 1.23 V	water splitting	7.0	(89)
FTO/2D-CN_*x*_	0.23 at 1.23 V	water splitting	7.0	(90)
FTO/S-CN_*x*_	0.1 at 1.23 V	water splitting	13.0	(91)
FTO/CN_*x*_ (porous)	0.18 at 1.23 V	water splitting	7.0	(94)
FTO/CN_*x*_	0.116 at 1.23 V	water splitting	13.0	(95)
FTO/CN_*x*_ (porous)	0.353 at 1.23 V	water splitting	7.0	(98)
FTO/CN_*x*_	0.23 at 1.23 V	water splitting	6.0	(100)
FTO/s-BCN	0.102 at 1.23 V	water splitting	6.6	(104)
ITO/PMPDI/CoO_*x*_	0.150 at 1.56	water splitting	7.0	(107)
OTE/SnO_2_/C_60_-ZnP(Py)_4_	1.0 at 1.23 V	water splitting	7.0	(113)
FTO/CN-MSG/CN-M	0.270 at 1.23 V	water splitting	13.5	(105)
ITO/PBDB-T/ITIC/GaIn@Ni/NiFe-LDHs	15.1 at1.23 V	water splitting	13.6	(116)
FTO/CPN:SnO_2_	3.3 at 0.54 V	HI splitting	0	(117)
FTO/mZnO/PBDTTTPD-PNDITCVT/Co_3_O_4_	>2 at 1.23 V	water splitting	9	(118)
FTO/[Ru(bpy)_2_(4,4- (PO_3_H_2_)_2_bpy)]Br_2_/TiO_2_	>1.7 at 0.2 V	water splitting	6.8	(119)

## 3 Polymer-Based Photocathodes

In this section, the emerging polymer-based photocathodes for water and CO_2_ reduction reactions are reviewed. As explained in the previous section, although single component polymer photocathodes were used for PEC reduction reaction, it is significant to engineer the BHJ to efficiently separate the charge carriers for catalysis. For a polymeric photocathode, it must have good stability when in direct contact with electrolytes. Next, the reduction potential of the polymer, which is approximated by the CB edge, should be more negative than water or CO_2_ reduction potentials to ensure thermodynamically favored electron transfer. Although polymer semiconductors are good light-absorbing materials, the efficient charge transfer to the surface is challenging because of competitive recombination. The following reviewed the different polymer semiconductors meeting these requirements.

### 3.1 CN_*x*_-Based Photocathode

Although CN_*x*_ has been used as a photoanode, thermodynamically its VB is aligned around +1.6 V vs RHE, which is not very favorable for water oxidation (+1.23 V vs RHE) due to the small overpotential. This could explain the existing gap in PEC performance between CN_*x*_ and traditional metal oxide photoanodes (TiO_2_, BiVO_4_, WO_3_, Fe_2_O_3_, and TaON)^120^ which have more positive VB edges. Considering the negative CB (−1.1 V vs RHE) and the great success of CN_*x*_ for photocatalytic H_2_ evolution in a suspension system, CN_*x*_-based photocathodes are potentially more attractive than photoanodes. Intrinsically, CN_*x*_ is a n-type semiconductor that generates an upward band bending on the surface and exhibits anodic photocurrent in most studies. To construct a photocathode using an n-type semiconductor, it must overcome the depletion layer filled with holes at the interface, which can be achieved by tuning the electronic properties of CN_*x*_ by heteroatoms doping. For instance, B doping showed a cathodic current while with a limited current density of 0.01 mA/cm^2^ at −0.2 V vs RHE for CO_2_ reduction, due to the positively shifted conduction band (−0.44 V vs RHE) after B doping, therefore mitigating the reduction ability.^121^ Likewise, Zhang et al. developed phosphorus (P)-doped CN_*x*_ as a photocathode and observed an accelerated charge transfer in the bulk and an enhanced IPCE of 1.5% at 400 nm at −0.2 V bias vs Ag/AgCl.^102^ Though the P atoms doping changed the electronic structure of CN_*x*_, the PEC efficiency was rather low.

**Figure 7:**
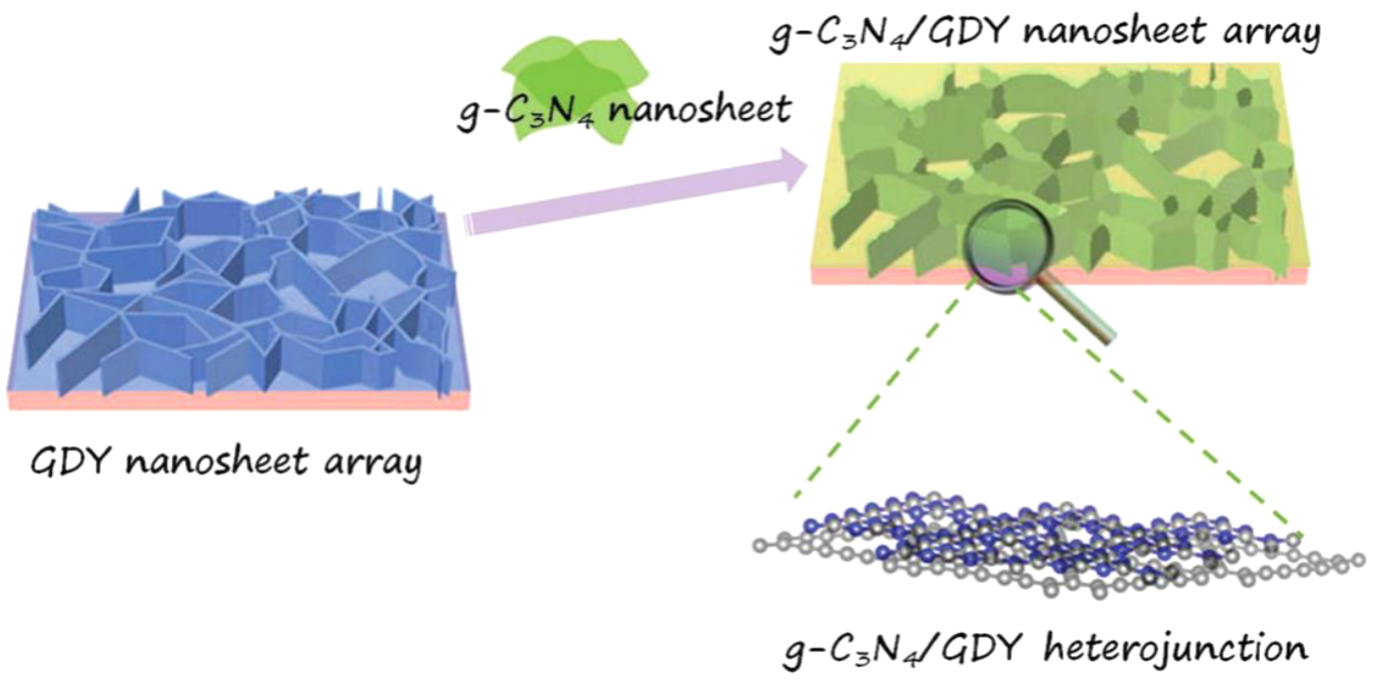
Schematic illustration for the construction of 2D/2D CNx/graphdiyne heterojunction on a 3D GDY nanosheet array. Adapted with permission from ref (123). Copyright 2018 Wiley-Blackwell.

To improve the photoreduction performance of the CN_*x*_ cathode, it has to be changed from an n-type to a p-type. Ruan et al. substituted NH_2_ terminals of CN_*x*_ with −OH groups, which introduced sufficient surface shallow trap states for electrons, extending the lifetime of trapped electrons up to 1 μs for PEC H_2_ evolution.^122^ Further by combining the CN_*x*_ layer with a graphdiyne nanolayer on Cu foil (Figure 7), an efficient photocathode was fabricated to fasten the hole extraction from CN_*x*_ to graphdiyne/Cu substrate, and the electrons left on the CNx performed the reduction reaction. This photocathode produced a photocurrent density of 0.133 mA/cm^2^ at a potential of 0 V vs RHE in a neutral aqueous solution.^123^

**Figure 8:**
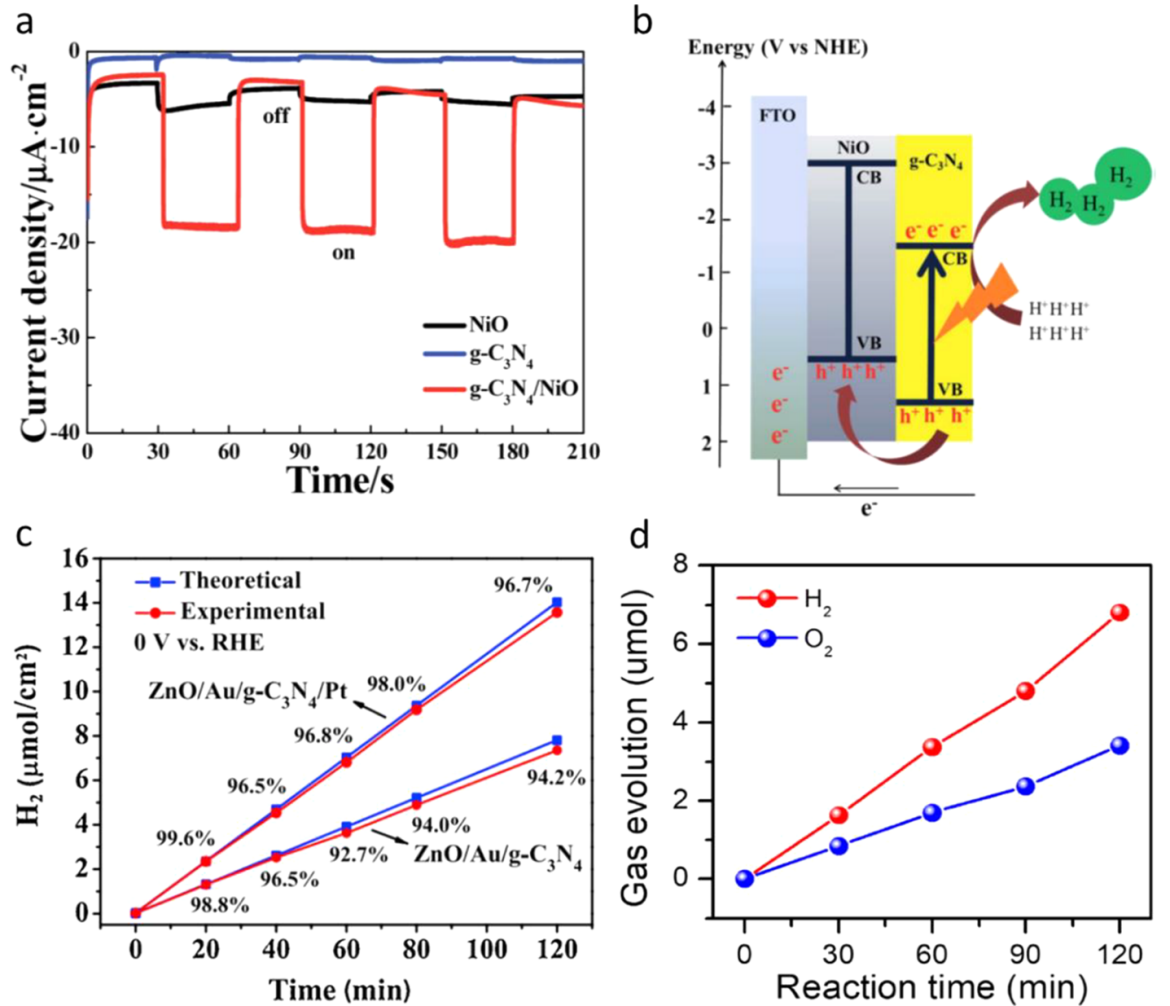
(a) Current density vs time of the three electrodes under chopped light illumination in 0.10 M Na_2_SO_4_ solution at a bias potential of 0 V vs RHE. (b) The mechanism of PEC H_2_ generation using the g-C_3_N_4_/NiO electrode. Adapted with permission from ref (126). Copyright 2016 Royal Society of Chemistry. (c) Hydrogen production and faradic efficiency of 3D urchin-like ZnO/Au/g-C_3_N_4_ and Pt-loaded 3D urchin-like ZnO/Au/g-C_3_N_4_ photocathodes. Adapted with permission from ref (127). Copyright 2020 Elsevier. (d) H_2_ and O_2_ evolution of the Cu-CN-W photocathode at 0.42 V vs RHE using Pt as the counter electrode. Adapted with permission from ref (128). Copyright 2019 Wiley-VCH.

The CN_*x*_ nanosheets on NiO arrays were reported to form a photocathode with a photocurrent density of 70 μA/cm^–2^ at 0.42 V vs RHE.^124^ It was believed that the porous structure of CN_*x*_ or CN_*x*_ deposited on aporous conductive substrate would contribute to the photocathode characteristic, similar to minimizing the band bending in a suspension system. The sufficient surface trap states for electrons present in the CN_*x*_ easily converted it into a photocathode, whereas traditional metal oxide’s porous structure showed photoanode characteristics.^125^ Similarly, Dong et al. reported a CN_*x*_/NiO photocathode for PEC H_2_ evolution,^126^ in which the NiO was used to extract the holes from the CN_*x*_. The CN_*x*_/NiO photocathode showed a photocurrent density of 0.020 mA/cm^2^ at 0 V vs RHE, which is 10 times higher than the bare NiO and 20 times the bare CN_*x*_ (Figure 8a).

Interestingly, this photocathode was producing H_2_ with 100% FE and high stability. A low charge transfer resistance for CN_*x*_/NiO photocathode was observed, indicating that it was appropriate for electron transfer to the electrolyte for the proton reduction reaction. The photogenerated holes at the VB of the CN_*x*_ transferred to the NiO VB, preventing the recombination with CN_*x*_ CB electrons (Figure 8b). A solid Z-scheme photoelectrode ZnO/Au/CN_*x*_ was reported for photocathodic performance.^127^ The addition of the Au layer acted as a solid electron mediator to promote the electron transfer between the ZnO and CN_*x*_. With the use of this photocathode, an H_2_ evolution rate of 3.69 μmol h^–1^ cm^–2^ with a FE of 95.2% was obtained under one sun irradiation at an applied bias of 0 V vs RHE in 0.1 M Na_2_SO_4_, indicating that the photoelectrons generated by the photocathode were mainly used for the proton reduction reaction. After Pt cocatalyst loading on the surface of the CN_*x*_, the H_2_ evolution rate was increased to 6.75 μmol h^–1^ cm^–2^ (Figure 8c) and a FE of 97.5% was achieved. In another report, Basu et al. prepared CN_*x*_ embedded CoSe_2_ using a combustion technique followed by a simple hydrothermal route to reduce the charge accumulation on the CoSe_2_, which led to increased stability.^129^ CN_*x*_-CoSe_2_ ink was prepared to fabricate the PEC H_2_ evolution photocathode by placing them on top of the p-Si microwires, in which CN_*x*_-CoSe_2_ was used as an electrocatalyst. It showed a photocurrent density of 4.89 mA/cm^2^ at 0 V vs RHE and an H_2_ evolution rate of 1.77 μmol min^–1^ with a FE of 80%.

Copper (Cu)-modified CN_*x*_ was reported as an effective photocathode for solar water splitting. The special synthesis method introduced free CuCl and Cu into CN_*x*_, which formed the heterojunction between Cu and CN_*x*_, similar to a type-II junction leading to enhanced photocurrent density for H_2_ evolution.^128^ This work highlighted the molten-salt-based synthesis as an alternative to the liquid-phase synthesis as the molten salt-based approach provides high crystallinity. It produced the cathodic photocurrent density of 0.200 mA/cm^2^ at 0.42 V vs RHE and the H_2_ evolution rate of 3 μmol h^–1^ with a FE of 90.6% (Figure 8d). The mechanistic study revealed that the photogenerated electrons from the CN_*x*_ transferred to the CB of the CuCl followed by injection into the protons for H_2_ evolution.

### 3.2 Covalent Organic Framework (COF) and Metal–Organic Framework (MOF)-Based Photocathodes

COFs are newly developed low-density crystalline polymers that consist of organic units linked via covalent bonds to form porous networks. The selection of appropriate building blocks and linkage motifs provides ways to tailor the optical and electronic properties of COF structures. It has some special potentials for the PEC process such as excellent visible light absorption, fast charge separation and transfer thereby less recombination, and good thermal and chemical stability. Though COFs have been vastly reported as particulate photocatalysts for solar fuel synthesis,^130,131^ the COFs-based photocathode is also attractive.

**Figure 9:**
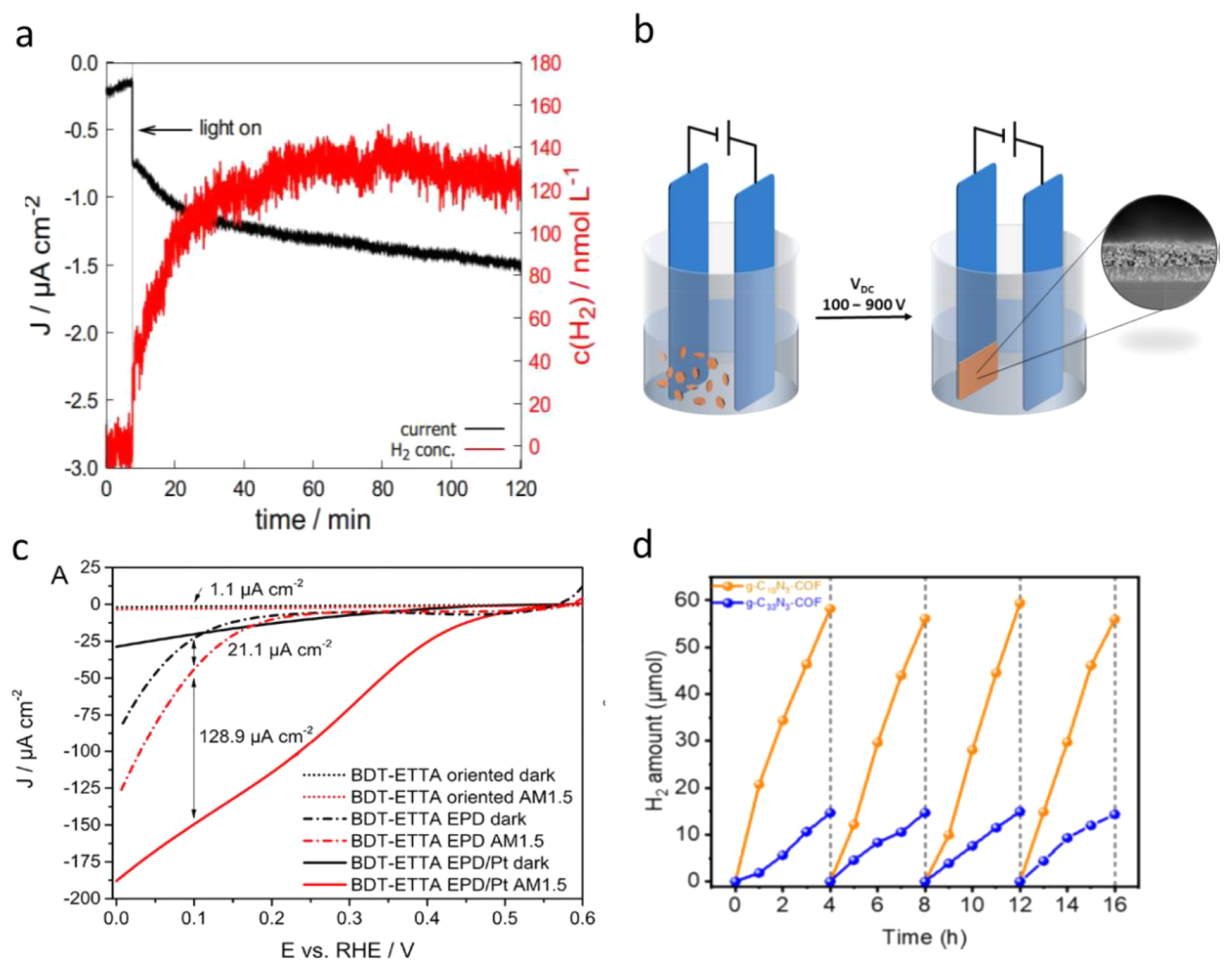
(a) Hydrogen evolution on a BDT-ETTA COF electrode was quantified with a hydrogen microsensor (*Unisense A/S H2-NPLR*) with a selective silicone membrane at a static potential of 0.4 V vs RHE. Illumination of the sample with AM1.5 simulated sunlight results in a photocurrent (black) and the production of hydrogen (red). Adapted from ref (132). Copyright 2018 American Chemical Society. (b) Schematic presentation of the EPD setup with a typical COF film SEM cross-section. (c) Dynamic hydrogen evolution measurement under chopped AM1.5G illumination of a BDT-ETTA COF electrode at 0.2 V vs RHE. Adapted from ref (133). Copyright 2019 American Chemical Society. (d) Time course hydrogen evolution using g-C_18_N_3_-COF and g-C_33_N_3_-COF as catalysts under visible light (λ > 420 nm) irradiation, monitored over 16 h with evacuation every 4 h (dashed line). Adapted from ref (134). Copyright 2019 American Chemical Society.

An imine-based COF photocathode prepared by using aromatic amine-functionalized tetraphenylethylene (1,1′2,2′-tetra-*p*-aminophenylethylene, ETTA) and thiophene-based building blocks (benzo[1,2-b:4,5-b′]-dithiophene-2,6-dicarboxaldehyde, BDT) was reported for the PEC H_2_ evolution. The HOMO and LUMO bandgap measured using the UV–vis and PEC measurements revealed that the LUMO of the COF has higher energy than the water reduction potential. It exhibited a small cathodic photocurrent density of 0.0043 mA/cm^2^ at +0.3 V vs RHE under one sun irradiation (Figure 9a),^132^ which was further enhanced by a factor of 4 after loading Pt nanoparticles. In the follow-up study, the same group used a similar COF film but prepared it by an electrophoretic deposition approach (Figure 9b),^133^ which showed an HER photocurrent density of 0.021 mA/cm^2^ at 0.1 V vs RHE under the same experimental conditions. By depositing the Pt cocatalyst, the photocurrent density was increased significantly to 0.128 mA/cm^2^ at 0.1 V vs RHE (Figure 9c). In another report, a new type of COF was synthesized using the Knoevenagel condensation approach in which sp^2^-carbon-linked triazine core 2D sheets were vertically stacked into high crystalline honeycomb-like structures, forming extended π-delocalization, tunable energy levels, high surface area, regular open channels, and chemical stabilities. The COF-based photocathode showed a photocurrent density of 0.045 mA/cm^2^ at 0.2 V vs RHE with the average H_2_ evolution rate of 14.2 μmol h^–1^ (Figure 9d).^134^

**Figure 10:**
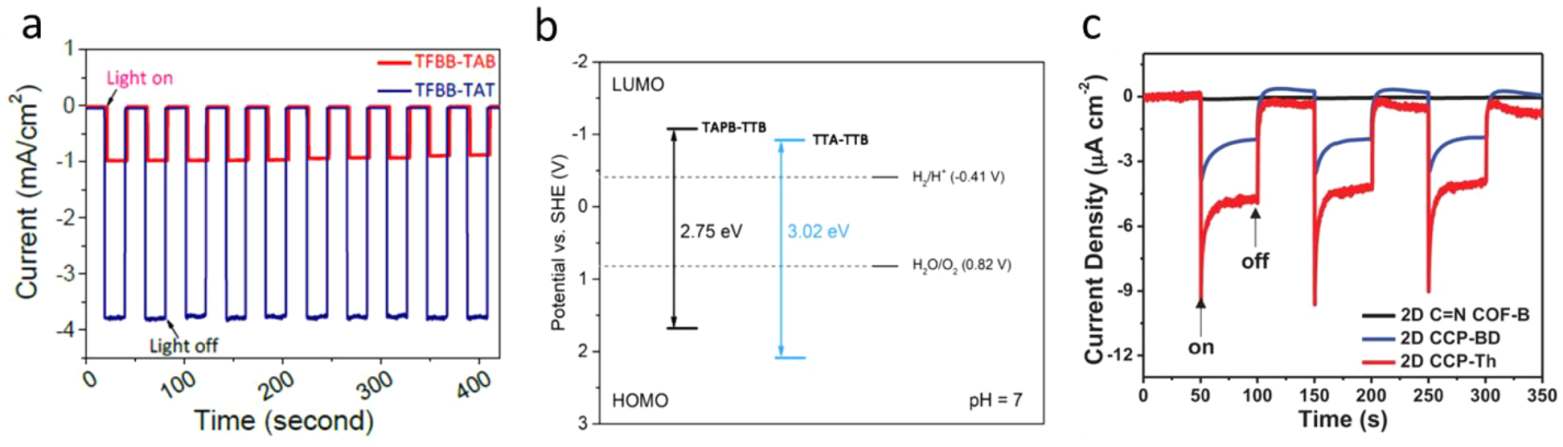
(a) Transient photocurrent responses of TFBB-TAB and TFBB-TAT under dark and light. Adapted with permission from ref (135). Copyright 2020 Zenodo. (b) Hybrid DFT calculated potentials of frontier orbitals and electronic bandgaps in model TAPB-TTB COF and TTA-TTB COF. Adapted with permission from ref (136). Copyright 2021 Wiley-VCH. (c) Photocurrent–time plots for 2D CCP-Th (red line), 2D CCP-BD (blue line), and 2D C=N COF-B (black line) at 0.3 V versus RHE. On: illumination on; off: illumination off. Adapted with permission from ref (137). Copyright 2021 Wiley-VCH.

2D COF TFBB-TAT (triazine-based) and TFBB-TAB (without triazine) were prepared under solvothermal conditions using a Schiff base type condensation between 1,3,5-tris (4-formylbiphenyl) benzene (TFBB), 2,4,6-tris(4-aminophenyl)-1,3,5-triazine (TAT), and 2,4,6-tris(4-aminophenyl)-benzene (TAB), and their PEC performances were studied. The triazine-based (ITO/PEDOT:PSS/TFBB-TAT) COF photocathode showed a water reduction photocurrent density of 4.32 mA/cm^2^ at 0 V vs RHE (Figure 10a),^135^ which was higher than the nontriazine-based TFBB-TAB COF. In addition to the excellent photoabsorption property, TFBB-TAT showed enhanced charge transfer. Similarly, Dai et al. prepared two 2D COFs (TTA-TTB and TAPB-TTB) based on 2,4,6-triphenyl-1,3,5-triazine by introducing an electron donor triphenylbenzene to reduce the optical bandgap for extended visible light absorption and improved charge transfer. The FTO/TAPB-TTB photocathode showed a photocurrent density of 0.110 mA/cm^2^ at 0 V vs RHE, at pH 7.0, in the absence of any sacrificial agents, which was higher than the FTO/TTA-TTB (0.035 mA/cm^2^).^136^ The extended light absorption and promoted charge transfer were found to be crucial to enhance the PEC water reduction, in accordance with the first-principles calculations. Figure 10b shows the electronic bandgaps of the TAPB-TTB and TTA-TTB COFs modeled using DFT, consistent with their PEC performance. Another COF-based photocathode composed of a bithiophene-bridged donor–acceptor-based 2D sp^2^ carbon linkages was synthesized using Knoevenagel polymerization between 2,3,8,9,14,15-hexa(4-formylphenyl) diquinoxalino[2,3-a:2′,3′-c]phenazine (HATN-6CHO), an electron-accepting building block, and the first electron-donating linker 2,2′-([2,2′-bithiophene]-5,5′-diyl)diacetonitrile (ThDAN).^137^ This bithiophene-based COF (2D CCP-Th) photocathode displayed a water reduction photocurrent density of 0.0079 mA/cm^2^ at 0 V vs RHE (Figure 10c), which was higher than the COF prepared using biphenyl-bridged COF (2D CCP-BD).

Besides COF, other known coordinated polymers are metal–organic framework (MOF) materials, which offer the opportunities to develop highly ordered three-dimensional (3D) structures for PEC production of fuels. The pore size control alongside the tunable framework structure offers the appropriate active sites with long-time stability. By choosing suitable central metal atoms, it is possible to create photoresponsive MOF materials. Moreover, the large surface areas and selective porosities in MOF materials can be potentially applied in gas–solid-phase reactions, such as CO_2_ reduction reactions. Thus, the combination of MOFs with appropriate semiconductors can be a promising approach to design effective solar conversion processes. The MOF-based photocatalyst suspension has been widely reported and reviewed,^138^ though photocathodes made from MOFs are much less discussed.

**Figure 11:**
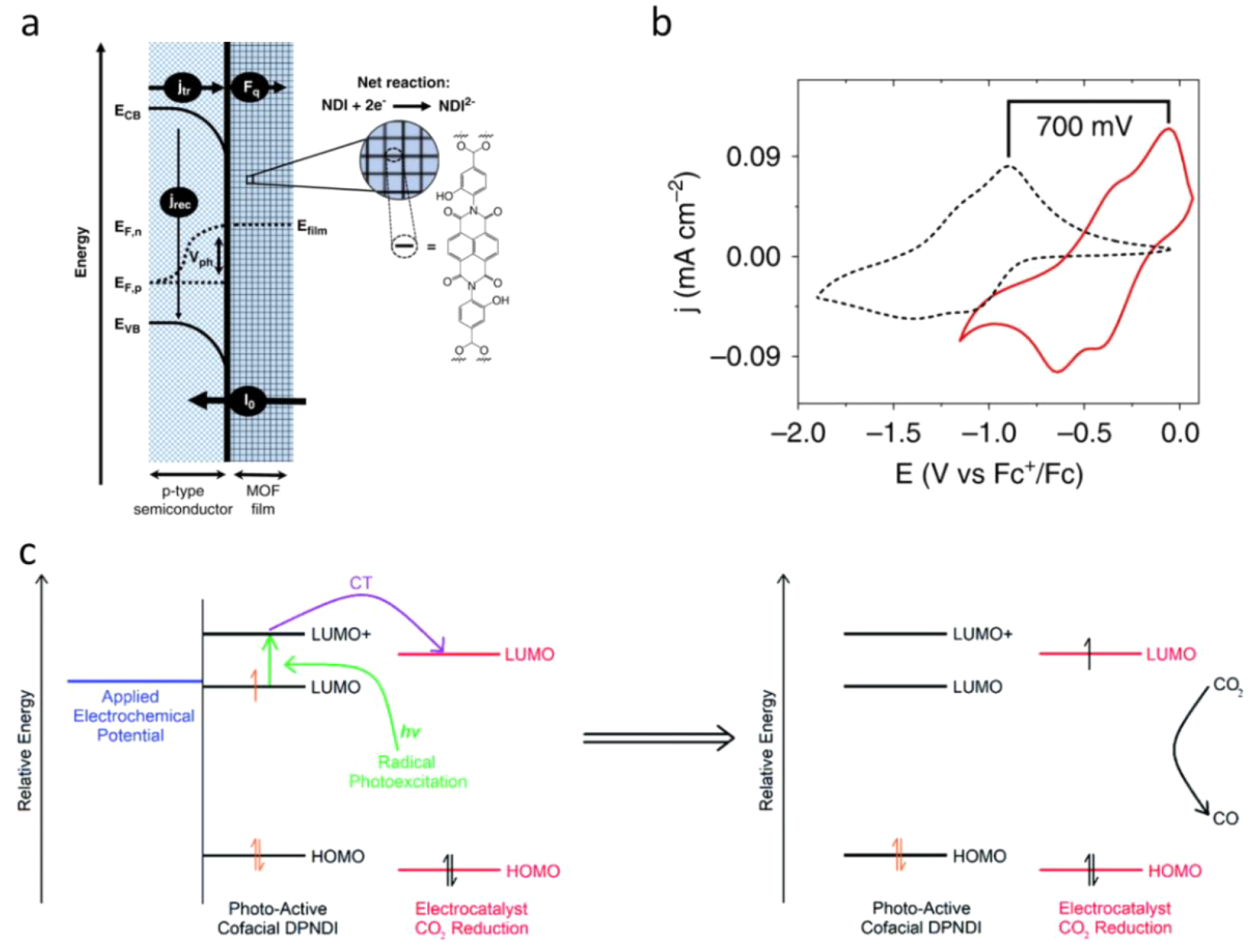
(a) Schematic representation of the MOF surface coating on p-Si. *E*_VB_ and *E*_CB_ are the energetic positions of the valence and conduction band, respectively, *E*_F,p_, and *E*_F,n_ are the quasi-Fermi levels of the holes and electrons, respectively, *V*_ph_ is the semiconductor photovoltage, and *E*_film_ is the electrochemical potential of the MOF film. The molecular structure of the linker is pictured on the right. (b) Cyclic voltammograms of Zr(NDI)@FTO (black dashed) and an illuminated Zr(NDI)|TiO_2_@GaP working electrode (red) at a scan rate of 100 mV s^–1^ with 0.5 M LiClO_4_ in DMF as the supporting electrolyte. The red solid data were collected under AM 1.5 illumination. Adapted with permission from ref (139). Copyright 2020 Nature Springer. (c) Schematic showing the photocathode role of csiMOF-6 in a CO_2_ photoelectrochemical reduction system employing a rhenium electrocatalyst. A green arrow indicates photoexcitation, the purple arrow indicates CT, and the black arrow signifies CO_2_ reduction to CO. Adapted with permission from ref (140). Copyright 2021 Royal Society of Chemistry.

The surface coating of MOF material onto a semiconductor would increase the interfacial charge transfer. The porous structure of MOF either ensures high optical absorption or can act as a protective layer to prevent the leaching and aggregation of the photosensitizing particles. For instance, a redox-active MOF was coated on a p-type Si (Figure 11a), which showed a photovoltage of 0.3 V, whereas p-Si functionalized with a naphthalene diimide derivative monolayer exhibited no photoresponse.^139^ Furthermore, the same MOF coating on a GaP semiconductor shifted the photovoltage to +0.7 V (Figure 11b), which is the highest reported for GaP in a PEC application. This emphasizes the advantage of MOF films regarding enhanced photocathodic operation. In addition, MOF film directly grown on the surface of a substrate improved the diffusion of charges through the film, thereby increasing the photocathodic current.^139^

Cardoso et al. have reported the MOF-based TiO_2_ nanotubes photocathode for CO_2_ reduction, in which the zeolite imidazole framework-8 (ZIF-8) nanoparticles were deposited on TiO_2_ nanotubes using a layer-by-layer process.^141^ The prepared polymer photocathode (Ti/TiO_2_NT-ZIF-8) reduced CO_2_ to ethanol (up to 10 mM) and methanol (0.7 mM) in a 0.1 M Na_2_SO_4_ electrolyte at 0.1 V under UV–vis irradiation for 3 h at room temperature. The utilization of ZIF-8 has the dual advantage that it acted as an active site for CO_2_ adsorption and activation and also functioned as a cocatalyst to facilitate electron transfer. In a report, Hou et al. used a double solvents approach to synthesize Pt@NH_2_-MIL-125(Ti) MOF and fabricated the photocathode by drop-casting on the ITO substrate to study the PEC H_2_ production.^142^ The optical characterization showed very good absorption in the visible region, precisely at 500 nm. The H_2_ evolution photocurrent onset was observed at 0.3 V for this MOF-based photocathode. Though the PEC water reduction activity was small, it demonstrated the applicability of the MOFs as photocathode materials for solar fuel conversion. Similarly, a new cofacial photo- and redox-active MOF, i.e., *N*,*N*′-di(4-pyridyl)-1,4,5,8-naphthalenediimide (DPNDI), denoted as csi-MOF-6, was synthesized by Ding et al. and used to fabricate a photocathode by blending it with a photosensitizer into a [Re(bipy-tBu)(CO)_3_Cl] electrocatalyst (Figure 11c) for PEC CO_2_ reduction.^140^ The reaction was performed with a small overpotential under broad visible light irradiation in a CO_2_-saturated 0.1 M [nBu_4_N]PF_6_/MeCN electrolyte, and CO was produced with a FE of 78% and a TON of 7.

### 3.3 Poly(3-hexylthiophene)-P3HT-Based Photocathodes

P3HT is a commercially available π-conjugated polymer with sufficient solubility in chlorinated organic solvents and has a direct low bandgap of 1.9 eV. The charge carrier mobility is high in P3HT as it has a high degree of intermolecular order. The LUMO of the P3HT is more negative than the water and CO_2_ reduction potentials; hence, it can be used as a direct photocathode material for solar fuel synthesis. A solution-processed regioregular P3HT photocathode was reported for PEC water reduction, which showed a photocurrent density of 0.020 mA/cm^2^ under one sun irradiation. In addition, a good stability was observed over a few hours of irradiation.^143^ The mechanism for proton reduction will be discussed in Section 5. Briefly, the P3HT surface adsorbed H species at the α-site of the thiophene ring. The protonated polymer–electrolyte interface received the photogenerated electrons and produced H_2_ followed by regeneration of the polymer surface. To enhance the binding of the polymers to the substrate, direct polymerization from the respective monomers has been recommended.

**Figure 12:**
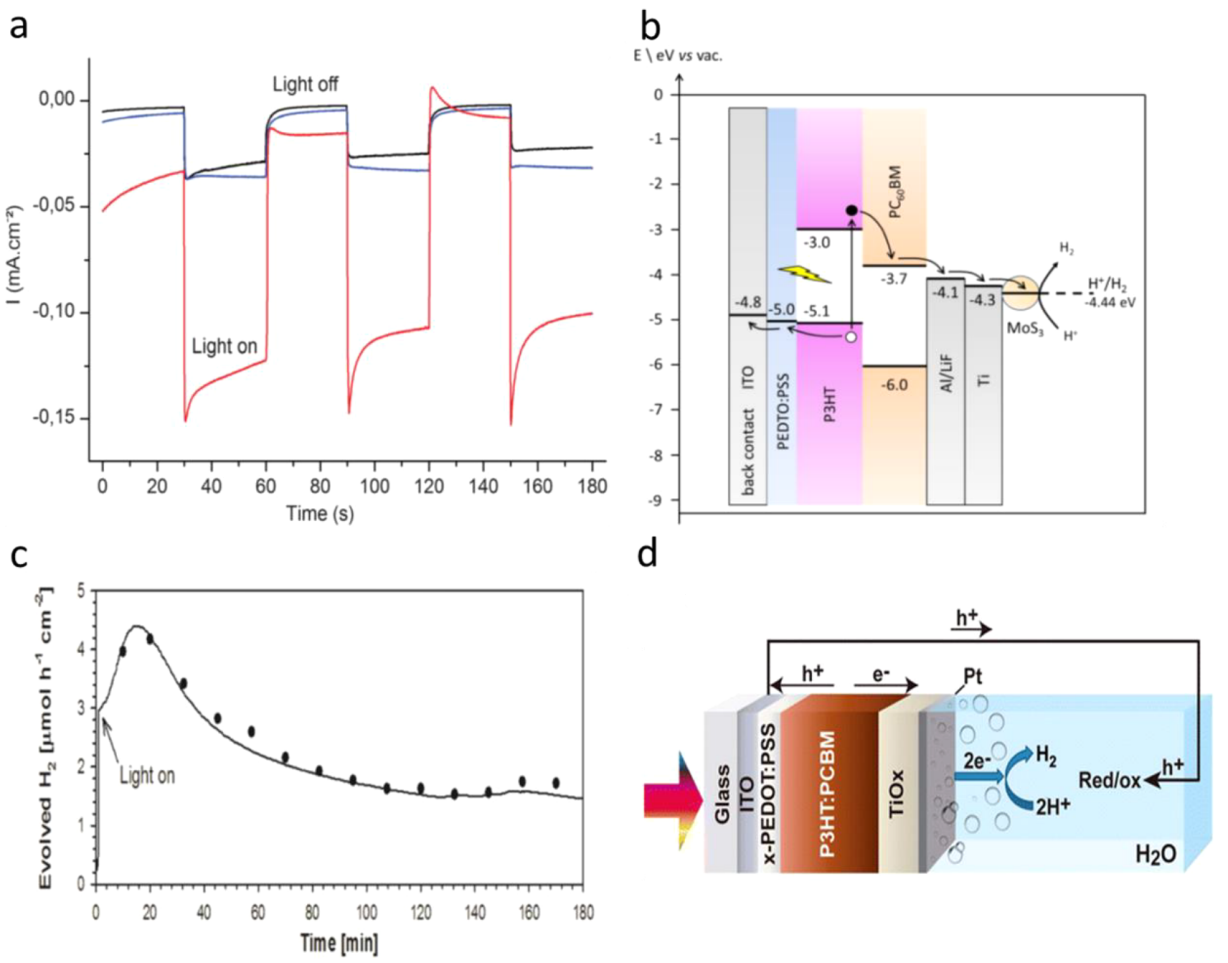
(a) Electrolysis at a bias potential of +0.16 V vs RHE, with chopped visible light, in H_2_SO_4_ (0.5 M). Photocathode: black, P3HT:PCBM; blue, MoS_3_/P3HT:PCBM; red, TiO_2_:MoS_3_/P3HT:PCBM photocathode. Electrode area: 0.5 cm^2^. Adapted with permission from ref (144). Copyright 2013 Royal Society of Chemistry. (b) Energy level diagram of the device in contact with the electrolyte. Electrons and holes are represented by black and white dots, respectively. Adapted from ref (145). Copyright 2015 American Chemical Society. (c) Hydrogen evolution of the OPEC measured under continuous 1 sun irradiation at 0 V versus RHE registered experimentally (square points) and theoretically calculated from the measured current by Faraday’s law. (d) Device architecture of the optimized organic photoelectrochemical cell (OPEC), showing the electronic process during device operation. Adapted from ref (146). Copyright 2015 American Chemical Society.

Though P3HT showed direct PEC performance, the charge carrier recombination was presumably a key issue and hence BHJ formation has been introduced to enhance the charge transfer, analogous to the evolution of organic photovoltaics. BHJs are typically obtained by blending a p-type P3HT polymer with an electron acceptor layer. For instance, fullerene derivatives were used as electron acceptors. The BHJ photocathode made up of P3HT:fullerene absorbed light and separated the photogenerated electrons from holes very efficiently. For example, the BHJ photocathode was fabricated by using a P3HT:phenyl-C61-butyric acid (PCBM) blend and sandwiched between the molybdenum sulfide (MoS_3_) electron selective layer and the PEDOT:PSS hole selective layer. Next, a TiO_2_ thin film was spin-coated on MoS_3_ to protect P3HT:PCBM from oxygen and water exposure. The PEC characterization of the BHJ photocathode exhibited a high photocurrent of 0.100 mA/cm^2^ at 0.16 V vs RHE (Figure 12a).^144^ After two years, the same group attempted to increase the electron extraction to the catalyst layer MoS_3_ by adding the metal Al, Ti, and nanocarbon (C_60_) as an interfacial layer. The ITO/PEDOT:PSS/P3HT:PCBM/LiF/Al/Ti-MoS_3_ and ITO/PEDOT:PSS/P3HT:PCBM/C_60_-MoS_3_ photocathodes showed dramatically enhanced photocurrent densities of 8 mA/cm^2^ and 1 mA/cm^2^, respectively, at 0.6 V vs RHE.^145^ The proposed electron extraction mechanism was shown in Figure 12b.

Similarly, Haro et al. fabricated an ITO/PEDOT:PSS/P3HT:PCBM/TiO_*x*_-Pt photocathode and obtained a photocurrent density of 1 mA cm^–2^ at −0.1 V vs RHE and stable H_2_ evolution of 1.5 μmol h^–1^ cm^–2^,^146^ as shown in Figure 12c. The experimental results agreed with the theoretical value of H_2_ production, which is represented in the dashed line, indicating nearly 100% FE. The schematic of the photocathode and the mechanism of H_2_ production are represented in Figure 12d. Fumagalli et al. reported a photocathode made up of P3HT:PCBM sandwiched between the hole separation layer MoO_3_ and electron separation layer TiO_2_. The Pt cocatalyst loaded on the TiO_2_ photocathode (FTO/MoO_3_/BHJ/TiO_2_/Pt) showed the photocurrent response of 3 mA cm^–2^ at 0 V vs RHE with the 100% FE for H_2_ evolution.^147^

Rojas et al. used the cuprous iodide (CuI) as a hole selective layer to effectively separate the holes with high performance and stability, along with an electron selective TiO_2_ layer to fabricate the inorganic–organic hybrid BHJ photocathode.^148^ After adding a thin Pt catalyst layer, the FTO/CuI/P3HT: PCBM/TiO_2_-Pt photocathode produced the high photocurrent density of 8 mA at 0 V vs RHE under one sun condition in the pH 1.0 electrolyte. The IPCE value of 50% was obtained with a FE of 100% using the above photocathode. To improve the stability of the photocathode, the polyethylenimine (PEI) layer, due to its good adhesion, hydrophilicity, and proton affinity, was coated on the surface. Such a layer was expected to minimize the Pt loss, and the chronoamperometry results revealed that the photocurrent decrease over time was 2-fold reduced after PEI coating, suggesting improved stability.

Table 2 summarizes representative polymer-based photocathodes reported for water reduction and CO_2_ conversion reactions. These photocathodes performances were evaluated in terms of photocurrent density or TON under different working conditions. Most of the photocathodes had their PEC reduction activity reported at the aqueous interface. The long-term stability parameters were not discussed in most of the reported polymer-based photocathodes, which should be paid particular attention as organic substances tend to be less stable under strong light irradiation compared with their inorganic counterparts.

**Table 2:** Representative Polymer-Based Photocathodes and Their PEC Performance

photocathode design	cathodic photocurrent (mA/cm^2^ vs RHE) or TON	PEC reaction	pH	ref
CN_*x*_				
FTO/B-C_3_N_4_/Rh	0.01 at –0.2 V	CO_2_ reduction	7.3	(121)
ITO/P-C_3_N_4_	1.5% IPCE at 420 nm	–	7.0	(102)
FTO/def-C_3_N_4_	0.010 at 0 V	–	7.0	(122)
FTO/Pt@g-C_3_N_4_/GDY	0.133 at 0 V	water splitting	7.0	(123)
FTO/NiO-C_3_N_4_	0.070 at 0.42 V	water splitting	6.5	(124)
FTO/NiO-C_3_N_4_	0.020 at 0 V	water splitting	6.5	(126)
ITO/ZnO-Au-C_3_N_4_/Pt	0.290 at 0 V	water splitting	6.8	(127)
FTO/Cu-C_3_N_4_	0.200 at 0.42 V	water splitting	6.8	(128)
COF and MOF				
FTO/BDT-ETTA COF/Pt	0.129 at 0.1 V	water splitting	10.0	(133)
FTO/g-C_18_N_3_-COF/Pt	0.045 at 0.2 V	water splitting	6.8/3.0	(134)
FTO/TAPB-TTB	0.110 at 0 V	water splitting	7.0	(136)
FTO/Si@TiO_2_-Zr(NDI)	+0.3 V (photovoltage)	–	6.0	(139)
FTO/GaP@TiO_2_-Zr(NDI)	+0.7 V (photovoltage)	–	6.0	(139)
FTO/csiMOF-6[Re(bipy-tBu)(CO)_3_Cl]	78% FE, TON 5	CO_2_ reduction	organic	(140)
P3HT				
FTO/P3HT	0.020 under one sun	–	0.1	(143)
ITO/PEDOT:PSS/P3HT:PCBM/MoS3-TiO2	0.100 at 0.16 V	–	0.1	(144)
ITO/PEDOT:PSS/P3HT:PCBM/C_60_-MoS_3_	1 at 0.6 V	–	0.1	(145)
ITO/PEDOT:PSS/P3HT:PCBM/TiOx-Pt	1 at –0.1 V	water splitting	2.0	(146)
FTO/MoO_3_/BHJ/TiO_2_/Pt	3 at 0 V	water splitting	1.37	(147)
FTO/CuI/P3HT: PCBM/TiO_2_-Pt	8 at 0 V	water splitting	1.0	(148)
others				
FTO/PTEB	0.010 at 0 V	water splitting	6.8	(154)
FTO/PTEB_1.3_-*co*-PDET_1_	0.021 at 0 V	water splitting	6.8	(154)
FTO/pDEB/p(DEB_0.9_-*co*-TEB_0.1_)	0.055 at 0.3 V	water splitting	6.8	(155)

### 3.4 Other Polymer Photocathodes

A direct polymer-based photocathode, for instance, a polyacetylene-based photocathode, was reported for visible-light-driven water reduction. It showed a cathodic photocurrent of 0.005 mA/cm^2^ at −0.46 V vs RHE at pH 5.7.^62^ Subsequently, several polymers such as polypyrrole (PPy),^149^ polyaniline,^150^ and poly(p-phenylene)^151^ have been reported for the direct PEC H_2_ production on the surface of the polymer photoelectrodes, even though poor charge separation efficiency in the absence of a selective charge extraction layer is a major challenge. In addition, stability has been a crucial issue to use the polymers directly in contact with electrolytes. Later, the conjugated acetylenic polymers have been proposed as they have strong electrostatic interaction with water molecules due to the presence of an electron-rich C-to-C triple bond. For instance, the conjugated polymers such as poly(1,3,5-triethynylbenzene) (PTEB) and poly(1,3,5-tris(4-ethynylphenyl)benzene) (PTEPB) nanoflakes were reported for the photocatalytic activity toward H_2_ and O_2_ evolutions.^152^ The PTEB electrode without doping may act as an n-type semiconductor, however, nitrogen doping can convert it into a p-type semiconductor.^153^ PTEB polymerized on the surface of the Cu or Ti was reported for PEC H_2_ evolution with the photocurrent density of 0.010 mA/cm^2^ at 0 V vs RHE. With the use of this photocathode, an H_2_ evolution rate of 0.253 μmol h^–1^ cm^–1^ was achieved with a FE of >90%.^154^ An increased photocurrent density of 0.021 mA/cm^2^ was observed upon the incorporation of 2,5-thieno[3,2-*b*]thiophene into the polymer structure (p(TEB1.3-*co*-DET1)). Later, the same group prepared a poly(1,4-diethynylbenzene) (pDEB) conjugated polymer by controlled copolymerization of 1,4-diethynylbenzene (DEB) and 1,3,5-triethynylbenzene (TEB) then studied PEC water reduction activity.^155^ The water reduction photocurrent density of the homojunction FTO/pDEB photocathode was 0.019 mA/cm^2^, which was enhanced to 0.055 mA/cm^2^ for the gradient-homojunction pDEB/p(DEB_0.9_-*co*-TEB_0.1_) photocathode at 0.3 V versus RHE at pH 7.0.

The conducting polymer PPy was sometimes used as a supporting substrate to deposit the photoactive complex to fabricate the effective PEC photocathode for solar fuel production. The PPy allows the metal complex to deposit efficiently without modifying the intrinsic properties. For instance, Lattach et al. used PPy to deposit the Ru(II) complex using anodic polymerization onto a carbon electrode,^156^ which was then incorporated with MoS_*x*_ by an ion-exchange method followed by electrochemical reduction of MoS_4_^2–^. The PPy-Ru/MoS_*x*_ photocathode showed an H_2_ evolution rate of 0.53 μmol cm^–2^. PPy has also been used as an electrocatalyst for CO_2_ reduction reactions by replacing the transition metal electrocatalysts to reduce the energy barrier at the semiconductor-electrolyte junction. Won et al. decorated a p-ZnTe photocathode with PPy to reduce CO_2_ into formic acid and carbon monoxide (CO).^149^ Beyond this, organic polymer-based dyes have also been used to improve the performance of the photocathode. Simply coating organic dye molecules via chemisorption or covalently linking organic molecules such as perylene derivatives and donor–acceptor dyes, the visible region of the solar spectrum could be efficiently absorbed. Numerous reports can be found on polymeric dye-sensitized photocathodes,^157−160^ which is not the focus of this review.

Overall many polymer photocathodes were reported, while the photocurrent is still quite moderate. Except the P3HT-based polymer, which showed a maximum photocurrent density of 8 mA/cm^2^ at 0 V vs RHE, the highest cathodic photocurrent was 0.29 mA/cm^2^ observed on the solid Z-scheme ITO/ZnO-Au-C_3_N_4_/Pt photocathode, which is nearly 1 order of magnitude lower than that achieved on the benchmark inorganic photocathodes. Therefore, much effort is still required to improve the photocurrent of polymer photocathodes.

### 3.5 Bias-Free Photoanode|Photocathode System

In addition to the photoanode|dark cathode and photocathode|dark anode systems, a bias-free tandem cell PEC approach has also been recently used for solar fuel production. The term “tandem-cell approach” has been used to represent two types of configurations, (i) a photoanode or photocathode coupled with photovoltaics (PEC|PV) and (ii) a self-powered photoanode|photocathode system. Several PEC|PV systems were reported for solar H_2_ production^44,161^ and CO_2_ reduction,^162−164^ which is beyond the scope of the present review and the readers can find the related reviews in the literature.^4,44,165^ The past few years have witnessed the photoanode|photocathode tandem cell approach (Figure 1d) for solar fuel production without the need of any external bias, which potentially reduces the overall cost of the system and increases its efficiency. In this configuration, a n-type semiconductor coated electrode is used as a photoanode for water oxidation to produce O_2_ and protons and a p-type semiconductor-loaded electrode used as a photocathode for proton reduction to produce H_2_ or CO_2_ reduction to produce methanol or high-value chemicals. While both photoelectrodes are connected in series, the light can be harvested by the photoanode and photocathode or two light source can be used. It is also feasible to have both electrodes separated by a membrane to separate the reaction products. So far, the majority of the reported photoanode|photocathode tandem PEC device is made up of inorganic semiconductors^166−169^ and, hence, limited us to explore further in this review. To the best of our knowledge, the direct use of polymer-based semiconductors to fabricate a photoactive anode and a photoactive cathode in a tandem cell approach is yet to come. But, the organic dye-sensitized photoanode|dye-sensitized photocathode tandem PEC approach has been reported,^170,171^ in which inorganic metal oxide semiconductors such as TiO_2_ and NiO were used to extract the charges from the photoexcited dyes followed by the respective oxidation and reduction reactions performed.

## 4 Molecular and Electro-Cocatalysts

Robust and efficient cocatalysts can be introduced into a conjugated polymer photoelectrode not only to improve the catalytic activity but also to enhance the stability of the polymer. As mentioned above, there were limited polymer photoelectrodes reported, therefore few cocatalysts were tested on polymer photoelectrodes, while there were many cocatalysts loaded on either dyes or organic scaffolds to evidence their efficiency. Due to the similarity of the chemical bonds or affinity between these organic scaffolds and cocatalysts as well as between potential polymer photoelectrodes and cocatalysts, these efficient cocatalysts are reviewed here. To date, ruthenium (Ru)-, cobalt (Co)-, rhenium (Re)-, and manganese (Mn)-based molecular catalysts and inorganic catalysts, e.g. Pt, RuO_*x*_, IrO_*x*_, NiO_*x*_, CoO_*x*_, have been extensively grafted on polymer photoelectrodes or organic scaffolds to proceed PEC reactions. To incorporate the catalysts into the matrix, *in situ* electropolymerization/polymerization, layer-by-Layer (LbL) assembles, etc. have been widely used. Here, the recent advancement of cocatalysts in the matrix, including both polymer photoelectrodes and organic scaffolds is addressed, including for the PEC OER, HER, and CO_2_ reduction reaction (CRR).

### 4.1 Molecular OER Cocatalysts

#### 4.1.1 Ru-Based Molecular Cocatalysts

Abundant OER catalysts are based on Ru complexes due to the basis of water oxidation at Ru progressing between four oxidation states: Ru^II^, Ru^III^, Ru^IV^, and Ru^V^. With Ru^II^ and Ru^III^, an aqua ligand is commonly presented, forming the active site for catalysis. Activation of Ru^IV^ spurs the complete proton loss from the aqua ligand and the formation of the Ru-oxo bond. Water oxidation generally occurs at the highest oxidation state, Ru^V^, and the Ru-oxo complex undergoes nucleophilic addition to another water molecule in solution eventually leading to the evolution of O_2_ from the complex and reformation of the Ru^II^ or Ru^III^ aqua complex.^172−175^ Ru(bda)L_2_ (bda = 2,2′-bipyridine-6,6′-dicarboxylate; L = 4-picoline, isoquinoline, pyridine, imidazole, or thiophene), a typical class of mononuclear Ru complexes, have been widely exploited both under electrochemical and PEC conditions.^176,177^ Inspired by the stabilization function of phenolate and carboxylate ligands for high valence states of Mn in natural photosynthesis photosystem II, carboxylate ligands were thereby incorporated into Ru molecular OER catalysts to access high valence Ru = O species at a low oxidation potential,^178^ which were then utilized in the water oxidation reaction.^172,176,177^ For instance, Ru(bda)L_2_ (**OER1**; L = 4-picoline) was reported to have an onset potential of ca. 0.98 V vs normal hydrogen electrode (NHE) at neutral pH in an electrochemical water oxidation reaction.^172^ Such low overpotential (0.98 V) renders a homogeneous water oxidation reaction by combing with the photosensitizer [Ru(bpy)_3_]^3+^.^177^ Due to the impressive water oxidation properties in the homogeneous system, developing strategies to further assemble the molecular catalyst in the heterogeneous platform without activity loss attracts much attention. This remains a major challenge holding back molecular artificial photosynthesis.^179^

**Figure 13:**
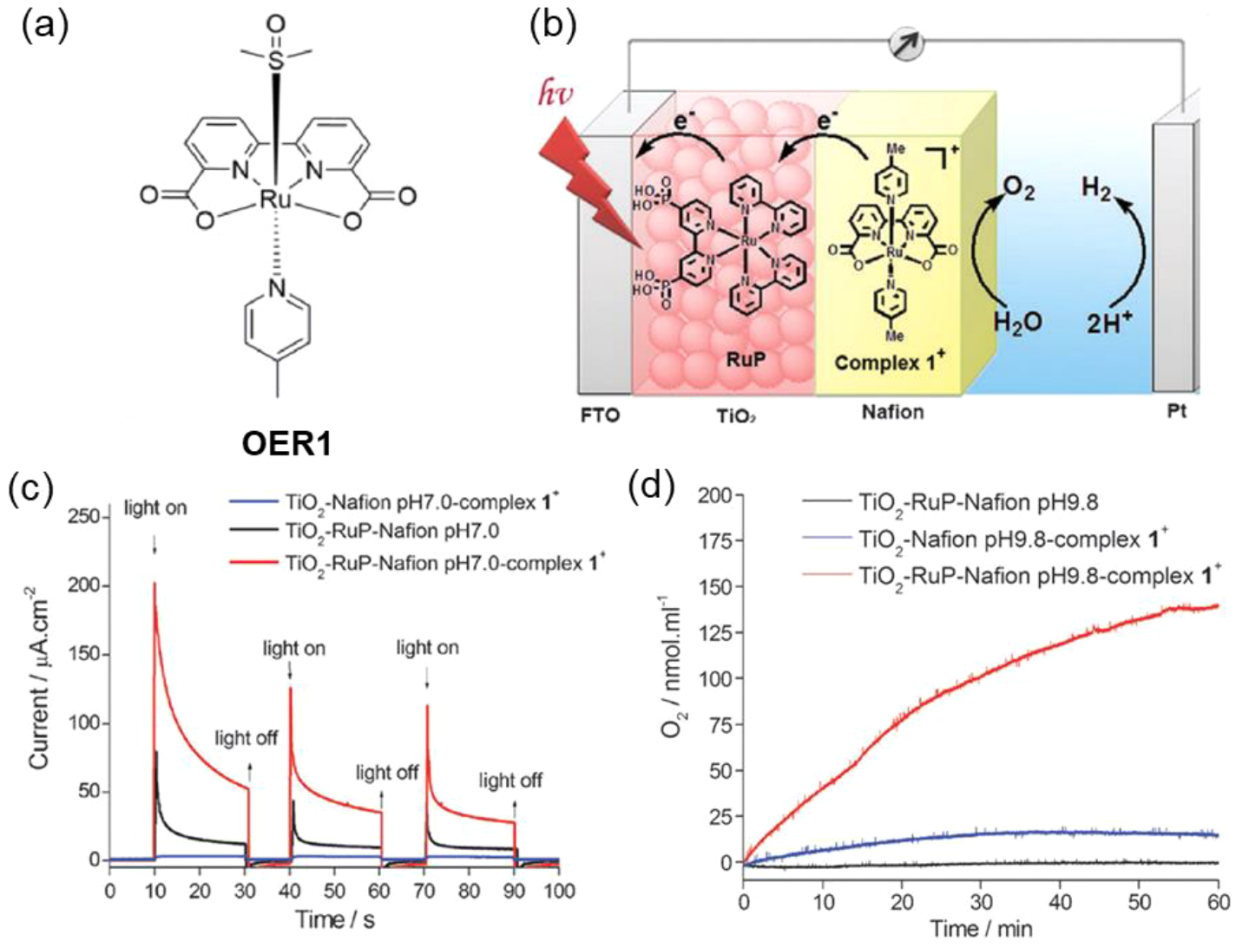
(a) Chemical structure of OER1. (b) Light-driven PEC water splitting device, consisting of TiO_2_-RuP/Nafion-OER1, a Pt cathode, and an aqueous electrolyte. (c) Transient short-circuit current responses to on–off cycles of illumination. The illumination is provided by a light-emitting diode operated in a 0.1 M Na_2_SO_4_ aqueous solution in PEC devices without applying any bias. Nafion pH 7.0 represents the related Nafion film prepared using a pH 7.0 Nafion solution, respectively. (d) Oxygen evolution in PEC devices without applying any bias, operated in a pH 7.0 phosphate buffer solution, detected by a Clark electrode, and illuminated by a 500 W xenon lamp through a 400 nm cut-off filter. Complex 1^+^ indicates the OER1. Adapted with permission from ref (180). Copyright 2010 Royal Society of Chemistry.

To address this issue, OER1 embedded in a Nafion polymer was covered on the dye [Ru(bpy)_2_(4,4′-(PO_3_H_2_)_2_bpy)]^2+^ (RuP) sensitized nanostructured TiO_2_ photoanode (Figure 13, labeled as TiO_2_-RuP/Nafion-OER1).^180^ First, the Nafion polymer has good electrical conducting properties and high chemical/thermal stability, benefiting photoelectrochemical application, due to the existence of numerous sulfonic acid groups.^181^ Moreover, the oxidation potential *E*_1/2_(Ru^III^/^II^) of RuP is more positive than the onset potential of OER1 for water oxidation at pH 7.0, thus thermodynamically allowing the photogenerated Ru^III^ in RuP to drive OER1. A negligible photocurrent or fast decay was recorded for the photoanodes without photosensitizer RuP or catalyst OER1 modification. Whereas the TiO_2_-RuP/Nafion-OER1 showed dramatically enhanced photocurrent very likely due to efficient electron transfer from the catalyst to the photooxidized dye. The photocurrent decay rate of TiO_2_-RuP/Nafion-OER1 was strongly related to the initial pH of the Nafion membrane, probably due to the rapid proton release during water oxidation thereby affecting the catalytic properties of OER1.^177^ No O_2_ could be produced without light illumination for all photoanodes. For TiO_2_/Nafion-OER1, only 16 nmol mL^–1^ O_2_ was generated after 60 min illumination. In the presence of RuP but without OER1 (TiO_2_-RuP/Nafion), no O_2_ could be produced as well. In contrast, in the coexistence of RuP and OER1 (TiO_2_-RuP/Nafion-OER1), 140 nmol mL^–1^ O_2_ was obtained after 60 min irradiation. These results clearly proved the light-driven water oxidation by this complete catalytic assembly. Accordingly, assuming all OER1 participating in the water oxidation reaction, TON of 16 was obtained with a corresponding turnover frequency (TOF) of 27 h^–1^.

**Figure 14:**
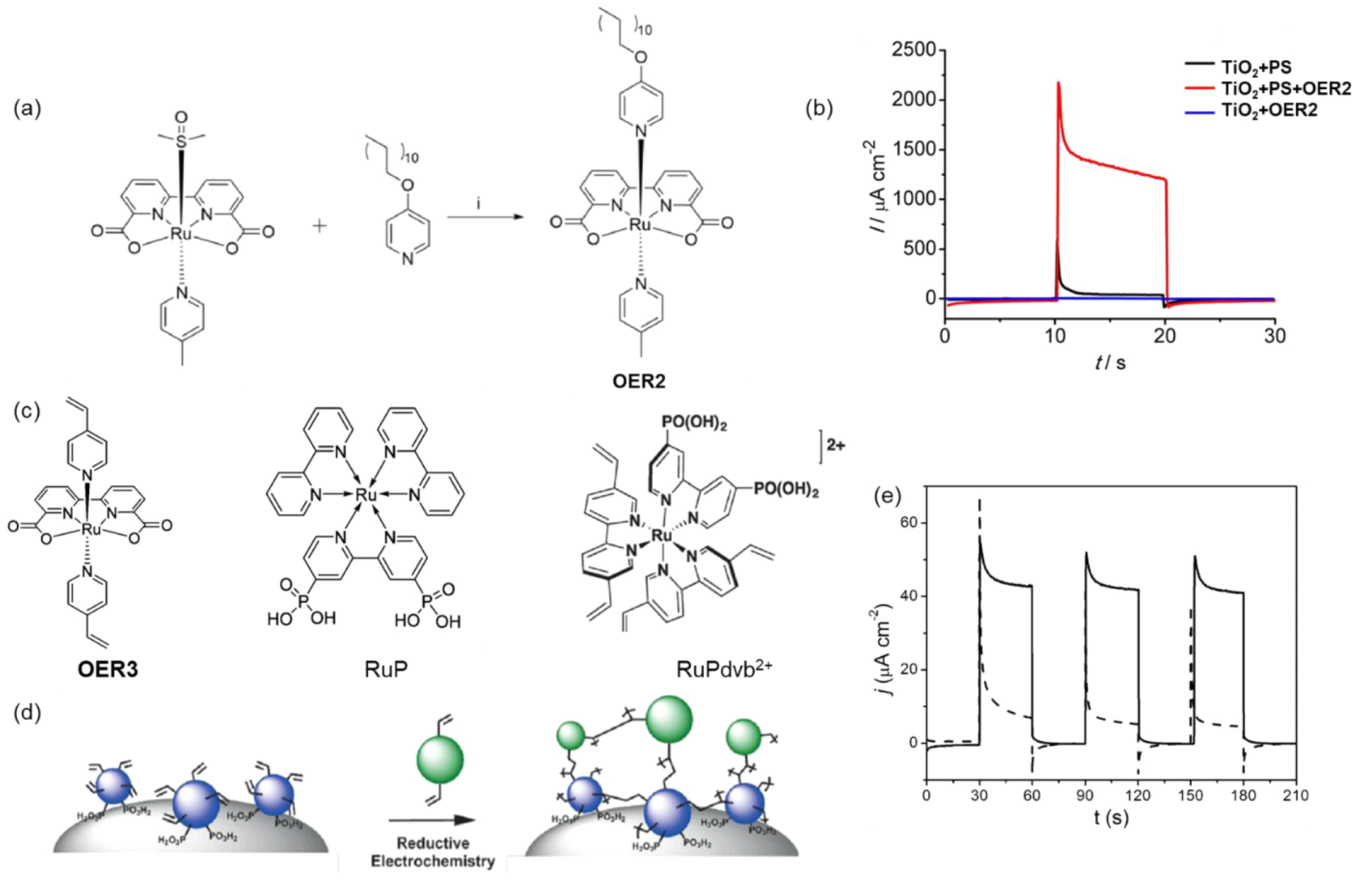
(a) Chemical structure and synthesis route of the OER2. (b) Photocurrent of three photoanodes (TiO_2_+PS, TiO_2_+PS+OER2, and TiO_2_+OER2) with a 0.2 V vs NHE external bias in pH 6.8 phosphate buffer solution upon visible light irradiation (λ > 400 nm, 300 mW cm^–2^). Adapted with permission from ref (184). Copyright 2015 Wiley-VCH. (c) Chemical structures of OER3, RuP, and RuPdvb^2+^. (d) Schematic diagram of the surface structure following reductive electropolymerization of OER3 on TiO_2_-RuPdvb^2+^. (e) Photocurrents of nTiO_2_-RuPdvb^2+^ (dashed) and nTiO_2_-RuPdvb^2+^-polyOER3 (solid) at a bias of 0.2 V versus SCE. Adapted with permission from ref (186). Copyright 2015 Wiley-VCH.

PMMA (poly(methyl methacrylate)) oligomer is another potential support for immobilizing catalysts. Compared to acidic Nafion, PMMA has nearly no influence on the properties of molecular components and displays superior abilities for assembling pH-sensitive devices, thus rendering the utilization in perovskite solar cells as stable hole-transport materials and the immobilization of molecular components for higher stability.^182,183^ After stabilizing a long carbon chain modified Ru(bda)L_2_ (**OER2**; L = 4-picoline) catalyst by PMMA on top of RuP-sensitized TiO_2_ (Figure 14a,b)^184^ and upon visible light (λ > 400 nm) illumination, a stable photocurrent density of 1.1 mA/cm^2^ was obtained at 0.2 V vs NHE in phosphate buffer solution, while nearly no photocurrent was recorded for the only catalyst or photosensitizer-modified TiO_2_. A maximum IPCE of 9.5% was achieved at 450 nm. A lower photocurrent density with faster decay without long-chain modification indicated that the long carbon chains have a positive effect on immobilizing the catalyst by twining around the polymerized PMMA. After coembedding RuP photosensitizer and OER2 in PMMA, the photocurrent density was further improved to 1.50 mA/cm^2^.^185^

Besides polymer encapsulation, the electropolymerization method enables an “on-surface” preparation of assemblies by electrochemically induced C–C bond coupling. This technique exhibits the capacity for polymer film preparation with a vinyl-functionalized complex, Ru(bda)L_2_ (**OER3**; L = 4-vinylpyridine), on a metal oxide photoanode (Figure 14c–e).^186^ The OER3 was prepared by a simple, microwave-assisted, one-pot reaction via self-assembly of three reagents: bda^2–^ (generated *in situ* by reaction of H_2_bda and triethylamine), Ru(DMSO)_4_Cl_2_, and 4-vinylpyridine. Then, poly-OER3 was obtained by electrochemical polymerization on the RuP-sensitized TiO_2_ film at −2.0 V vs Ag/AgNO_3_ for 200 s in an acetonitrile solution containing OER3.^187,188^ A remarkable initial photocurrent density of ∼3.0 mA/cm^2^ was obtained by the poly-OER3+RuP@TiO_2_ photoanode (phosphate buffer solution, 0.2 V vs NHE), while no significant photocurrent was observed for the RuP@TiO_2_ photoanode. A maximum IPCE of 8.9% was observed at 450 nm, and the FE was measured as 82%. However, the poly-OER3+RuP@TiO_2_ photoanode was unstable with time, indicated by the photocurrent density decreasing from 1.4 to 0.3 mA/cm^2^ after 200 s of light illumination, which was due to the decomposition and/or desorption of the photosensitizer. By replacing the RuP@TiO_2_ with an α-Fe_2_O_3_ nanorod array, stable photocurrent density was obtained for a poly-OER3@Fe_2_O_3_ photoanode after long-term illumination, indicating that photosensitizer decomposition could be substantially suppressed by using α-Fe_2_O_3_ in comparison to RuP.

**Figure 15:**
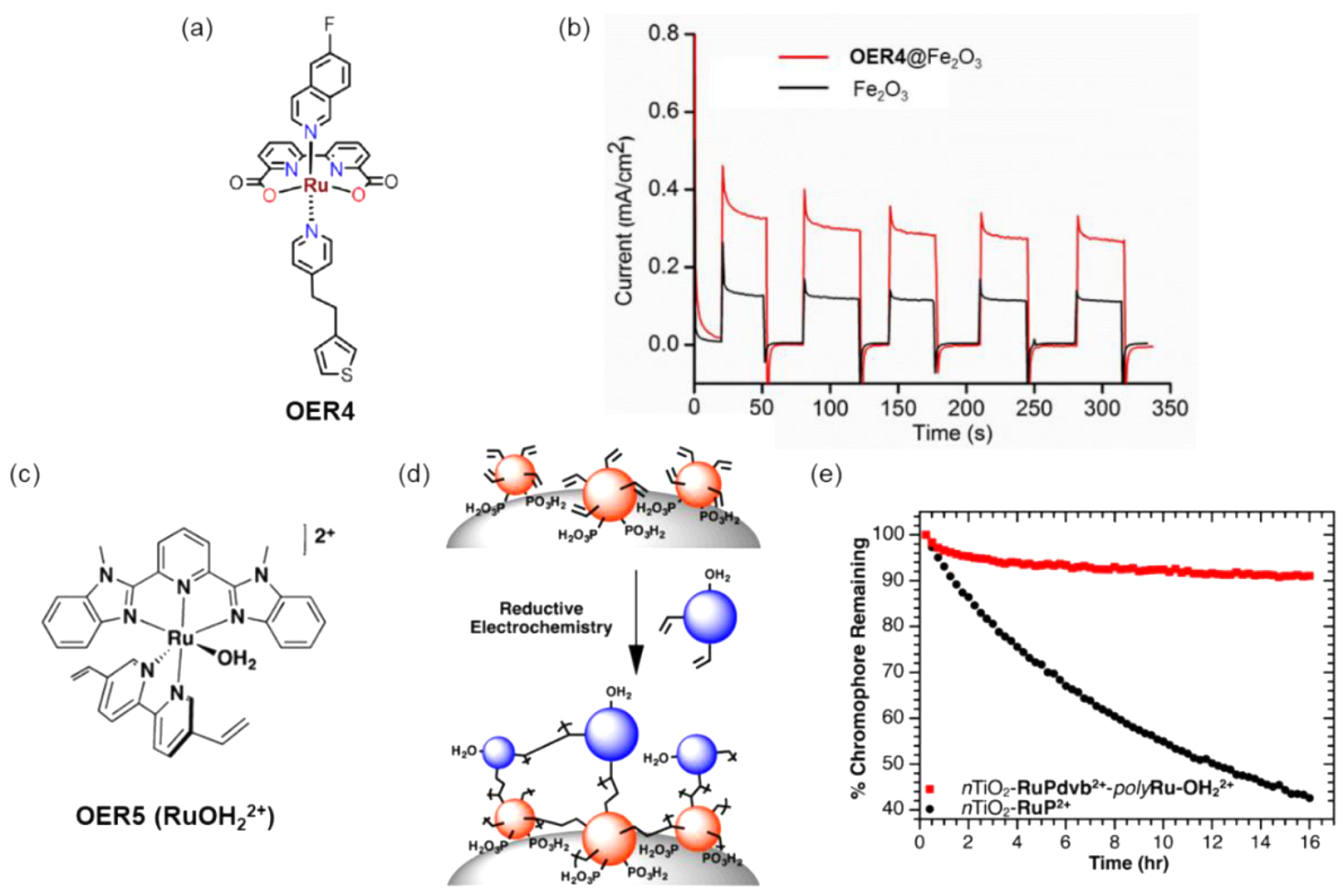
(a) Structure of OER4. (b) Photocurrent densities of the OER4@Fe_2_O_3_ and pristine Fe_2_O_3_ in the phosphate buffer (pH 7) with a bias of 0.8 V vs NHE, and under AM 1.5 G illumination (100 mW cm^–2^). Adapted with permission from ref (189). Copyright 2017 Elsevier. (c) Structures of an OER5 (RuOH_2_^2+^). (d) Schematic diagram of the surface structure following reductive electropolymerization of RuOH_2_^2+^ on nTiO_2_-RuPdvb^2+^. (e) Variation of surface coverage as a function of irradiation time at 475 mW cm^–2^ at 455 nm over a 16 h photolysis period in aqueous 0.1 M HClO_4_. Adapted with permission from ref (190). Copyright 2014 American Chemical Society.

The results above reveal that molecular catalyst poly-OER3 is an efficient and stable catalyst in PEC devices as well as imply the reliability of the electropolymerization method for fabricating efficient and stable catalyst embedded polymer photoelectrode. In addition, replacing the photosensitizer, RuP, by the phosphonate-derivatized light-harvesting chromophore [Ru(dvb)_2_((PO_3_H_2_)_2_bpy)]^2+^ (RuPdvb^2+^; dvb = 5,5′-divinyl-2,2′-bipyridine; (PO_3_H_2_)_2_bpy = [2,2′-bipyridine]-4,4′-diylbis(phosphonic acid)), more stable photocurrents were obtained under white light illumination compared to poly-OER3+RuP@TiO_2_.^186^ The next catalyst **OER4** bears a thiophene unit as appended-bridge ligand and a hydrophobic ligand 6-fluoroisoquinoline instead of 4-iodpyridine (Figure 15a,b).^189^ Under visible light irradiation OER4@Fe_2_O_3_ showed a high and stable photocurrent of over 0.3 mA/cm^2^ at a relatively small bias of 0.8 V vs NHE. This work suggests that the *in situ* polymerization also has the promise to immobilize molecular catalysts on electrode surfaces to build efficient polymer PEC devices.

Vinyl-functionalized [Ru(Mebimpy)-(dvb)(OH_2_)]^2+^ (**OER5 = RuOH**_**2**_^**2+**^; Mebimpy = 2,6-bis(1-methyl-1H-benzo[d]imidazole-2-yl)pyridine, dvb = 5,5′-divinyl-2,2′-bipyridine) is another OER catalyst polymerized with or without the RuPdvb^2+^ chromophore on the surface of the metal oxide film by electropolymerization/electrooligomerization (Figure 15c–e).^190^ In a typical electropolymerization process, propylene carbonate as the solvent rather than CH_3_CN avoids the displacement of the H_2_O ligand for RuOH_2_^2+^. The chromophore/catalyst ratio was controlled by tuning reductive electrochemical cycles. A blue shift in the metal-to-ligand charge transfer absorption maximum from 462 to 453 nm was observed for RuPdvb^2+^ in the electropolymerized films. This shift is consistent with the conversion of the π* acceptor vinyl substituents in RuPdvb^2+^ to saturated, electron-donating alkyl substituents in the electropolymerized polymers, suggesting the formation of C–C bonds between RuPdvb^2+^ and RuOH_2_^2+^ in the surface assembly.^191,192^ The surface coverage of the chromophore in nTiO_2_-RuPdvb^2+^ decreased by ∼70%, while only ∼10% was lost for nTiO_2_-RuPdvb^2+^-polyRuOH_2_^2+^ after sustaining 16 h of irradiation.

**Figure 16:**
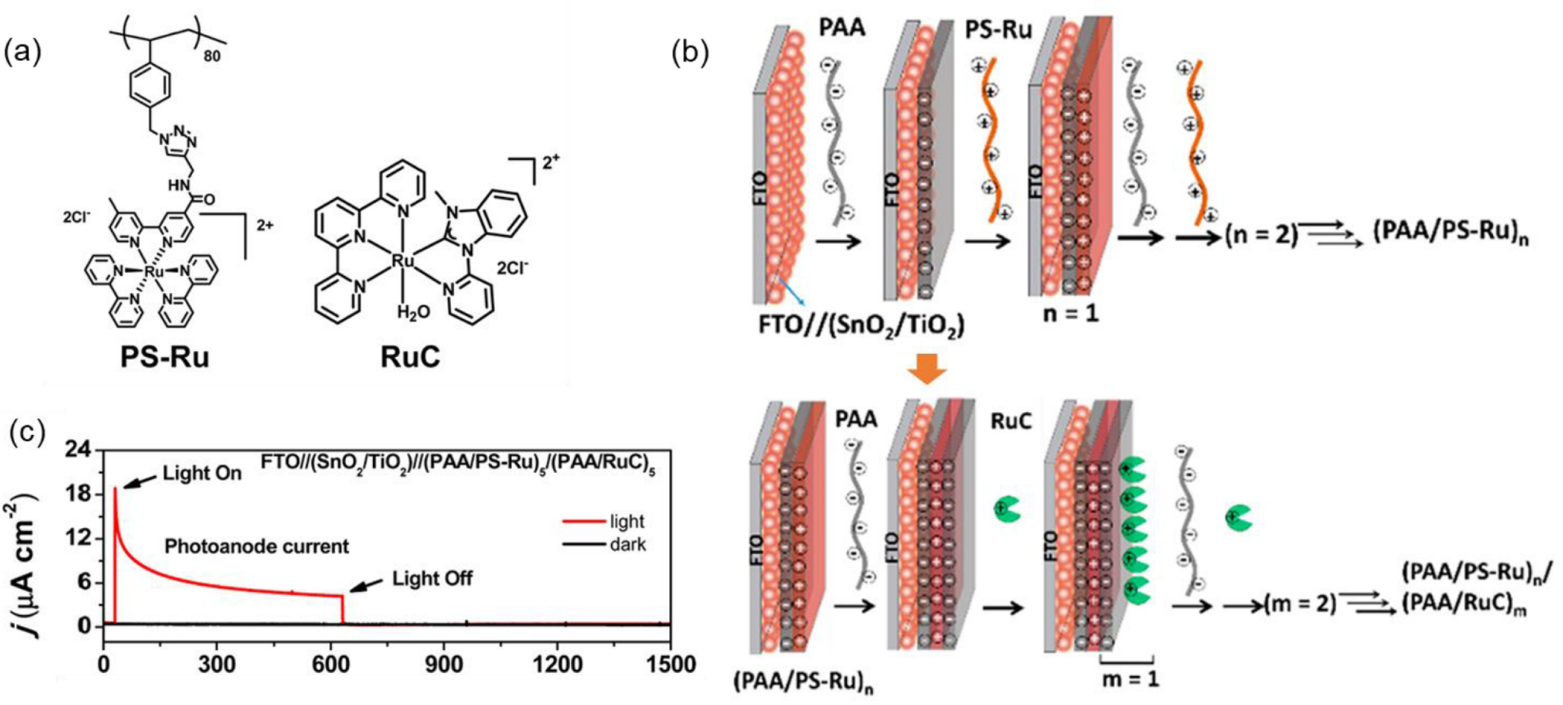
(a) Molecular structures of polystyrene-based PS-Ru and **OER6** (RuC). (b) Schematic illustration for fabrication of (b-top) FTO//(SnO_2_/TiO_2_)//(PAA/PS-Ru)_*n*_ and (b-down) FTO//(SnO_2_/TiO_2_)//(PAA/PS-Ru)_*n*_/(PAA/RuC)_*m*_ multilayer films. (c) Current–time trace with illumination (1 sun, 100 mW cm^–2^, 400 nm cutoff filter) of FTO//(SnO_2_/TiO_2_)//(PAA/PS-Ru)_5_/(PAA/RuC)_5_ photoanode (red) and in the dark (black) in a 0.1 M phosphate buffer at pH 7 with an applied bias of 0.44 V vs NHE. Adapted from ref (193). Copyright 2016 American Chemical Society.

[Ru(tpy)(Mebim-py)(OH_2_)]^2+^ (**OER6** = **RuC**; Mebim-py = 2-pyridyl-*N*-methylbenzimidazole) as another OER catalyst was codeposited with poly(acrylic acid) (PAA), an inert polyanion, to construct chromophore-catalyst assemblies with cationic polystyrene-based Ru polychromophore (PS-Ru) via a LbL self-assembly on the planar SnO_2_/TiO_2_ core/shell structure precoated FTO substrate (Figure 16).^193^ The LbL method enables facile control over the amount and ratio of the chromophore to the catalyst. In the collector-generator (C-G) cell and under irradiation, no measurable cathodic current was detected in the absence of the RuC catalyst layers, while a pronounced anodic photocurrent immediately was produced over FTO//(SnO_2_/TiO_2_)//(PAA/PS-Ru)_5_/(PAA/RuC)_5_. The photocurrent decayed quickly within the first 30 s and sustained at a stable value over the following 530 s of illumination. Prolonged photoelectrolysis experiments revealed O_2_ production from the illuminated photoanode with a FE of 22%, equaling the previously investigated systems using Ru(bpy)_3_-derivatized chromophores with Ru-based OER catalyst stabilized by an atomic layer deposition (ALD) technique.^194^

**Figure 17:**
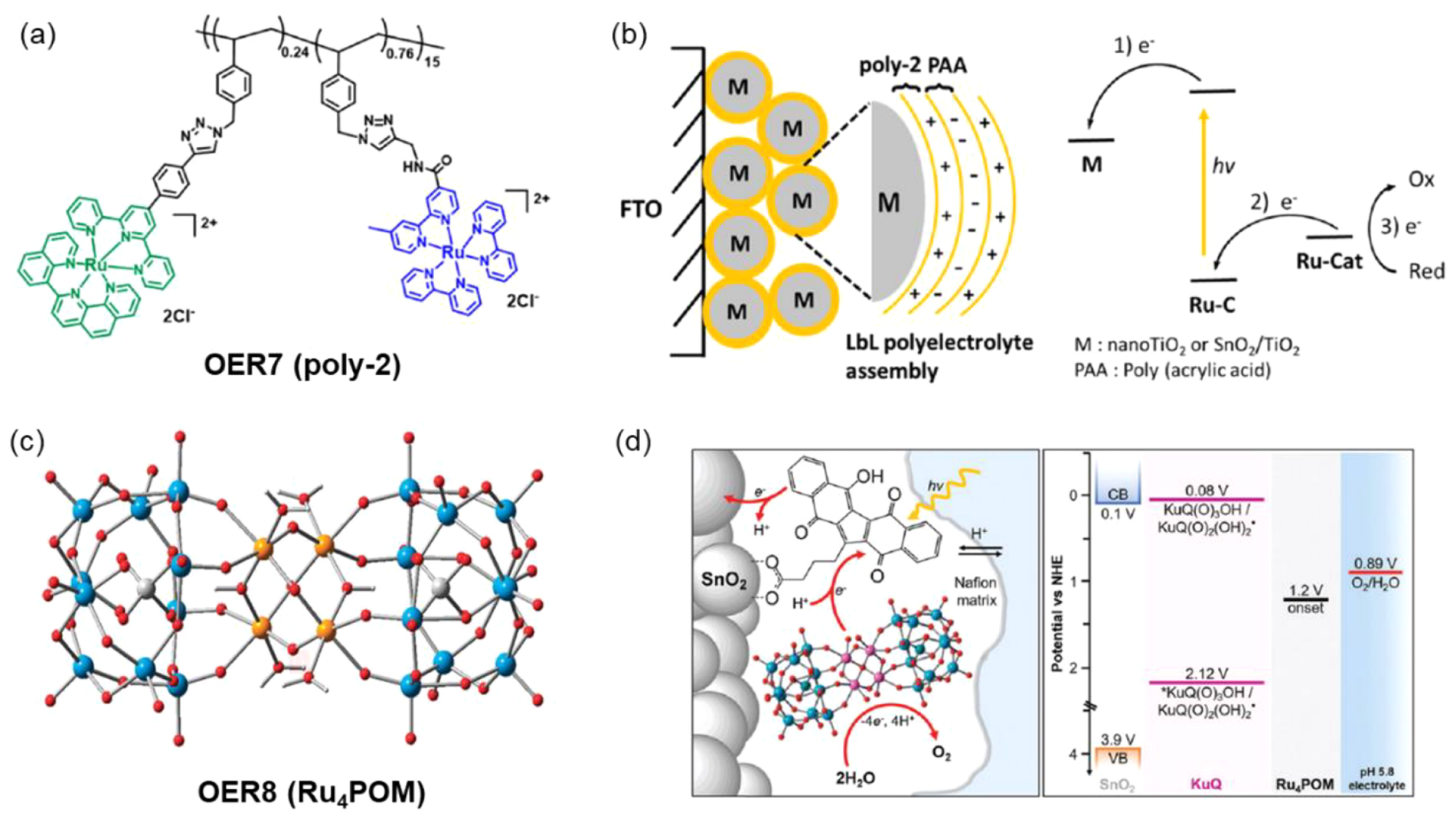
(a) Chemical structure of OER7 (poly-2). (b) Schematic illustration for fabrication of OER7 onto mesoporous substrates. Adapted from ref (195). Copyright 2017 American Chemical Society. (c) Chemical structure of OER8 (Ru_4_POM) (light blue: W; orange: Ru; gray: Si; and red: O). (d) Schematic representation of the SnO_2_|KuQ(O)_3_OH|Ru_4_POM photoanode for water oxidation (the energy levels are shown for the system at pH = 5.8). Adapted with permission from ref (197). Copyright 2020 Royal Society of Chemistry.

Similarly, a polystyrene-based chromophore-catalyst assembly (poly-2) was obtained on a mesoporous metal oxide photoanode also via the LbL method. [Ru(trpy)(phenq)]^2+^ (**OER7** = **RuCat**; trpy = 2,2′;6,2″-terpyridine, phenq = 2-(quinol-8′-yl)-1,10-phenanthroline) and [Ru(bpy)_3_]^2+^ (bpy = 2,2′-bipyridine) derivatives were OER catalyst and chromophores in the assembly, respectively (Figure 17a,b).^195^ Multilayer photoanodes were constructed with cationic poly-2 and anionic PAA. FTO//(SnO_2_/TiO_2_)//PAA/poly-2)_5_ photoanode had an initial photocurrent (∼0.0185 mA/cm^2^) partly ascribed to the light-driven water oxidation (0.5 M KNO_3_ aqueous solution with 0.1 M phosphate buffer (pH 7), 100 μW/cm^2^, λ > 400 nm). The photocurrent generated from bare FTO//(SnO_2_/TiO_2_) was negligible, and the polychromophore-modified photoanode just produced an initial photocurrent density of ∼0.0074 mA/cm^2^ and finally stabilized at ∼0.006.2 mA/cm^2^ after three light on–off cycles. In comparison with the previous multicomponent LbL approach, anchoring Ru catalyst molecules via electrostatic LbL self-assembly,^196^ the LbL films here sustained ∼28% higher photocurrent density after 250 s of continuous illumination. The enhanced photochemical properties of the covalently linked chromophore-catalyst polymer suggested improved charge injection and hole transfer to the catalyst. Polyoxometalate with a tetraruthenium active site, e.g. Na_10_{Ru_4_(μ-O)_4_(μ–OH)_2_[γ-SiW_10_O_36_]_2_}(**OER8** = **Ru**_**4**_**POM**), has the requisite potential for water oxidation.^197−199^ Ru_4_POM was successfully loaded on a KuQ(O)_3_OH (KuQ(O)_3_OH = 1-(3-carboxypropyl)KuQuinone) sensitized SnO_2_ photoanode by soaking in an aqueous solution containing a Nafion polymer and Ru_4_POM (Figure 17c,d).^197^ Absorption spectrum and elemental mapping evidenced the successful and uniform loading of Ru_4_POM over the surface of the SnO_2_|KuQ(O)_3_OH|Ru_4_POM photoanode. Under irradiation, the photocurrent started at a low onset potential of 0.20 V vs NHE, while reaching a net and constant value of 0.020 mA/cm^2^ in the range 0.4–1 V vs NHE. The attribution of the observed photocurrent to O_2_ evolution was confirmed by the C-G cell, where the anodic photocurrent produced at the SnO_2_|KuQ(O)_3_OH|Ru_4_POM generator at 0.8 V vs NHE. The FE for O_2_ evolution was estimated as 70 ± 15%. Conversely, in the case of the catalyst-free SnO_2_|KuQ(O)_3_OH electrode (stationary photocurrent of ca. 0.008 mA), no significant cathodic current for O_2_ reduction was produced, confirming the fundamental role of Ru_4_POM in driving water oxidation. Evidenced by transient absorption spectroscopy (TAS) results, the absence of long-lived dynamics suggests a fast evolution of the dye excited state in the presence of Ru_4_POM and likely involving a charge transfer from Ru_4_POM to *KuQ(O)_3_OH, forming reduced KuQ(O)_2_(OH)_2_* and oxidized Ru_4_POM, and driving water oxidation reaction. A drop of photocurrent density to 60% of the initial value was observed after 30 min, associated with visible leaching of KuQ(O)_3_OH and the Ru_4_POM from the electrode. Furthermore, a remarkably improved PEC water oxidation performance and stability was observed for WO_3_/PPy:Ru_4_POM p-n heterojunction by combining PPy doped with Ru_4_POM (PPy:Ru_4_POM) with WO_3_ photoanode.^198^

#### 4.1.2 Mn or Co-Based Cubane Catalysts

**Figure 18:**
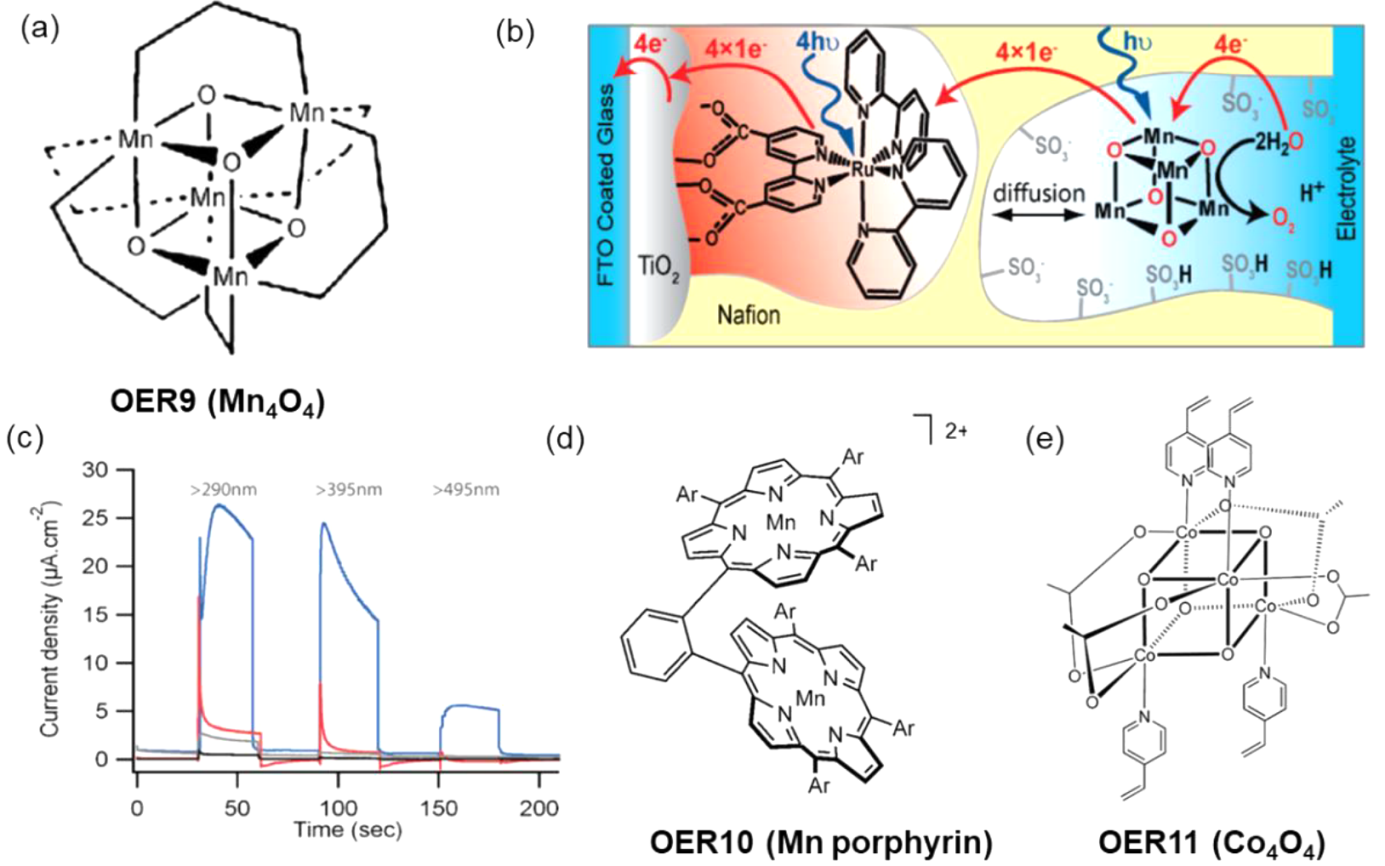
Chemical structures of (a) OER9 (Mn_4_O_4_). (b) Charge transfer route. (c) Photocurrent response of the corresponding photoanode. Representative data from conductive FTO coated glass (black), OER9^+^-Nafion/TiO_2_ (gray), Nafion/photosensitizer-TiO_2_ (red), and OER9^+^-Nafion/photosensitizer-TiO_2_ (blue), illuminated at 100 mW/cm^2^ through a series of long-pass light filters as labeled. Adapted from ref (200). Copyright 2010 American Chemical Society. (d) OER10 (Mn porphyrin; Ar = 4-tBuC_6_H_4_, 2,4,6-Me_3_C_6_H_2_, or C_6_F_5_). Adapted with permission from ref (109). Copyright 2012 Wiley-VCH. (e) OER11 (Co_4_O_4_). Adapted with permission from ref (202). Copyright 2017 Royal Society of Chemistry.

Tetranuclear Mn or Co cubane clusters render water oxidation due to the inspiration of the O_2_-evolving complex composed of four Mn atoms in natural photosystem II. Tetranuclear Mn-oxo cluster ([Mn_4_O_4_L_6_]^+^; L = (MeOPh)_2_PO_2_^–^) OER catalyst has been grafted on [RuII(bipy)_2_-(bipy(COO)_2_)]-sensitized TiO_2_ photoanode by the Nafion polymer (**OER9**, Figure 18a).^200^ The photoanode produced a photocurrent due to water oxidation in an almost neutral solution (0.1 M Na_2_SO_4_, pH = 6.5) under irradiation (λ > 395 nm) without an external bias. Contemporary excitation of the Mn_4_O_4_ catalyst could transfer an electron from the Mn_4_O_4_ cluster to the oxidized sensitizer due to their matched electrochemical potentials and then released O_2_. The Ru^II^ sensitizers injected electrons into the CB of TiO_2_, following transferring electrons to a cathode for H^+^ reduction to H_2_, thereby activating the photochemical water splitting cycle (Figure 18b). The photocurrent was measured ca. 100 times that of bare TiO_2_ (Figure 18c). Later, the Mn-porphyrin monomer (**OER10**, Figure 18d) was uniformly incorporated into a poly(terthiophene) (PTTh) film on the ITO glass or flexible ITO-coated poly(ethylene terephthalate) (PET) sheet to be a photoanode, which possessed the ability to selectively oxidize seawater instead of producing chloride under irradiation.^109^ However, interestingly, monomeric Mn-porphyrin is normally catalytically inactive.^201^

Co_4_O_4_(O_2_CMe)_4_(py)_4_ (**OER11**; py = pyridine derivatives, Figure 18e) with similar merits of Mn cubane catalysts also attracts attention for water oxidation. Several works of cobalt-oxo cubane for photocatalytic water oxidation in homogeneous aqueous solutions consisting of a sacrificial electron acceptor and a photosensitizer have been reported.^203,204^ It was found that the reactivity of cobalt-oxo cubane was relative to the ligand substitution.^205^ However, the reactivity of cobalt-oxo cubane on a polymer photoelectrode aiming at heterogeneous PEC water oxidation is rarely explored. After being immobilized on a Nafion film-coated FTO and an α-Fe_2_O_3_ photoanode, the incorporation of cobalt-oxo cubane catalysts resulted in a significant cathodic shift of onset potential by 400 mV compared to the Nafion-coated α-Fe_2_O_3_ or bare α-Fe_2_O_3_. The photocurrent was increased as much as 6-fold to 0.200 mA/cm^2^ at 0.5 V vs Ag/AgCl with good stability. Furthermore, the vinyl group-modified Co_4_O_4_(O_2_CMe)_4_(4-vinylpy)_4_ (py = pyridine) enabled electrochemical polymerization with vinyl phosphate (Vpa) on a RuP-sensitized TiO_2_ or BiVO_4_ electrode.^202,206^ These two kinds of photoanodes showed enhanced photocurrents and cathodic onset shifts compared to the bare metal oxide photoanodes. Moreover, anchoring linkage Vpa allowed better immobilization of the catalyst on the electrode to promote electron transfer between the sensitizer and the catalyst, ultimately, resulting in better performance and excellent stability.

### 4.2 Inorganic OER Cocatalysts

#### 4.2.1 IrO_*x*_ Inorganic Cocatalysts

IrO_*x*_ is a typical noble metal OER catalyst for improving polymer photoanode performance. For instance, an organic bilayer photoanode, composed of 3,4,9,10-perylenetetracarboxyl-bisbenzimidazole (PTCBI, n-type semiconductor) and 29H,31H-phthalocyanine (H_2_Pc, p-type semiconductor), was capable of O_2_ evolution in water with the assistance of an IrO_2_ catalyst that was encapsulated in a Nafion matrix.^207^ In the electrolyte solution of KOH (pH = 10) and with an applied potential of 0.4 V, O_2_ evolution was only produced with the IrO_2_ catalyst under visible light (<750 nm). IrO_2_ particles were also deposited on a ITO/TiO_2_-PH (PH = polyheptazine) photoanode by soaking in an IrO_2_ colloidal solution, deriving from the hydrolysis of Na_2_IrCl_6_.^208^ Basically, the potential of PH valence band maximum (VBM) was estimated to be positive enough to induce water oxidation. Therefore, under light excitation, ITO/TiO_2_-PH/IrO_2_ had an O_2_ evolution with a photocurrent of ca. 0.1 mA/cm^2^ along with remarkable stability over 90 min (phosphate buffer, +0.5 V vs Ag/AgCl).^208,209^

#### 4.2.2 Co-Based Inorganic Cocatalysts

**Figure 19:**
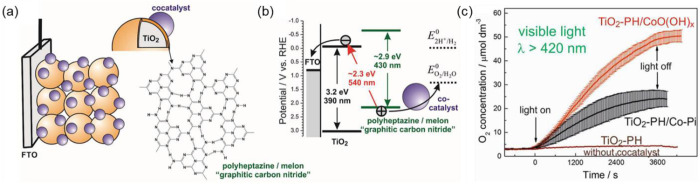
(a) Schematic of TiO_2_-PH hybrid photoanode. (b) Simplified potential scheme illustrating the TiO_2_-PH photoanode under visible light with a cocatalyst. (c) O_2_ evolution of the TiO_2_-PH photoanode without a cocatalyst, with CoO(OH)_*x*_ or Co-Pi cocatalysts. Adapted from ref (211). Copyright 2017 American Chemical Society.

Compared to noble metal cocatalysts, earth-abundant Co-based inorganic catalysts are more attractive and have shown great potential both in electrochemical, photoelectrochemical, and photocatalytic water oxidation, as well as over polymer photoanodes for water oxidation.^108,210−214^ The TiO_2_-PH photoanode has been modified with cobalt oxide via an *in situ* photoelectrochemical deposition method in a phosphate buffer containing Co^2+^ cations (thus, denoted as **Co-Pi**),^210^ ensuring the preferential loading of Co-Pi at the active sites with the highest concentration of photogenerated holes. Under monochromatic light irradiation (λ = 450 nm), the ITO/TiO_2_-PH/Co-Pi photoanode produces a photocurrent density of ∼0.1 mA/cm^2^, higher than that of the ITO/TiO_2_-PH photoanode (+0.5 V vs Ag/AgCl). Under visible light (λ > 420 nm), the photocurrent density further increased to 0.19 μA/cm^2^, capable of producing O_2_ continuously for 1 h 40 min, while no O_2_ was detected in the absence of Co-Pi. Substituting Co-Pi particles (∼5 nm) by smaller **CoO(OH)**_*x*_ particles (∼1–2 nm) is beneficial for improving activity and stability (Figure 19).^211^ In detail, the CoO(OH)_*x*_ cocatalyst was chemically deposited on the FTO/TiO_2_-PH by successive immersion in a solution of Co(NO_3_)_2_ and a weakly basic aqueous ammonia. Due to the smaller size, higher loading of CoO(OH)_*x*_ than Co-Pi provides more electroactive surface area without decreasing transparency, which would affect the excitation of TiO_2_-PH and following photocatalytic processes. As a result, the FTO/TiO_2_-PH/CoO(OH)_*x*_ (34%) outperformed the FTO/TiO_2_-PH/Co-Pi (17%) with a photocurrent of higher than 0.11 mA/cm^2^ after 4 h visible light irradiation.

**Ni-Co** bimetallic nanoparticles have been developed as an OER catalyst that is directly loaded on an n-type π-conjugated naphthalene benzimidazole polymer photoanode (poly[benzimidazobenzophenanthroline], also labeled as BBL) by the solvothermal method.^108^ However, no O_2_ was generated due to the poor catalyst attachment with BBL. After depositing a tunnel junction of an ∼1 nm thin TiO_2_ layer on the BBL film via ALD to better attach Ni–Co nanoparticles, ultimately, the photocurrent density was improved from ∼0.015 to 0.030 mA/cm^2^. The O_2_ evolution was confirmed during the constant illumination with a FE of 82 ± 16%. Recently, Co^2+^ salt-modified S-gC_3_N_4_/BiOCl, prepared by a simple ultrasonically aided hydrothermal method, was reported to have an evident enhancement of the photocurrent density,^212^ which was measured as 0.393 mA/cm^2^ for Co-S-gC_3_N_4_/BiOCl, ∼3-fold higher than S-gC_3_N_4_/BiOCl (1.23 V vs RHE). Similarly, a Ni-based inorganic OER catalyst also has enormous potential to modify polymer photoanodes; the yet reported candidates are just briefly summarized here, including NiOOH,^215^ Ni(OH)_2_,^216^ Ni salt,^217^ NiFeO_*x*_,^218^ NiO_*x*_,^219^ and the Ni-Co catalyst.^108^

### 4.3 Molecular HER Cocatalysts

**Figure 20:**
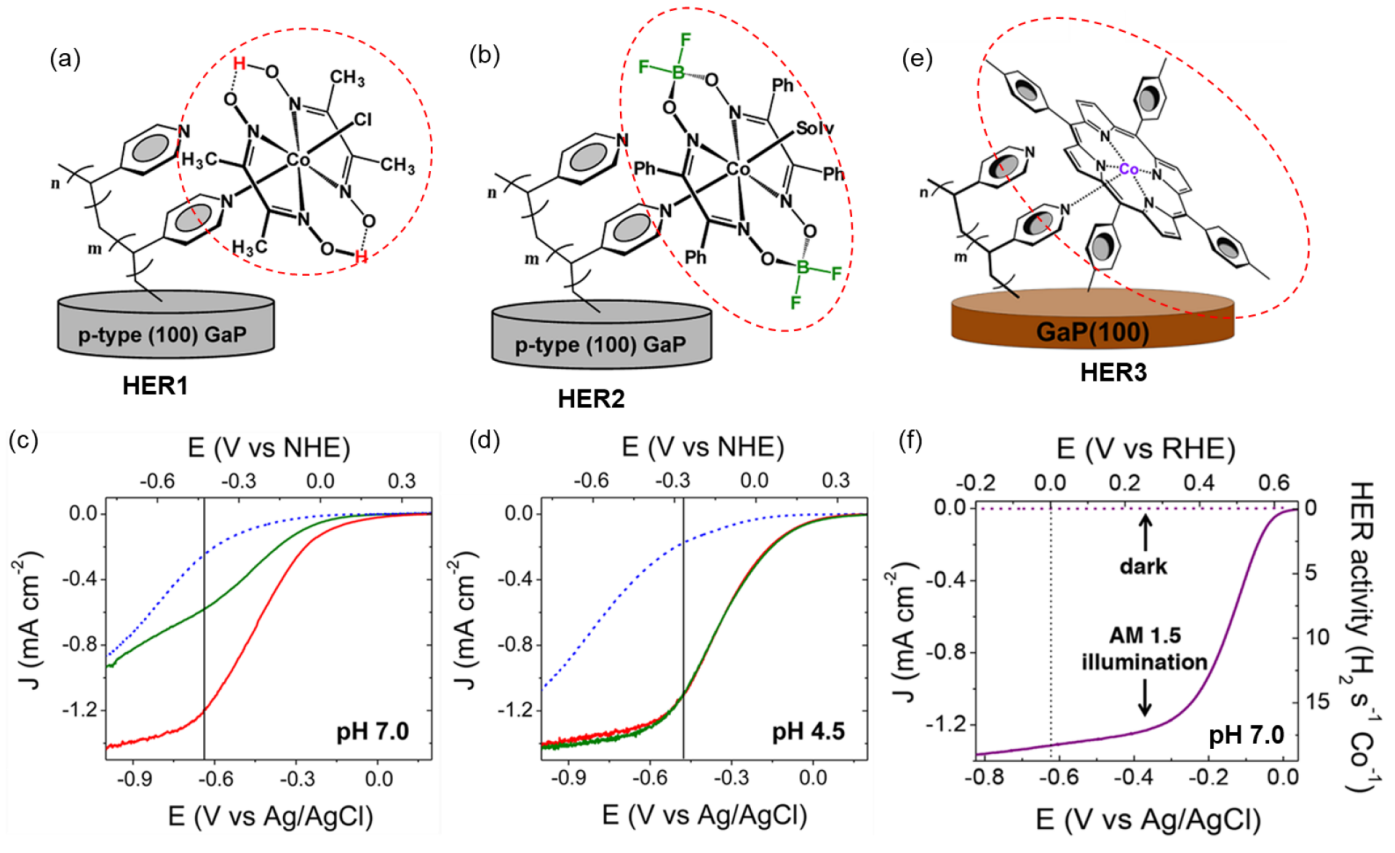
Chemical structure (marked by red circle) of (a) cobaloxime (**HER1**, Co(dmgH_2_)(dmgH)Cl_2_). (b) Boron difluoride modified HER1 (**HER2**). (c) Linear sweep voltammograms (LSVs) of GaP (blue dash), HER1 loaded GaP (red), and HER2 loaded GaP (green) recorded at pH 7. (d) Recorded at pH 4.5 under simulated AM 1.5 illumination. Adapted from ref (223). Copyright 2014 American Chemical Society. (e) Cobalt porphyrin (**HER3**, CoTTP). (f) LSV of HER3 loaded GaP at pH 7 under simulated AM 1.5 illumination. Adapted from ref (226). Copyright 2017 American Chemical Society.

Up to now, Co complexes are the most widely investigated molecular catalysts to facilitate PEC water reduction. Thereinto, the efficient cobaloxime family contains a coordinated Co^III^ ion as the redox platform and an −OH group in the second coordination sphere as the proton relay site.^220^ They have been grafted on the p-type GaP photocathode via the polymerization method and systematically applied for PEC water reduction.^221−223^ In a typical preparation procedure, the surface oxide of the GaP electrode was first etched by buffered hydrofluoric acid (HF) to create OH-terminated sites for UV-initiated photochemical attachment of linker molecules.^221^ Then, polymer growth was achieved by reacting 4-vinylpyridine with the hydroxylated surface. The self-initiated photografting and photopolymerization mechanism have been reported on a variety of hydrogen- and OH-terminated materials and occur when hydrogen is abstracted from the surface by a photoactivated monomer to begin surface-initiated free radical polymerization. In this way, polymers bear multiple pyridine binding sites per chain, thereby promising the geometric area loading capacity of catalysts. The catalyst attachment occurs by replacement of one of the axial chloride ligands of Co(dmgH_2_)(dmgH)Cl_2_ (**HER1**) (Figure 20a,c) with a surface-attached pyridine moiety. Compared to bare GaP, the Co-functionalized photocathode showed significantly enhanced PEC performance of 2.4 mA/cm^2^ photocurrent density at 0.17 V vs RHE (neutral pH, AM 1.5 illumination). By introducing a boron difluoride (BF_2_) capping group on the glyoximate ligand of cobaloxime (**HER2**) (Figure 20b,d), the PEC performance could be notably influenced by the change of the ligand environment of molecular catalysts.^223^ Then, to unveil the effect of the polymeric interface, polyvinylimidazole (PVI) and PVP, providing pyridyl or imidazole ligands, were employed to graft **HER2** on GaP(100) electrodes.^224^ The photocathodes with imidazole and pyridyl immobilized cobaloximes were both capable of achieving a photocurrent density ∼1 mA/cm^2^ at +0.24 and +0.07 V vs RHE, respectively. The per-cobalt TOF of the Co-PVP-GaP and Co-PVI-GaP photocathodes were estimated as 2.1 s^–1^ and 2.4 s^–1^, respectively (0 V vs RHE). Subsequently, the **HER2** catalyst was immobilized onto two sides of a p-type GaP substrate via coordination to a surface-grafted PVI brush.^225^ Through a similar way, cobalt porphyrin catalysts (5,10,15,20-tetra-p-tolylporphyrin cobalt(II)) (CoTTP, **HER3**) (Figure 20e,f) were assembled on the thin-film polypyridine (4-vinylpyridine as the monomeric unit) surface coatings with a molecular interface for H_2_ evolution onto a visible-light absorbing p-type GaP semiconductor.^226−229^

**Figure 21:**
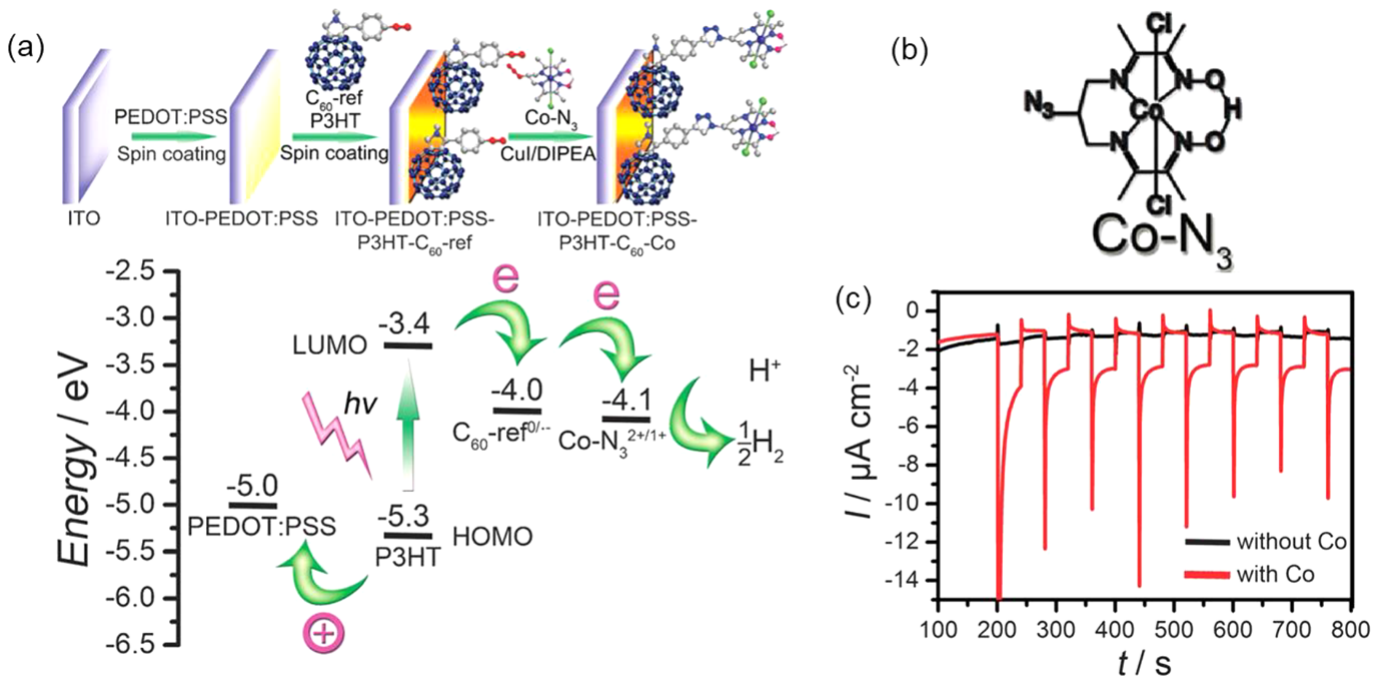
(a, top) Preparation of the OPV-based PEC electrode with a Co catalyst by “click” chemistry. (a, down) The energy level diagram depicts the relevant energy levels under flat band conditions of all materials used in the photocathode. (b) Chemical structure of Co-N_3_ (**HER4**). (c) Transient photocurrent response curves of the OPV photocathodes with and without the Co catalyst. Adapted with permission from ref (231). Copyright 2015 Royal Society of Chemistry.

Adopting “click” chemistry for electrode immobilization of a cobaloxime catalyst, diamine-dioxime cobaloxime (**HER4**, Co-N_3_) could be successfully grafted to a fullerene derivative (*N*-methyl-2-(4′-ethynyl)phenyl-3,4-fulleropyrrolidine, C_60_-ref) on an organic photovoltaic (OPV) electrode (Figure 21).^230,231^ The LUMO of poly(3-hexylthiophene-2,5-diyl) (P3HT) is more negative than the reduction potential of C_60_-ref, thus charge transfer between P3HT and C_60_-ref is thermodynamically feasible. In accordance with the electrochemical measurements, the energy levels of C_60_-ref^0/–^ and Co-N_3_^2+/1+^ were estimated to be −4.0 and −4.1 eV, respectively.^232^ Therefore, an intermolecular charge transfer between the reduced C_60_-ref and **HER4** is expected to reduce protons to H_2_.^230^ Compared to ITO/PEDOT:PSS/P_3_HT:C_60_-ref, the ITO/PEDOT:PSS/P_3_HT/C_60_-Co photocathode had a much higher photocurrent density as shown in Figure 21c (0.1 V vs NHE, 100 mW/cm^2^), confirming the good catalytic effect of **HER4**.

### 4.4 Inorganic HER Cocatalysts

#### 4.4.1 Pt

**Figure 22:**
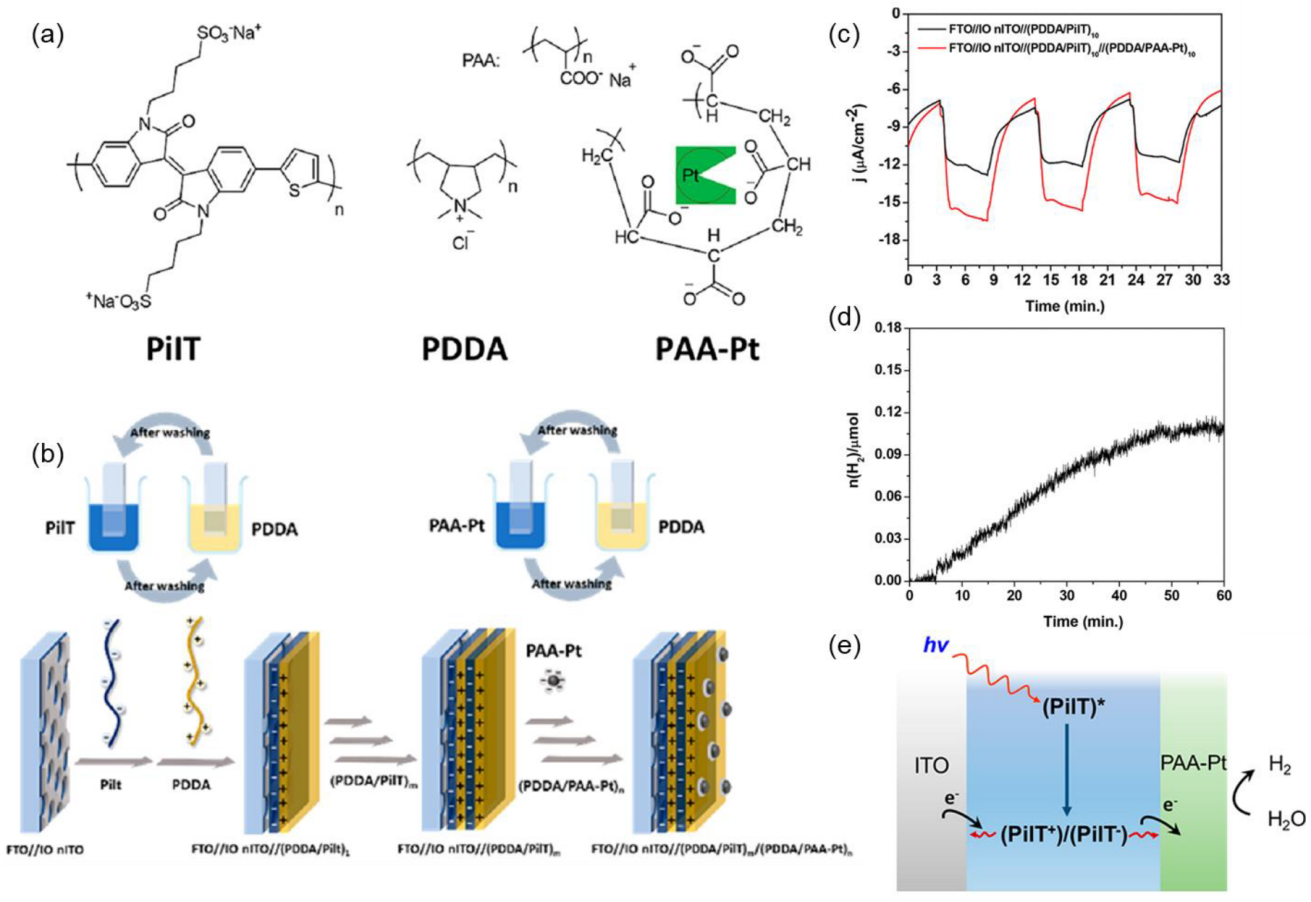
(a) Molecular structures of PilT, poly(diallyldimethylammonium chloride) (PDDA), and polyacrylatestabilized Pt nanoparticles (PAA-Pt). (b) Schematic illustration of fabrication of FTO//IOnITO//(PDDA/PilT)_10_//(PDDA/PAAPt)_10_. (c) Current–time traces with illumination on FTO//IO nITO//(PDDA/PiIT)_10_ (black) and FTO//IO nITO//(PDDA/PiIT)_10_//(PDDA/PAA-Pt)_10_ (red) in 0.1 M acetate buffer, 0.4 M NaClO_4_, at pH 4.5 with an applied bias of −0.4 V versus Ag/AgCl. (d) H_2_ production versus time. (e) Proposed mechanism for charge generation/separation in PiIT/PAA-Pt films. Adapted with permission from ref (233). Copyright 2018 American Chemical Society.

Noble metal Pt is the most efficient and extensive inorganic HER catalyst and shows significance for decorating polymer photocathodes. As a special instance, Pt-nanoparticles stabilized by PAA (PAA-Pt) and the anionic poly(isoindigo-*co*-thiophene) with pendant sodium butylsulfonate groups (PiIT) polyelectrolyte PAA-Pt were codeposited with cationic poly(diallyldimethylammonium) chloride (PDDA) by using LbL self-assembly onto inverse opal (IO) and nanostructured FTO//ITO (nITO) (Figure 22).^233^ Photoelectrodes with PAA-PT modification showed an enhanced cathodic photocurrent, pointing toward the photoinduced electron transfer from the excited PilT to PAA-Pt and a fast HER rate. Photocatalytic H_2_ production was confirmed in pH 4.5 acetate buffer with NaClO_4_ under visible-light irradiation. 0.11 μmol of H_2_ was produced with 0.047 C of cathodic charge, thus the corresponding FE was ∼45%.

**Figure 23:**
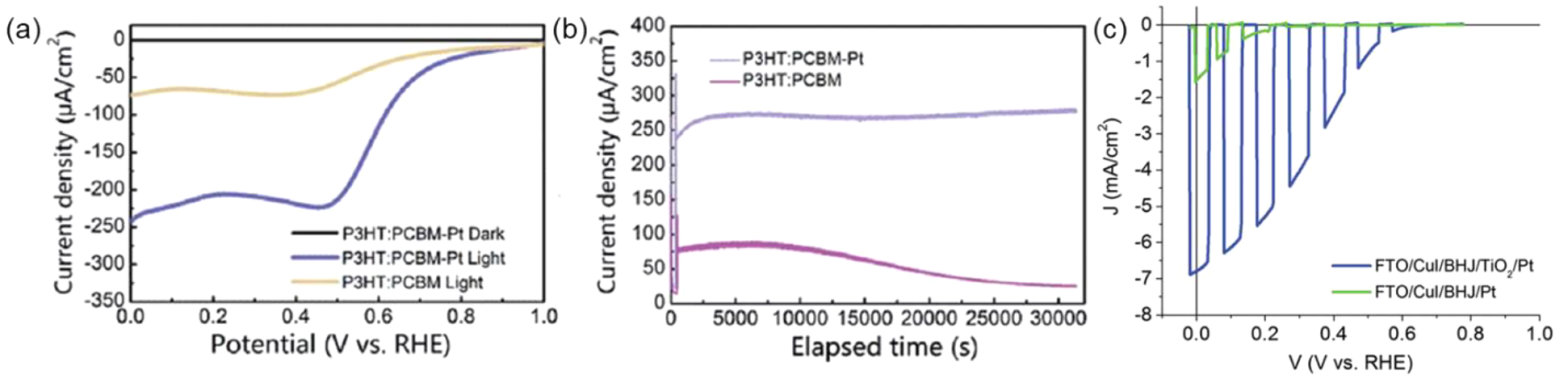
(a) Photocurrent density of the photocathodes vs the applied voltage. (b) Prolonged *J*–*t* curves of the photocathodes at a bias potential of 0 V vs RHE. Adapted with permission from ref (234). Copyright 2018 Royal Society of Chemistry. (c) Chopped photocurrent density comparison of photoanodes with or without a TiO_2_ layer. Adapted with permission from ref (148). Copyright 2016 Royal Society of Chemistry.

In addition, Pt catalyst deposited on a p-type organic photocathode based on P3HT:PCBM BHJ also significantly improved the water reduction performance.^146,148,234−238^ In detail, after directly depositing a thin layer of Pt onto the top of the P3HT:PCBM (PEDOT:PSS/P3HT:PCBM-Pt), the photocathode represented an increased water reduction onset potential to about +0.69 V vs RHE and the 0.24 mA/cm^2^ photocurrent density with good stability over 9 h irradiation (0 V vs RHE) (Figure 23a,b). The Pt layer not only improved the charge transport and separation at the Pt/electrolyte interface but also served as electron sinks to effectively extract the electrons from the LUMO of P3HT:PCBM to restrain electron–hole recombination inside the organic semiconductor.^234^ The photocurrent was further improved via coating a protective TiO_2_ layer between BHJ and Pt to reduce carrier recombination and to enhance electron collection on Pt (Figure 23c).^147,148^ In the CdSe and P3HT organic–inorganic photocathode, a thin Pt cocatalyst layer also notably promoted the photocurrent from 0.16 to 1.24 mA/cm^2^.^239^ Moreover, obvious enhancement was observed after changing the polymer layer by PTB7:PCBM (PTB7 = poly[(4,8-bis(2-ethylhexyloxy)-benzo(1,2-b:4,5-b’)dithiophene)-2,6-diyl-*alt*-(4-(2-ethylhexyl)-3-fluorothieno[3,4-*b*]thiophene-)-2-carboxylate-2–6-diyl]),^240^ or PBDB-T:ITIC (PBDB-T = poly[(2,6-(4,8-bis(5-(2-ethylhexyl)thiophen-2-yl)-benzo[1,2-b:4,5-b’]dithiophene))-*alt*-(5,5-(1′,3′-di-2-thienyl-5′,7′-bis(2-ethylhexyl)benzo[1′,2′-c:4′,5′-c′]dithiophene-4,8-dione))]; ITIC = 3,9-bis(2-methylene-(3-(1,1-dicyanomethylene)-indanone))-5,5,11,11-tetrakis(4-hexylphenyl)-dithieno[2,3-d:2′,3′-d′]-s-indaceno[1,2-b:5,6-b′]dithiophene).^241^ In addition, there are many other studies relating to the Pt cocatalyst to improve the performance of polymer photocathodes.^242−246^ Similarly, RuO_*x*_ is another extensively used noble HER catalyst for decorating the polymer photocathode, and corresponding work has been reviewed here.^117,247,248^

#### 4.4.2 MoS_*x*_

As discussed previously, exploring earth-abundant cocatalysts is of great significance for large-scale solar energy conversion.^249,250^**MoS**_*x*_, in particular of MoS_3_, is the most widely investigated catalysts to modify polymer photocathode for water reduction. By adding aqueous HCl into MoO_3_ and Na_2_S suspension, MoS_3_ particles could be successfully synthesized according to the reported method.^144,251^ Then MoS_3_ or mixed TiO_2_:MoS_3_ were combined with P3HT:PCBM to construct photocathodes for PEC water reduction. Under visible light irradiation, the photocurrent of MoS_3_/P3HT:PCBM photocathode (0.03 mA/cm^2^) was only slightly higher than the one without a catalyst layer (0.025 mA/cm^2^) (0.5 M H_2_SO_4_ aqueous solution, −0.05 V vs Ag/AgCl). By contrast, the photocathode with another cocatalyst TiO_2_:MoS_3_ layer yielded a higher photocurrent (>0.1 mA/cm^2^). Similar to the Pt cocatalyst, introducing an electron-collecting layer, routinely used in OPV technology,^252^ between the P3HT:PCBM and MoS_3_ layers also contributes to enhancing the overall performance. To prove this concept, an aluminum layer was deposited between the cocatalyst and the polymer layer. Moreover, a metallic LiF/Al/Ti layer or C_60_ layer was introduced to improve the stability and catalytic performance.^145^

Besides the surface deposition, the MoS_*x*_ cocatalyst could also be electrodeposited into a photosensitive Ru complex film by electropolymerization of a pyrrole-functionalized Ru^II^(2,2′-bipyridine)_3_^2+^.^156^ The MoS_4_^2–^ was first incorporated into polyRu by ion exchange and then electroreductive transformation in the film to MoS_*x*_. For the polyRu photocathodes, no H_2_ was detected without light irradiation or in the absence of MoS_*x*_. In the presence of MoS_*x*_ and under visible light, the HER process certainly occurred via the oxidative quenching of the excited state of the Ru complex by the MoS_*x*_ catalyst, thereby exhibiting an enhanced and stable PEC H_2_ evolution activity.

### 4.5 CRR Cocatalysts

Ru- and Re-based molecular complexes have been developed as cocatalysts to improve the CRR performance of the polymer photocathode. Only Ru and Re complexes are reviewed in the following, because, to the best of our knowledge, inorganic catalysts have not raised much attention in this field.

#### 4.5.1 Ru-Based Molecular Cocatalysts

**Figure 24:**
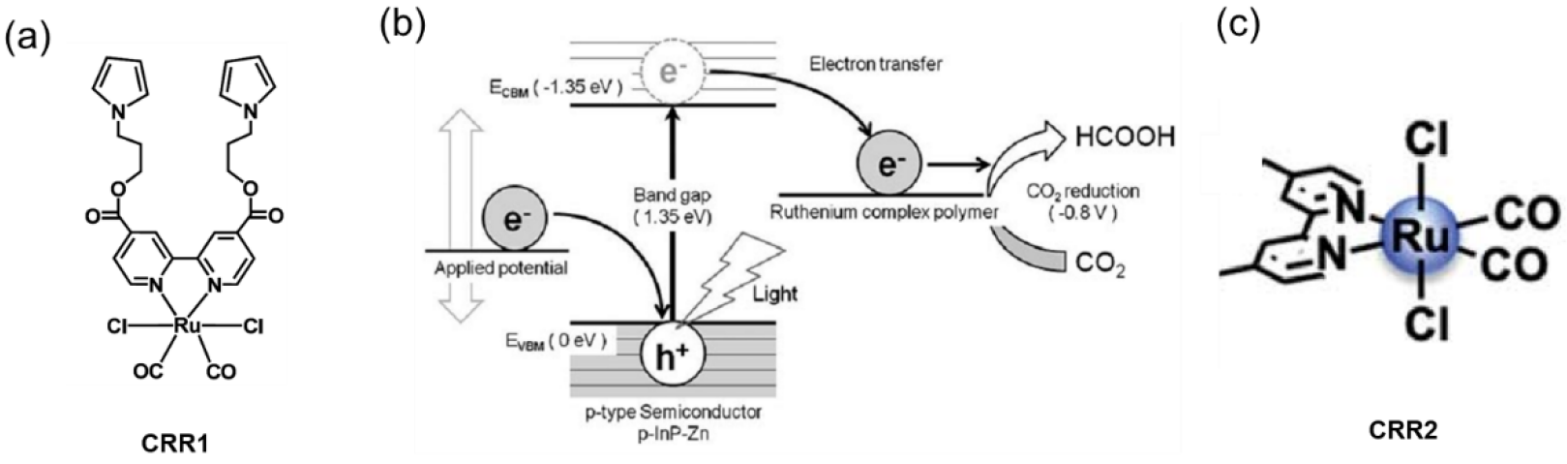
(a) Chemical structure of CRR1. (b) Schematic energy diagram of an RCP/p-InP-Zn electrode under visible-light irradiation. Adapted with permission from ref (253). Copyright 2010 Royal Society of Chemistry. (c) Chemical structure of CRR2. Adapted with permission from ref (256). Copyright 2016 Royal Society of Chemistry.

Ruthenium-complex polymer [Ru(L-L)(CO)_2_]_*n*_ (RCP; L-L = 4,4′-diphosphate ethyl-2,2′-bipyridine) has been developed as a CRR catalyst on the p-type InP:Zn (zinc-doped indium phosphide, p-InP-Zn) photocathode by the *in situ* photoelectropolymerization of **CRR1** (Figure 24a).^253^ Proper potential positions of p-InP-Zn, RCP, and the CO_2_ redox reaction ensure the efficient transfer of photoexcited electrons from p-InP-Zn to RCP and the reduction of CO_2_ to formate (HCOOH) (Figure 24b). The current efficiency for formate formation (EFF) over RCP/p-InP-Zn at pH 4 was measured as 34.3%. Then, three other **CRR1** derivatives were designed for polymer RCP/p-InP photocathodes preparation. The selectivity of HCOO^–^ was further improved beyond 70%, and the conversion efficiency of solar energy to chemical energy was achieved to be 0.03–0.04%.^254^ Finally, a SrTiO_3_ photoanode was used to combine with this photocathode as a wireless device, which successfully performed solar CO_2_ reduction and yielded a solar conversion efficiency of 0.08%.^255^

Recently, a new three-step procedure for preparing the highly stable photoelectrochemical CRR photoelectrode comprising a NiO substrate and polymerized complexes of the Ru(II) photosensitizer and a Ru(II) cocatalyst was reported.^257^ The Ru trisdiimine type photosensitizer (PRuV) contains both methyl phosphonic acid groups and vinyl groups on the diamine ligands and was adsorbed by methyl phosphonic acid groups on the NiO electrode. Another Ru mononuclear complex possessing diimine ligands with a vinyl group and a noncoordinated diimine ligand (VRu-N^N) was connected by electropolymerization of the vinyl groups. These procedures induced the rigid attachment of the polymerized Ru redox photosensitizer with a noncoordinated diimine ligand onto the NiO electrode and increased the amount of the attached Ru photosensitizer units. Finally, a Ru cocatalyst unit of **[Ru(CO)**_**2**_**Cl**_**2**_**]**_***n***_ (**CRR2**) (Figure 24c)^256^ was introduced into the noncoordinated diimine ligand to form a [(N^N)_2_-Ru(bpyC_2_bpy)Ru(CO)_2_Cl_2_]^2+^-type chromophore-catalyst complex on the electrode, which has shown durable CRR ability in homogeneous solutions or hybrid systems with various semiconductor particles.^256,258−260^ Under visible light irradiation (460 nm < λ < 650 nm, 20 mW cm^–2^), the NiO/PRu-poly-Ru-RuCAT1 molecular photocathode displayed excellent stability and selectivity for CO and HCOOH production over 100 h in 50 mM NaHCO_3_ aqueous solution (−0.7 V vs Ag/AgCl). The TON of CRR products exceeded 1200, being the outstanding activity for PEC reactions. Furthermore, a connected device of this polymer photocathode with a CoO_*x*_/BiVO_4_ photoanode, suitable for water oxidation, was developed to facilitate stable solar energy-driven CRR by using water as an electron donor, while a low-energy conversion efficiency of 1.7 × 10^–2^% was obtained.

#### 4.5.2 Rebased Molecular Cocatalysts

**Figure 25:**
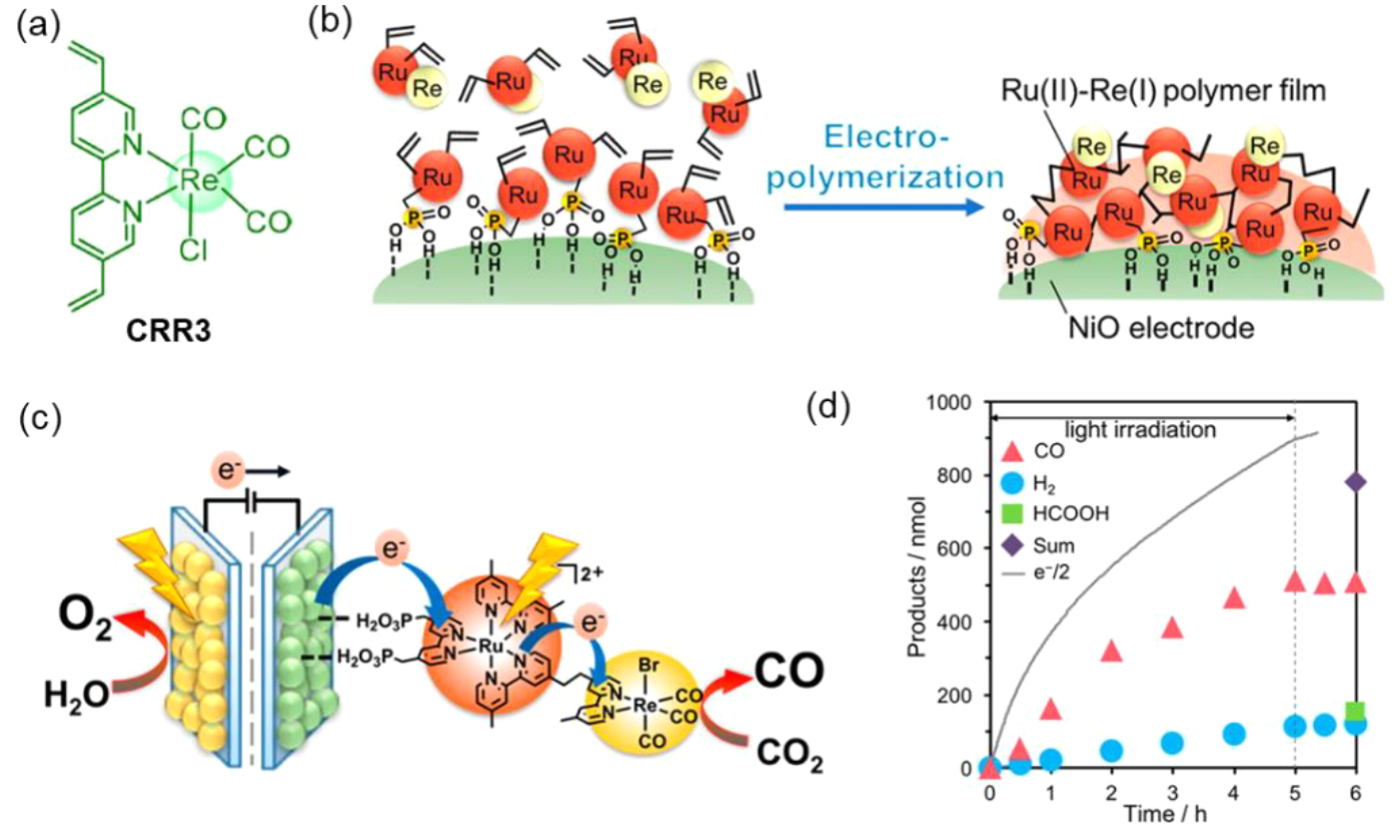
(a) Chemical structure of CRR3. (b) Preparation of the poly-RuRe/NiO electrode by electropolymerization. (c) Photoelectrochemical CO_2_ reduction system using H_2_O as a reductant. (d) Time courses of CO (red ▲), H_2_ (blue ●), HCOOH (green ■), and half amounts of electrons (black line) passed through the poly-RuRe/NiO (2.5 cm^–2^) at *E* = −0.7 V vs Ag/AgCl under irradiation at λ_ex_ > 460 nm. CO_2_ purged 50 mM NaHCO_3_ (aq) (pH 6.6) was used as an electrolyte. Adapted from ref (159). Copyright 2019 American Chemical Society.

The Re(I) bipyridine tricarbonyl chloride complex (denoted as **Re(bpy)(CO)**_**3**_**A**; A = Cl or Br) (**CRR3,**Figure 25a) is one of the extensively studied CO_2_ reduction catalysts both in electrocatalytic and photocatalytic systems. In particular, the heterogenization of Re(bpy)(CO)_3_Cl has been successfully applied to a wide range of surfaces through both noncovalent and covalent interactions.^261,262^ In a heterogeneous polymer system, a stable and efficient molecular photocathode (poly-RuRe/NiO) exists by stabilizing the Ru(II)–Re(I) supramolecular photocatalyst encapsulated **CRR3** on the p-type NiO electrode via electrochemical polymerization (Figure 25b–d).^159^ Due to the coexistence of the vinyl groups in the diimine ligand and methyl phosphonic acid anchors, the new poly-RuRe/NiO photocathode adsorbed more metal complexes and displayed better stability compared to that only using methyl phosphonic acid anchor groups. Compared to the unmodified electrode, the poly-RuRe/NiO photocathode produced approximately 2.5 times more CO (300 W Xe lamp with λ > 460 nm, 50 mM NaHCO_3_ aqueous solution, −0.7 V vs Ag/AgCl), and the FE was improved from 57 to 85%.

Recently, a variety of supporting materials were successfully modified with Re(bpy)(CO)_3_Cl by a surface-localized electropolymerization method.^263^ The coordination environments of the rhenium bipyridine tricarbonyl sites are preserved upon immobilization, and the polymerized cocatalyst moieties exhibit long-range structural order with uniform film growth. Though at a low cocatalyst loading, **CRR3** modified TiO_2_ photocathode demonstrates an enhanced activity with TON up to 70 during 5 h. Then, **CRR3** and [Ru(bpy)_3_]^2+^ (RuL) were coembedded in a polymer Nafion matrix.^264^ The ternary **CRR3**-RuL-Nafion system exhibited higher photoconversion of CO_2_ and photostability than the homogeneous RuL-**CRR3** system without Nafion. The Nafion could connect RuL sensitizers and hinder the destructive self-sensitized reaction via enhancing the electron transfer from excited RuL to **CRR3**. The TON in the **CRR3**-RuL-Nafion system was 454 for a 20 h reaction, which was ca. 4 times higher than that in the RuL-CRR3 system.

**Figure 26:**
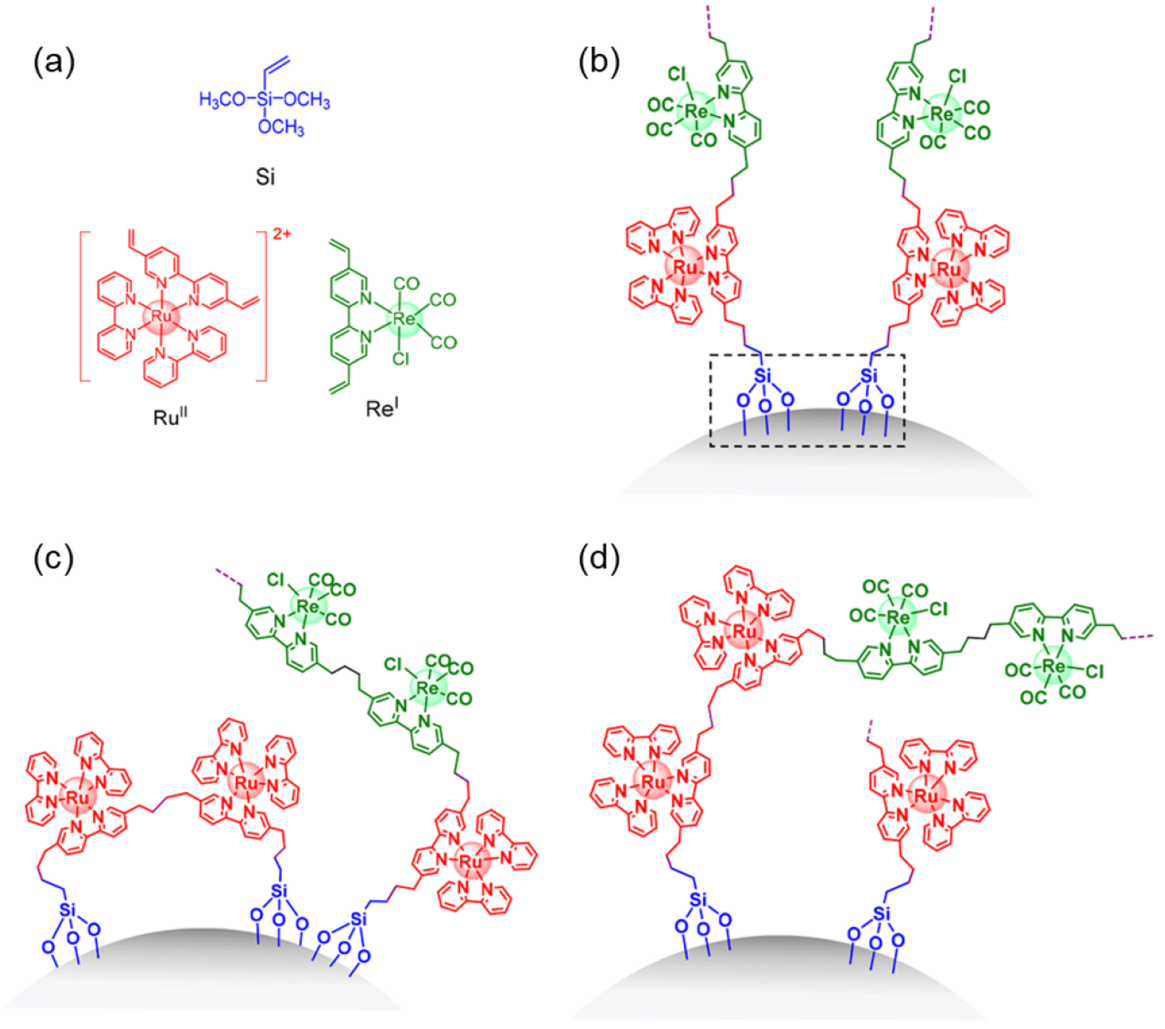
(a) Molecular structures for the surface bridge (Si), the chromophore (Ru^II^), and the catalyst (Re^I^). (b–d) Possible surface assembly structures on NiO|Si–poly(Ru^II^)–poly(Re^I^). Adapted from ref (160). Copyright 2019 American Chemical Society.

A photocathode assembly (NiO|Si–poly(Ru^II^)–poly(Re^I^)) by silanization of NiO and a two-step electropolymerization is shown in Figure 26. Vinyl groups functionalized molecular components of silane surface bridge, chromophore, and the catalyst are vinyltrimethoxysilane (Si), [Ru(dvb)_2_bpy]^2+^ (Ru^II^; dvb = 5,5′-divinyl-2,2′-bipyridine), and [Re(dvb)(CO)_3_Cl] (Re^I^), respectively. NiO|Si–poly(Ru^II^)–poly(Re^I^) had a stable activity toward CRR over 10 h with a FE of ∼65% and a TON of 58. The long-term stability arises from the silane surface-anchoring groups and the formation of C–C bonds between the three components due to the electropolymerization approach. It was revealed that excitation of the chromophore was followed by rapid hole injection into NiO and the catalyst reduction. The relatively slow interfacial back electron transfer from the reduced catalyst to NiO, in turn, facilitates electron transfer toward CRR.^160^

Overall, the cocatalyst, no matter molecular or inorganic ones, dramatically improved both oxidation and reduction activity of a polymer photoelectrode by about 10-fold in most cases. More importantly, the stability of the polymer photoelectrodes was enhanced greatly. However, one can see that the scale of the photocurrent density of the cocatalyst-loaded polymer photoelectrodes is mainly at a few mA/cm^2^, still smaller than the inorganic counterparts (about tens of mA/cm^2^)^265^ under similar experimental conditions.

## 5 Charge Carrier Dynamics of Polymer Photoelectrodes

The recent development of polymer-based photoelectrodes for OER, HER, and CRR has dramatically outpaced our fundamental understanding of their inner workings. The synthetic techniques and material discovery strategies have benefitted from a large body of previous work in organic photovoltaics^266−268^ because of the similarities in the materials targeted. In contrast, the aqueous interface in organic PEC devices adds considerable challenges to spectroscopic investigations. The aqueous environment is vastly different from the solid-state polymer environment, leading to shifts in spectral signatures derived from thin films for photovoltaics and photoinduced protonation changes that are hard to anticipate.^269^ The collective pathways taken by charges and their kinetics termed charge carrier dynamics ultimately dictate the efficiency and performance of the photochemical systems.^270^ Knowledge of the charge carrier dynamics provides a pathway to gain key insights into the limitations of a system. As the time scales (kinetics) of different photophysical processes are determined, one can identify the most restrictive limitation. This information could be used to guide efforts on impactful modifications that may be incorporated in the design of more efficient PEC systems.

In this section, we review charge carrier dynamics studies of organic photoelectrodes for HER and OER in the context of designing next-generation systems. Of note, to the best of our knowledge, there are no charge carrier dynamics studies of CO_2_ reduction on organic photoelectrodes that highlights the limitations of our current understanding, which will be underlined at the end of the section.

Several approaches to the design of semiconductor-based devices for solar fuel generation have been proposed and tested.^271^ The two most relevant for organic photoelectrodes are PEC cells^272^ and PV-driven electrolytic cells.^273,274^ These approaches are distinguished from the source of the asymmetry that separates photogenerated charge carriers and the types of interfaces. PEC photoelectrodes function as monolithic devices where charge carriers are separated at the solid|electrolyte interface. The photovoltage and photocurrent generated by PEC devices under illumination are due to charge separation moderated by differences in electrochemical potentials at the semiconductor|electrolyte interface or by asymmetries in the charge-transfer kinetics for charge carriers across the junction. Typical PV-driven electrolytic cells consist of buried junctions arranged electrically in series with electrocatalysts immersed in an electrolyte. The electrocatalyst can either be in physical contact with the photoelectrode or can be electrically connected by wires, with the PV device completely separated from the electrolyte.^275,276^ Here the photovoltage and photocurrent produced under illumination originate from charge separation mediated within the PV cell and, hence, are independent of the nature of the electrocatalyst|electrolyte interface. It is generally thought that this working principle is akin to that of PV cells, and consequently, concepts of OPVs are applicable.^277^ Because of this, we shall not consider PV-driven electrolytic cells in this section and will focus on PEC devices and the understanding of architectural modifications such as heterojunction formation, porosity, and crystallinity, coinciding with the contents addressed in the previous sections. Before discussing these characteristics, we first discuss mechanistic distinctions between inorganic and organic PEC cells.

### 5.1 Mechanism

The equilibration of charges across the semiconductor|electrolyte interface is fundamental to the function of photoelectrodes and our understanding of them. The electron electrochemical potential of the semiconductor equilibrates with that of the electrolyte solution when they are placed in contact. The dark charge flow across the semiconductor|liquid interface induces a space-charge region in the semiconductor. As mentioned in Section 1.2, the band bending generates a thermodynamic driving force for photogenerated charges to move in opposite directions such that one kind of carrier accumulates at the surface while the other is pushed toward the bulk. The spatial charge separation is key to lengthening the charge carrier lifetimes and making interfacial redox reactions kinetically competitive with recombination.

Our understanding of PEC cells has been built upon knowledge of inorganic photoelectrodes as the charge carrier dynamics of photoelectrodes have been much more studied for inorganic materials, namely metal oxides, compared to organic materials.^47,278−281^ Being comparatively a recent development, it has not been unambiguously established whether key concepts derived from inorganic photoelectrodes can apply to organic photoelectrodes. For example, Section 4 shows that depositing a cocatalyst on the surface of photoelectrodes is important, both for inorganic and organic materials, to increase their activity. While there is some debate whether the activity improvement arises from passivation of trap states, enhancement of band bending,^282−284^ improvement of catalytic activity,^285,286^ or a combination thereof, it is clear that the semiconductor|cocatalyst interface is critical. Structurally ill-defined polymers like CN_*x*_ have a high density of defects^287^ and structural disorder can also lead to defect-rich organic materials.^288^ The defects are associated with localized electronic states, which may reduce the applicability of concepts such as band bending which are derived from a delocalized band structure. Notably, it has been realized that transport models derived from metal-oxide-semiconductor field-effect transistors (MOSFETs) could not accurately describe the behavior of organic field-effect transistors (OFETs).^289^ Trap states must be explicitly considered to develop models with good agreement. Ionic functionalities have also been shown to lead to doping of organic semiconductors and can influence the energy of electronic states at the interface, which would impact function.^290^

The level of understanding of OER and HER is much higher for inorganic materials than organic ones. Considering the OER, the inorganic core of the oxygen-evolving complex (OEC) in photosystem II has been intensely studied, more recently with cutting edge X-ray free-electron lasers.^291−293^ While there is still some debate whether the O–O bond is formed through a radical mechanism or a nucleophilic attack in the OEC,^294^ it is clear that the successive oxidation of Mn centers occurs in a relatively narrow electrochemical potential window. This has also been observed for inorganic hematite photoanodes and suggests some generality.^295^ A concept known as redox leveling is invoked, where the loss of electrons is coupled to the loss of protons to maintain a relatively constant redox potential for subsequent oxidations.^296^ The inorganic semiconductors used in photoanodes, like PSII, have redox-active metal centers with a range of possible oxidation states in close proximity of each other, which appear to determine the OER mechanism.^295^ It seems doubtful that such mechanisms apply to organic materials considering the more limited accessible oxidation states and that it is unclear whether neighboring repeat units could act cooperatively for catalysis. The limited *operando* studies to reveal the reaction intermediate formed in organic systems hinder a deep understanding.

**Figure 27:**
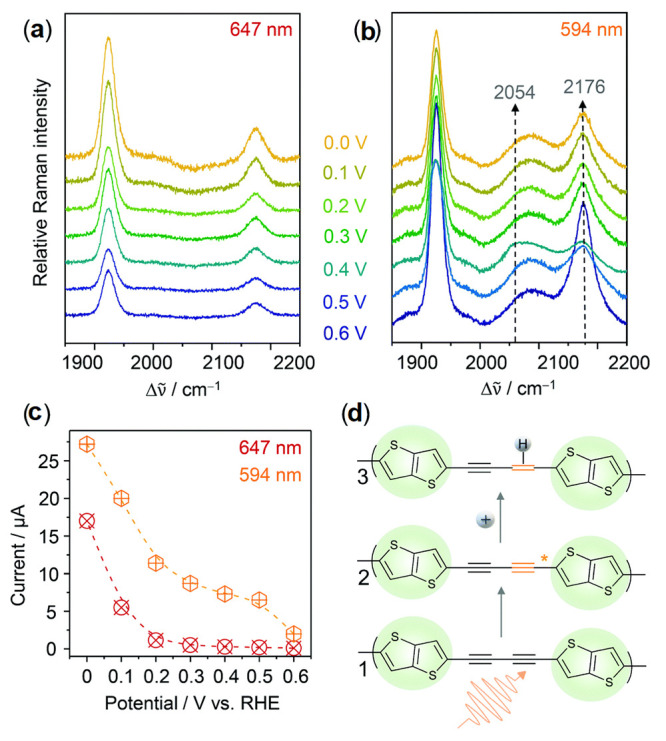
(a) EC-Raman spectra of pDET/Cu under 647 nm laser excitation. (b) EC-Raman spectra of pDET under 594 nm laser excitation. (c) Potential-dependent currents for HER as a function of excitation wavelength. (d) Photoelectrocatalytic reaction scheme for photoinduced hydrogen evolution. Adapted with permission from ref (308). Copyright 2021 Royal Society of Chemistry.

For PEC HER, the mechanism is not well understood for conjugated polymer systems compared to inorganic metallic electrodes. Model systems such as Pt and NiPt have been used to understand details such as the catalytic role of adsorbed hydroxyls and alkali metal cations,^297^ the change in the rate-determining step based on pH and the surface structure,^298^ and the water structure at the electrode|electrolyte interface.^299^ In contrast, the identification of active sites in organic polymers from where HER proceeds are not straightforward. Two main active sites have been proposed. The first is metal-centered catalytic active sites,^300,301^ dominant in conjugated polymers with metal centers, where HER is thought to occur through a homolytic or heterolytic path involving the formation of metal hydride intermediates.^302,303^ The second suggested active site is an excited state antibonding orbital with localized electrons, in the absence of metal.^304,305^ This follows recent experimental results by Sun et al. where poly(2,5-diethynylthieno[3,2-*b*]thiophene grown on copper support (pDET/Cu) displayed similar photocurrents (0.37 mA/cm^2^ at 0.3 V vs RHE) in both pristine electrolyte and one blended with 10 mM of thiocyanate (SCN^–^) ions,^304^ a widely known poisoner of metal-centered catalytic sites.^306,307^ This presents demonstrable evidence that the Cu species in pDET/Cu may not be the active site for PEC HER. Instead, the acetylenic units, which have excited states with a high electron density antibonding orbital, are more liable to act as highly active centers for HER. This assumption has been investigated via electrochemical-Raman (EC-Raman) spectroscopy.^308^ Laser lines above (594 nm) and below (647 nm) the bandgap of pDET (2.17 eV, equivalent to 571 nm) were chosen to understand the influence of the photoexcited states. The spectra obtained under 647 nm laser excitation presented bands at 1924 and 2174 cm^–1^ corresponding to the vibrations of neighboring acetylenic units (C≡C—C≡C) (Figure 27a). The potential dependent current plot under illumination at this wavelength showed an onset potential for HER at 0.2 V vs RHE (Figure 27c). On the other hand, additional bands at 2089 and 2054 cm^–1^ representing active and transient bands, respectively, were observed under sub-band gap 594 nm laser excitation (Figure 27b). The current onset potential was shifted to 0.5 V vs RHE (Figure 27c). The transient band appeared at a less positive potential (below 0.5 V) in place of the band at 2174 cm^–1^ indicative of triple bond vibration with lower bond strength. On the basis of these measurements, the acetylenic units were considered as active sites for hydrogen adsorption. As shown in Figure 27d, the acetylenic units are initially activated by the absorption of light. This then induces the attraction of more electron density into the C≡C causing attenuation in bond strength and observed as a shift to lower wavenumbers in the EC-Raman spectra. The role of thiophene units in these polymeric systems is limited to band gap tuning and therefore cannot act as active centers for HER since it is less susceptible to photoactivation as demonstrated by EC-Raman spectroscopy.^308^

**Figure 28:**
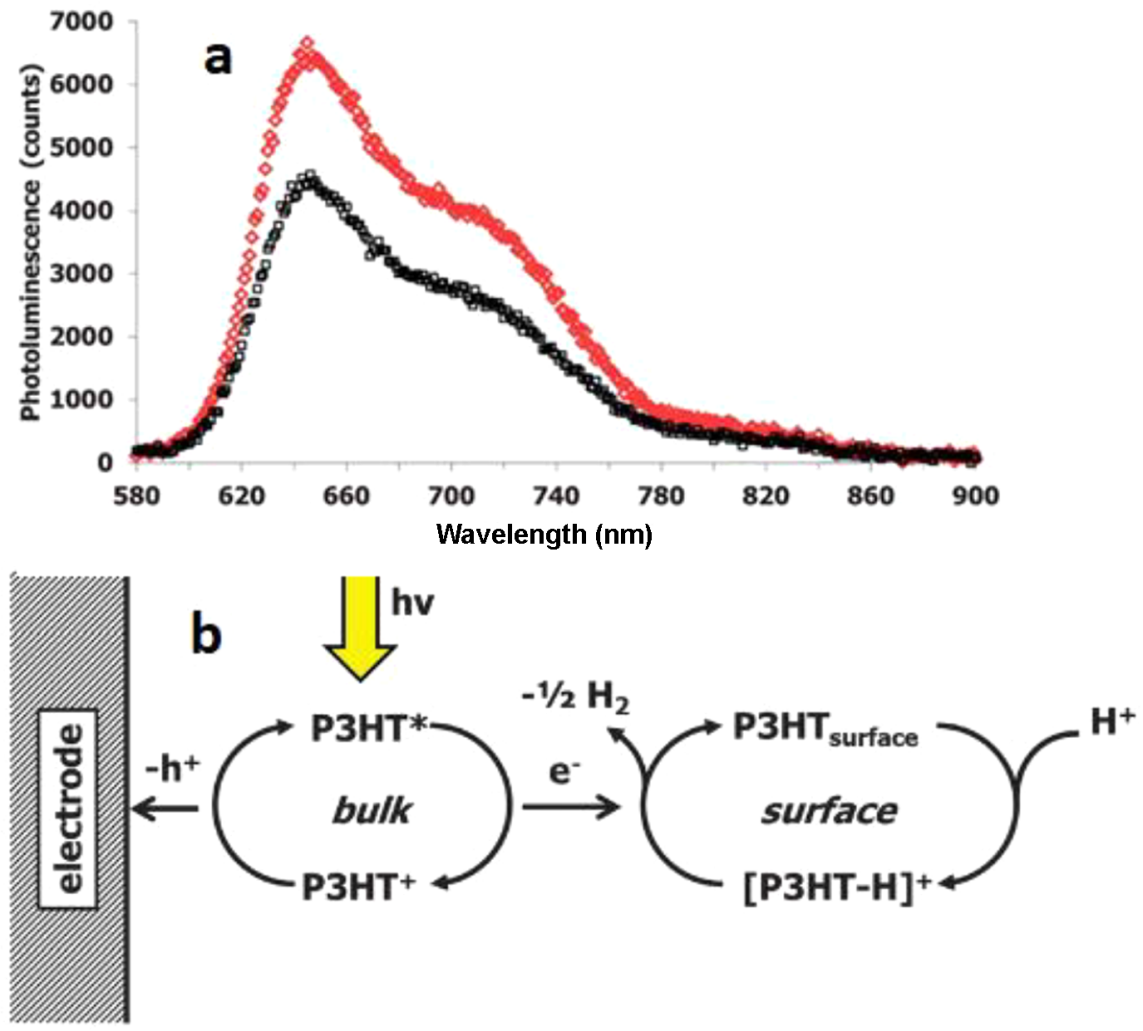
(a) Photoluminescence spectra of RR-P3HT films (a spectrum of the dry film in red and a spectrum of the film in contact with 0.1 M H_2_SO_4_ in black) at 550 nm excitation wavelength. (b) Proposed mechanism of RR-P3HT photocathodic activity in an aqueous solution. Adapted with permission from ref (143). Copyright 2013 Royal Society of Chemistry.

Suppes et al.^143^ have also investigated the mechanism of HER by employing a solution-processed regioregular RR-P3HT polymer (prepared over a Ni catalyst) as a photocathode. Given the involvement of hydrogen (as hydride or proton) in the intermediate step^309^ and the susceptibility of P3HT polymer to protonation in the presence of strong acid,^310^ the behavior of RR-P3HT in 0.1 M H_2_SO_4_ was monitored by PL experiments. The PL intensity of the RR-P3HT quenches upon exposure to the aqueous acid solution (Figure 28a) to an extent comparable with that for a pyridine-containing protonated pi-conjugated system.^311^ This together with the high tendency of exciton diffusion through the film^312^ indicates that protons may be involved in the interfacial charge transfer reaction. As such, the mechanism was proposed to proceed via an intermediate that involves the protonation of P3HT at the polymer|electrolyte interface, followed by the reception of photogenerated electrons from the bulk to release hydrogen. The initial interfacial acid–base reaction at the P3HT surface has been suggested to be the driving force for the transfer of photogenerated electrons from the bulk to the surface.^147^ When the protonated p-type P3HT polymer is brought in contact with an electrolyte solution, the Fermi level of the semiconductor equilibrates with the redox potential of the electrolyte by transferring holes (majority carriers) from the electrode to the electrolyte. As such, the Fermi level shifts to a more negative potential, and the redox potential in the Helmholtz layer of the electrolyte decreases until equilibrium is reached. At equilibrium, the reduction in the density of holes near the surface induces the formation of a space charge layer (SCL) with an electric field that gives rise to band bending. The separation of the excitons generated within the SCL under illumination is driven by the band bending, such that holes move away from the interface with the electrolyte and electrons move toward it. The model describes the transfer of electrons from the bulk to the surface (Figure 28b) although these regions were not defined concerning the SCL. It is unclear whether photogenerated electrons from outside the SCL can reach surface sites. If recombination outside the SCL outcompetes transport to the SCL and surface, which is expected based on the short lifetimes of excitons in the bulk, this implies that the structure of the polymer film should be matched to the SCL width. We also note the possibility that charge accumulation at the interface with the electrolyte breaks down charge neutrality within the polymer. In this case, the effective space charge is represented by a single layer within the bulk of the semiconductor, and charge separation could occur far from the surface.^313^ The mechanism proposes active sites on P3HT, though it should be noted that metal-centered active sites from residual Ni from the synthetic route in P3HT cannot be discounted.^314,315^

The following will discuss the key factors affecting the charge dynamics of polymer photoelectrodes that have been explored, namely junction formation, porosity and surface area, and crystallinity.

### 5.2 Architectural Modifications and the Charge Carrier Dynamics

#### 5.2.1 Junctions

Considering inorganic semiconductors, a high dielectric constant reduces the exciton binding energy that holds the electron and hole together as a pair to levels comparable to the available thermal energy at room temperature. The excitons will thus spontaneously dissociate, and exciton dynamics typically are not relevant under standard operating conditions. In contrast, organic semiconductors have a low dielectric constant which increases the exciton binding energies to an extent greater than the thermal energy.^316^ The excitons persist at room temperature, and their dynamics will impact device function. Similar to BHJ photovoltaics, a blend of separate donor and acceptor polymers can be adopted in polymer-based organic photoelectrodes to generate free charges.^317^ Another approach is differential doping of a material to form a homojunction, such as in bulk homojunction PV^318^ and typical silicon PV.^319^ This strategy can also be applied to polymer-based organic photoelectrodes to promote the generation of free charges. Junctions (homojunctions and heterojunctions) have thus been actively pursued in the design of more efficient organic PEC cells.

**Figure 29:**
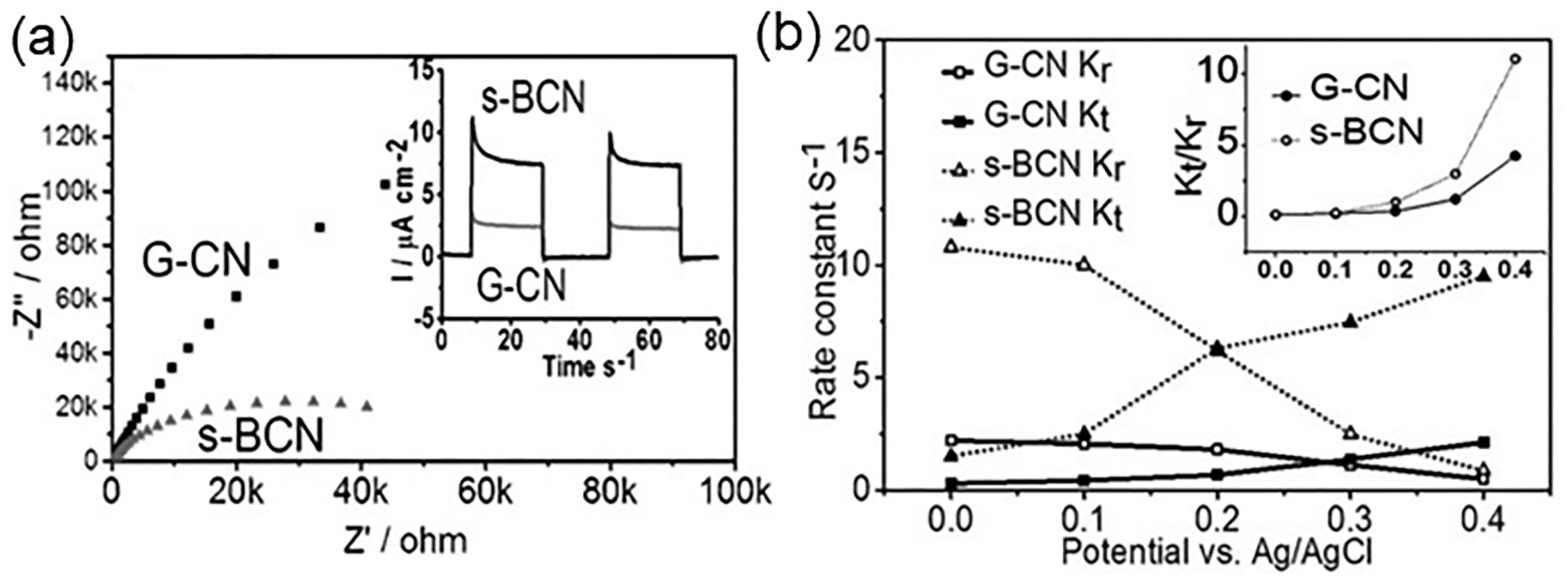
(a) Nyquist plots of G-CN and s-BCN obtained by applying a sine wave with amplitude of 5.0 mV over the frequency range from 10 kHz to 0.1 Hz. Inset: Periodic on/off photocurrent response of G-CN and s-BCN electrodes in 0.1m Na_2_SO_4_ with 0 V bias versus Ag/AgCl. (b) Potential dependence of the rate constant *k*_t_ and *k*_r_ for s-BCN and G-CN samples. Illumination: 365 nm UV light. Adapted with permission from ref (104). Copyright 2017 Wiley-VCH.

In 2017, Ruan et al.^104^ investigated the influence of a nanojunction between bulk carbon nitride (G-CN) and a B-doped CN nanolayer on the photoanodic performance of the photoelectrode. They used electrochemical impedance spectroscopy (EIS) to establish that the formation of bilayer homojunction (s-BCN) dictates the separation of charge carriers in the photoanode. s-BCN photoanode produced a photocurrent density of 0.103 mA/cm^2^ at 1.23 V vs RHE in 0.1 M Na_2_SO_4_ under one sun irradiation. This outperformed the bare G-CN photoanode which showed a photocurrent density of 0.0106 mA/cm^2^ at 1.23 V vs RHE. The IPCE at 400 nm was nearly 10% for s-BCN compared to 1% for CN. The improved water oxidation by s-BCN was attributed to the bilayer heterojunction which improved charge transfer to the electrolyte. The EIS Nyquist plots in Figure 29a showed a decrease in the semicircle diameter for s-BCN compared to G-CN, which translates to a 3-fold increase in electron transfer conductivity.

A more refined kinetics analysis was provided by intensity-modulated photocurrent spectroscopy (IMPS). The kinetics of charges are represented by the first-order rate constant of surface recombination (*k*_r_) and interfacial charge transfer (*k*_t_). This assumption may not hold for the multielectron, multiproton water oxidation reaction. However, similar analyses have been performed to elucidate the rate constants of the competing productive charge transfer and unproductive charge recombination processes for inorganic photocatalytic systems.^320−323^ As shown in Figure 29b, *k*_r_ decreases with increasing potential, typical for an ideal semiconductor|electrolyte interface. This is attributed to the stronger band bending caused by the more positive applied bias, enhancing charge separation, and suppressing charge recombination at the surface. Additionally, at low potential, *k*_r_ in G-CN is lower than in s-BCN due to the introduction of surface defects during B doping which acts as charge recombination centers. In effect, a high concentration of dopants may promote surface recombination and compromise PEC performance.^324,325^ For photoelectrodes based on CN_*x*_, doping past the optimal level mostly introduces trap states which serve as recombination centers irrespective of the redox reaction being monitored.^101^ At high potentials, dopant-induced surface recombination can be mitigated by the applied bias which directs electrons away from the surface and to the counter electrode.^104^ As seen in Figure 29b, *k*_t_ for s-BCN at 0.2 V vs Ag/AgCl was approximately 10 times larger than that of G-CN. Also, at 0.4 V vs Ag/AgCl and under 365 nm UV light irradiation, the ratio of *k*_t_ to *k*_r_ was larger in s-BCN than in G-CN. The authors, therefore, concluded that more charges were available for oxygen evolution in s-BCN than in the bulk G-CN because of the nanojunction formation. This can be understood by considering the energetics of the system. The B-doping shifts the valence band of the CN_*x*_ upward and generates a driving force for photoexcited holes to transfer from G-CN to the B-doped CN_*x*_ layer. Electrons can flow in the opposite direction under a weak bias. The opposite movement of photogenerated charges induces charge separation which dramatically improves the photoanode performance.

Functionalization with heteroatoms was also attempted to improve the performance of a CN_*x*_ photoelectrode. Fang et al.^91^ investigated this option by blending S-containing compounds into non-S precursors in the weight ratio of 1:1, 1:2, 1:5, and 1:10, in the synthesis of CN_*x*_ films. The S acted as a directing agent^326^ for the growth of the polymeric CN_*x*_ films on FTO as well as connections to assist charge migration. A photoanodic performance of 0.100 mA/cm^2^ was recorded at 1.23 V vs RHE under AM 1.5 illumination in a NaOH electrolyte solution. This compared favorably to the nondoped CN_*x*_ (0.008 mA/cm^2^) and was accredited to the limited defects along with the interfaces and the reduced charge recombination associated with the heterojunction formation.^327,328^ The maximized photoanodic performance was achieved with films synthesized from a 1:2 weight ratio of S:non-S-containing compounds. This was consistent with the EIS Nyquist plot in which the polymeric CN_*x*_ (PCN_*x*_) films made from the 1:2 weight ratio presented the smallest diameter compared with the other weight ratios, signifying efficient charge transport. Also, the optimized system exhibited efficient charge separation which was characterized by the dramatic decrease in emission peak at 470 nm compared to the high emission intensity observed for the other samples. This is an indication that radiative recombination was minimized as a result of exciton dissociation.^329^

To better understand the role of heterojunctions, Shalom and co-workers studied the charge recombination process between CN_*x*_ and mesoporous TiO_2_ using time-resolved photoluminescence (trPL) and TAS.^330^ They reported the electron injection rate from the CN_*x*_ excited states to the TiO_2_ conduction band by steady-state and trPL and the hole extraction kinetics using various liquid electrolytes and solid-state hole conductors. They observed that the emission spectrum of CN_*x*_ on glass displayed a wide band centered at 510 nm. The emission was drastically quenched when CN_*x*_ was deposited on TiO_2_ to create a heterojunction, indicative of CN_*x*_ excited state quenching. The magnitude of the emission quenching of the CN_*x*_/TiO_2_ heterojunction film compared with that of CN_*x*_ on glass was used to calculate an electron injection yield of about 90%. The emission of CN_*x*_/TiO_2_ also decayed faster than that of CN_*x*_ alone, consistent with electron transfer. The recombination processes of CN_*x*_/TiO_2_ and CN_*x*_ on glass were studied by TAS. The transient spectrum of CN_*x*_/TiO_2_ at 50 μs after excitation at 532 nm showed a photoinduced absorption band from 600 nm increasing toward the near-IR (NIR) spectral region. In contrast, the transient spectrum of CN_*x*_, the reference material, showed a negligible signal, consistent with the typical absorption onset of <450 nm for CN_*x*_, indicating that transient signals originated from the photoinduced electron transfer and not the deactivation of the CN_*x*_ excited states. The ground state absorption of the CN_*x*_/TiO_2_ samples extended into the NIR range, suggesting that the interface plays a role in modifying the electronic structure of CN_*x*_. Also, the hole transfer process between the excited states in CN_*x*_ and different hole acceptors was studied. Notably, P3HT coated on CN_*x*_/TiO_2_ films showed an enhanced TAS signal in the region from 750 to 925 nm.^330^ This signal was due to the charge transfer complementarity that occurred from the P3HT to TiO_2_ and CN_*x*_ since P3HT coated on CN_*x*_/glass showed no signals. The spectrum for P3HT/CN_*x*_/TiO_2_ was attributed to the individual contributions from the oxidized CN_*x*_ and P3HT formed by electron injection into the CB of TiO_2_.

Decorating the surfaces of organic photoelectrodes with cocatalysts presents a promising approach toward improving performance. For example, Fan et al.^331^ showed the incorporation of a layered double hydroxide (LDH) onto the structure of CN_*x*_. In their work, CN_*x*_ was grown on an FTO substrate and the NiCo-LDH was electrochemically deposited on top. The resulting CN_*x*_/NiCo-LDH composite generated a photocurrent of 0.0118 mA/cm^2^ at 0.6 V vs SCE, representing an increase of almost 3 orders of magnitude compared to bare CN_*x*_. The EIS data demonstrated that the bare CN_*x*_ film had the highest charge resistance. The charge transfer initially decreased when loading with NiCo-LHD up to 10 mC of charge passed. Beyond this optimal cocatalyst loading, the charge transfer resistance increased. This may be due to the formation of surface recombination sites at high amounts of additives, as we discussed above.

#### 5.2.2 Porosity

**Figure 30:**
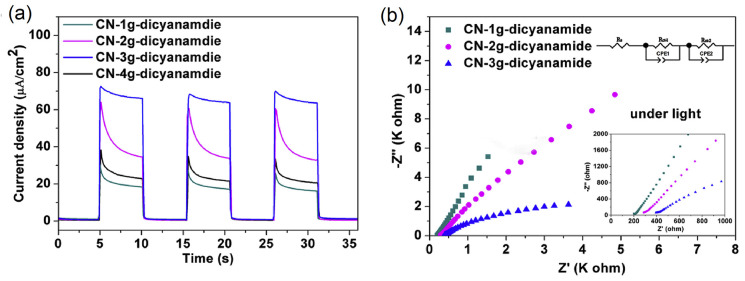
(a) Transient photocurrent density and (b) EIS spectra measured at 1.23 V versus RHE under light illumination of the CN films prepared using different amounts of dicyanamide. Adapted with permission from ref (87). Copyright 2017 Elsevier.

The growth of porous organic materials with considerably large surface area for PEC OER and HER has also been investigated. If exciton dissociation (free charges generation) can occur at the semiconductor|electrolyte interface, then increasing the surface area could potentially result in maximizing the photocurrent, and the conventional donor:acceptor type heterojunction would not be needed to generate charges. However, this may not apply to all systems as other studies^287,332,333^ have shown that charge generation could proceed on faster time scales as opposed to exciton diffusion to the interface. Polymeric CN_*x*_ electrodes with high porosity have recently been prepared for solar water splitting.^87,97^ Lv et al.^87^ reported a photoanode based on CN_*x*_ synthesized from cyanamide, melamine, or dicyandiamide (DCDA; note that the authors used the incorrect name dicyanamide) by a two-step vapor deposition. The obtained CN_*x*_ films displayed uniform morphologies with the DCDA-based film possessing the highest surface area, as a result of its smaller particle size. Under 1 sun illumination, the optimized CN-3g-dycyanamide films gave rise to a stable anodic photocurrent of 0.063 mA/cm^2^ at 1.23 V vs RHE (at pH 7) with an onset potential of 0.41 V vs RHE and a maximum IPCE of 6.6% at 350 nm. The stable anodic photocurrent delivered, implied that generation, separation, transport, and recombination of the charge carriers attained equilibrium as shown in the transient current curves of Figure 30a. Compared with melamine and cyanamide monomers, the superior PEC performance of DCDA-based CNx films was linked to enhanced light absorption and improved charge transfer at the photocatalyst|electrolyte interface as a result of a large surface area. The lower charge resistance at the photocatalyst|electrolyte interface of the optimized film (Figure 30b) is in line with its higher photoactivity.

**Figure 31:**
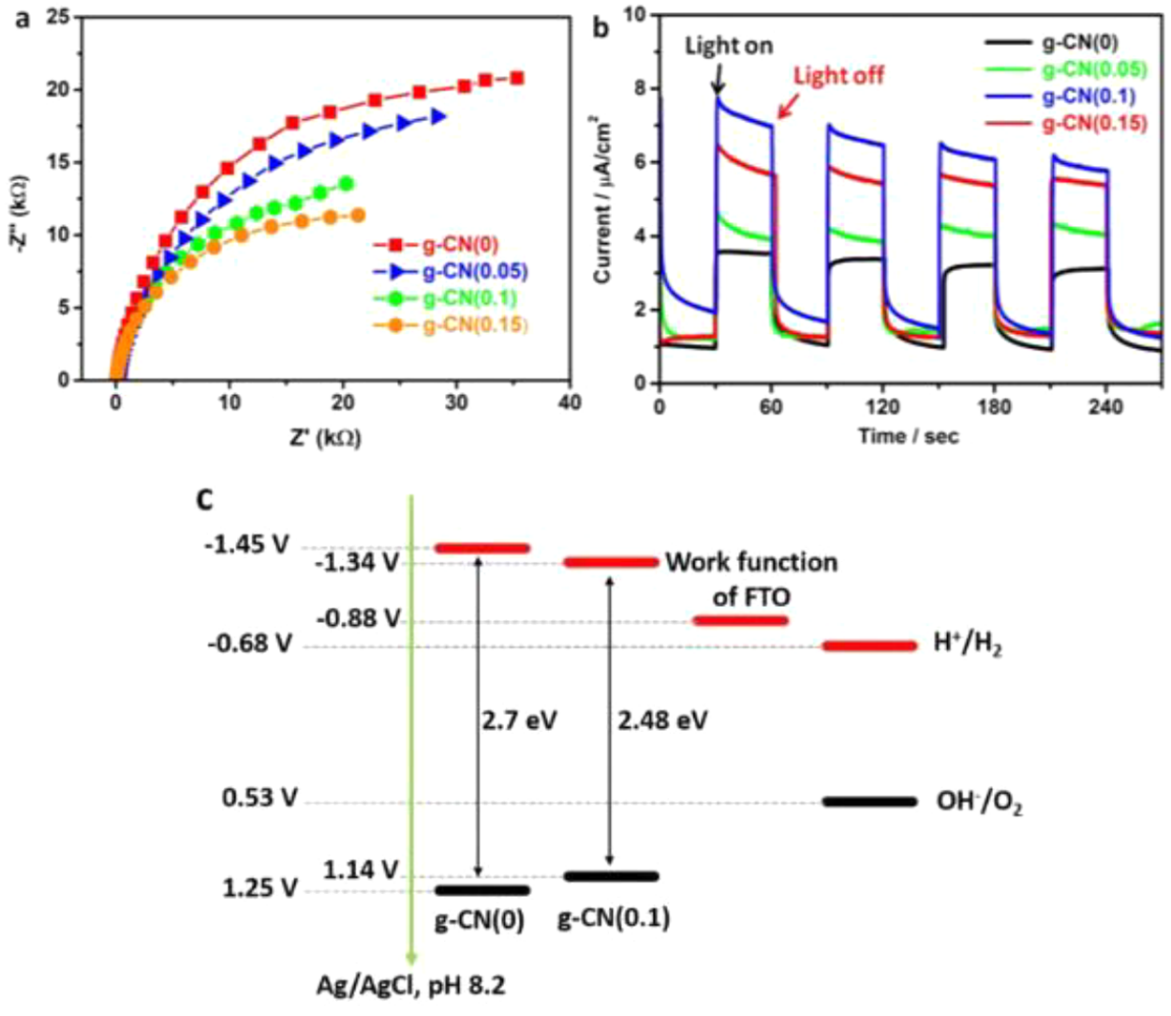
(a) Nyquist plot of the CN films at 1.23 V vs RHE in dark condition. (b) Photocurrent of the CN films at 1.23 V vs RHE in 0.1 M KOH aqueous solution under one sun. (c) Energy diagram of the g-CN(0)/g-CN(0.1)/FTO junction. Adapted with permission from ref (96). Copyright 2018 Wiley-VCH.

A relatively simple pathway for growing highly porous and large-scale CN_*x*_ films with controllable chemical and photophysical properties has recently been reported by Peng et al.^96^ Employing the doctor-blade technique, CN_*x*_ films were grown on a FTO substrate using a supramolecular paste containing typical CN_*x*_ precursors and barbituric acid. The resulting uniform and transparent films with a large surface area were presented as CN(*x*), *x* = 0, 0.05, 0.1, and 0.15 concerning the mass of barbituric acid used as a source of carbon doping. The advantage of high porosity and increased surface area was demonstrated by the increase in photocurrent from 0.0033 mA/cm^2^ to 0.0075 mA/cm^2^ at 1.23 V vs RHE (in 0.1 M KOH) between the less-porous (g-CN(0)) and more porous (g-CN(0.1)) films (Figure 31b). It was anticipated that increasing the C-doping would correspond to higher electronic conduction and effective charge transfer (Figure 31a). Past the optimal doping of *x* = 0.1, the performance decreases as a result of enhanced recombination induced by the excess carbon sites. Heterojunctions could also be prepared by the doctor-blade technique employed. The g-CN(0)/g-CN(0.1) heterojunction is thought to facilitate charge separation despite the type-I heterojunction (Figure 31c): both electrons and holes would transfer to g-CN(0.1) if only considering the energetics. This is characterized by a strong fluorescence quenching indicative of reduced recombination.

#### 5.2.3 Crystallinity

**Figure 32:**
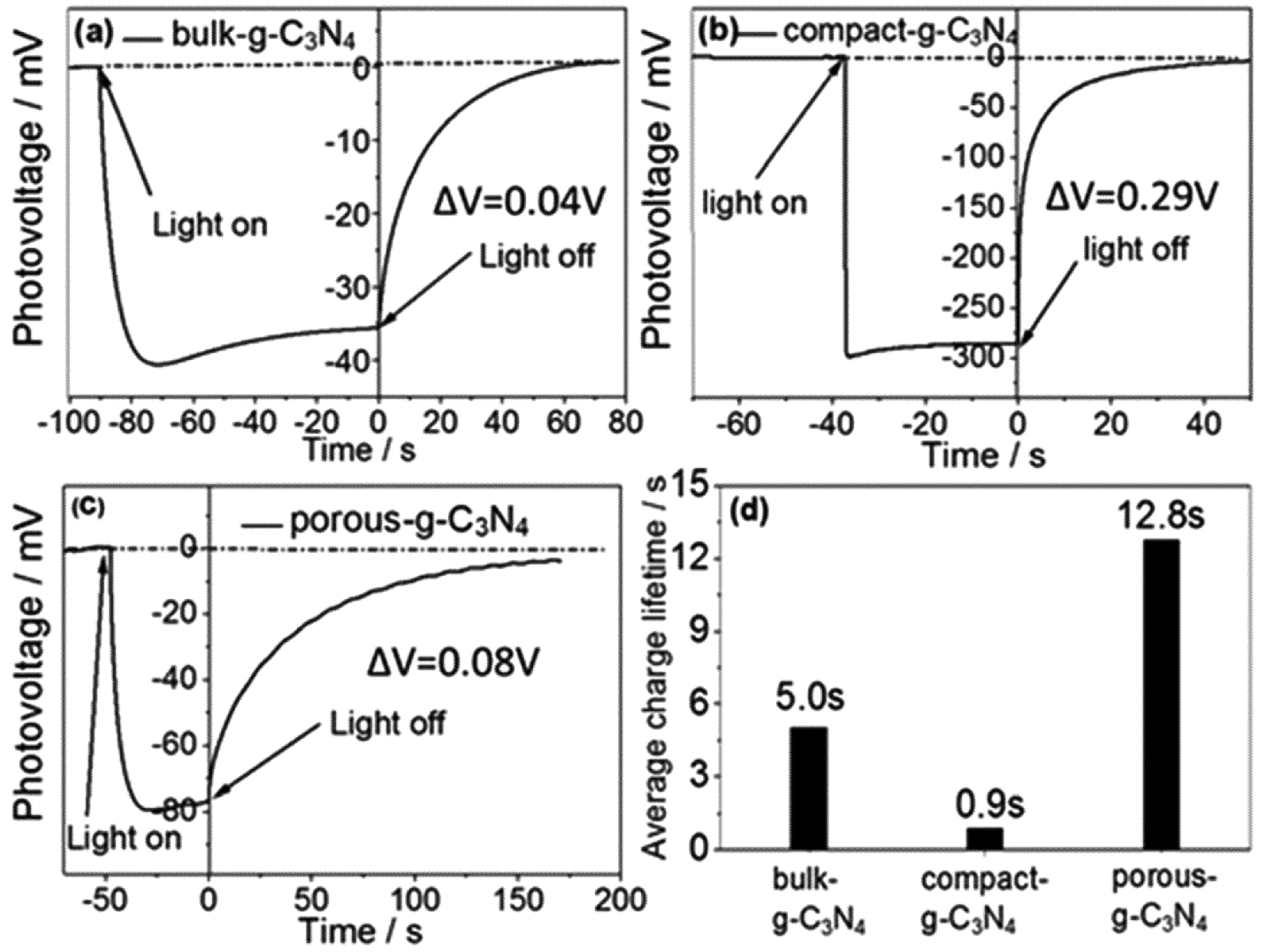
Open circuit voltage decay (OCVD) plots of (a) bulk CN_*x*_, (b) compact CN_*x*_, and (c) porous CN_*x*_ with 150 W xenon lamp illumination from the electrolyte–electrode (EE) side. (d) Calculated average charge lifetimes in the g-C_3_N_4_ films. (Generated photovoltage Δ*V* is the difference in voltage between dark and illumination conditions). Adapted with permission from ref (94). Copyright 2019 Royal Society of Chemistry.

A final design strategy that has attracted the interest of researchers with tangible prospects in facilitating charge transfer in organic photoelectrodes is crystallinity. Ruan et al.^94^ reported the effect of crystallinity on the PEC performance of CN_*x*_ films. Their findings established that long-lived charge carriers reside in more poorly crystalline samples, due to deeper trap states. To explore the impact of trap states, bulk CN_*x*_, porous CN_*x*_, and compact CN_*x*_ films were fabricated, representing moderate crystallinity, low crystallinity, and high crystallinity, respectively. Crystallinity was assessed from the full width at half-maximum (fwhm) values of the 110 XRD peak at 27.5° (1.0°, 1.1°, and 0.7° for bulk, porous, and compact, respectively) where lower fwhm values represent higher crystallinity.^94,334,335^ Open circuit voltage decay (OCVD) measurements were used to better understand the role of trap states. The average charge lifetime was determined by fitting a biexponential function to the decay curves (Figure 32). Photovoltage decay in the compact CN_*x*_ film (Figure 32b) was much faster than bulk (Figure 32a) and porous (Figure 32c) samples. The average electron lifetimes were found to be 0.9 s for compact, 5.0 s for bulk, and 12.8 s for the porous sample (Figure 32d). The short average lifetime indicated rapid charge recombination in the absence of electron donor, while the longer electron lifetimes were mainly attributable to the severe electron trap effect. The long-lived electrons in the bulk and porous samples are mostly trapped at deep levels and located at low energy levels, hence, incapable of participating in redox reactions.^287^ In effect, the migration of electrons in the photoelectrode was hindered by deep trap states, and reducing the deep trap state density promotes charge transfer efficiency and boosts photocurrent density. The lower trap state density in compact CN_*x*_ films enabled more charges to reach the interface and participate in redox reactions. This is also supported by the large photovoltages recorded.

**Figure 33:**
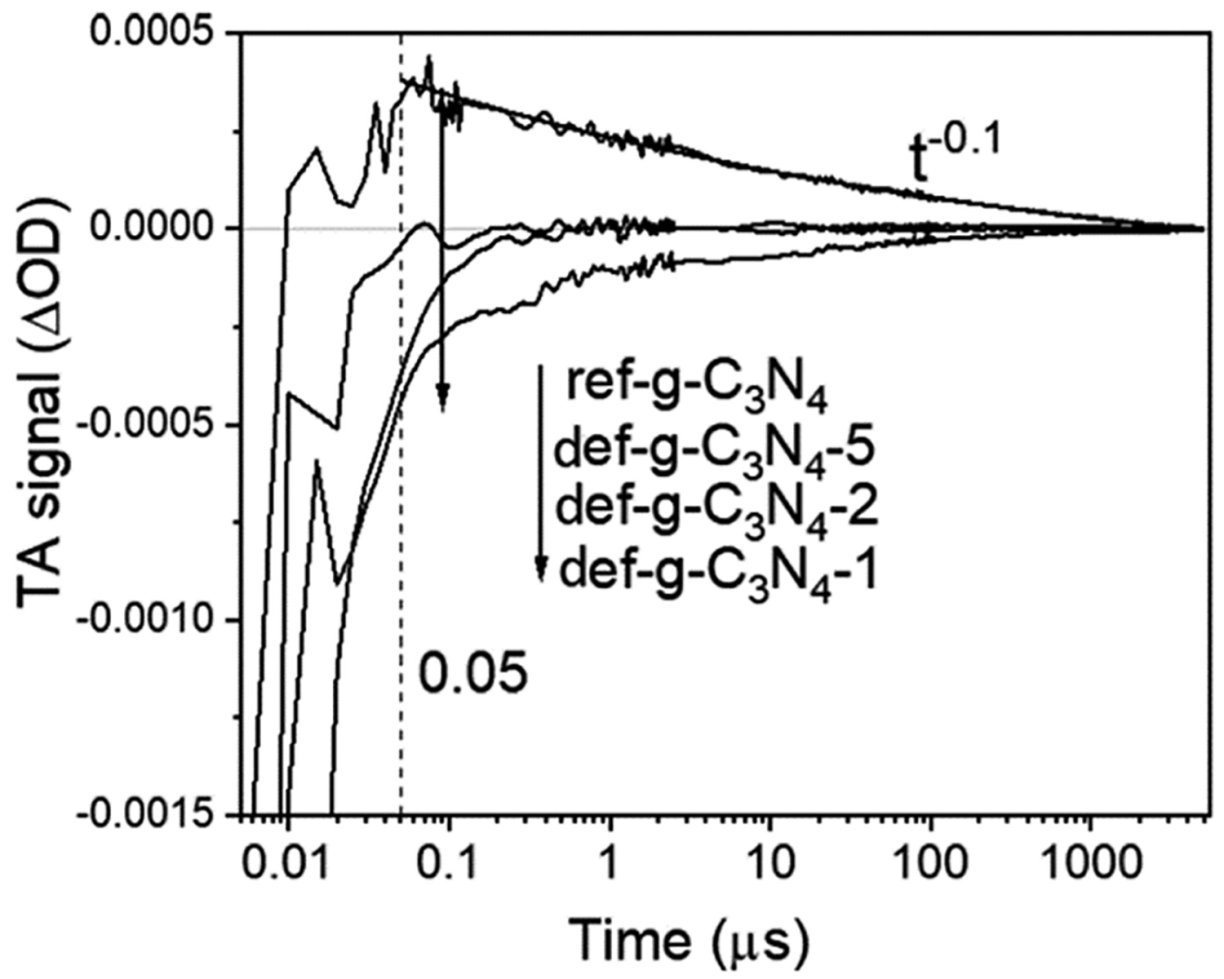
TAS kinetics of ref-*g*-C_3_N_4_, def-*g*-C_3_N_4_-1, def-*g*-C_3_N_4_-2, and def-*g*-C_3_N_4_-5 samples under N_2_ atmosphere after 355 nm excitation (200 Hz, 850 μJ/cm^2^/pulse), monitored with a 660 nm probe. Adapted from ref (122). Copyright 2020 American Chemical Society.

Recently, an engineered CN_*x*_ photocathode with a considerable amount of trap states as a result of N-defects and C–OH terminal groups has been presented by Ruan et al.^122^ The material showed improved properties compared to pristine CN_*x*_ with respect to conductivity and the lifetime of shallow-trapped charges. Quantitatively, conductivity increased by 2 orders of magnitude, and the lifetime of the shallow-trapped charges increased by 3 orders of magnitude in the optimized def-*g*-C_3_N_4_ compared with ref-*g*-C_3_N_4_. The average electron lifetime increased from 0.9 to 5.5 s as trap states were introduced, in line with a reduction in charge carrier mobility associated with charge trapping in defect-rich samples. In the absence of trap states, the decay kinetics of CN_*x*_ after about 50 ns of photoexcitation may be described by a power-law function. Nonetheless, this description may not hold for decay kinetics that precedes 50 ns due to the influence of emission. As shown in Figure 33, the negative TAS signal observed for def-*g*-C_3_N_4_ is significant up to tens of microseconds whereas the electron lifetime in the shallow emissive state of ref-*g*-C_3_N_4_ is less than 50 ns.

**Figure 34:**
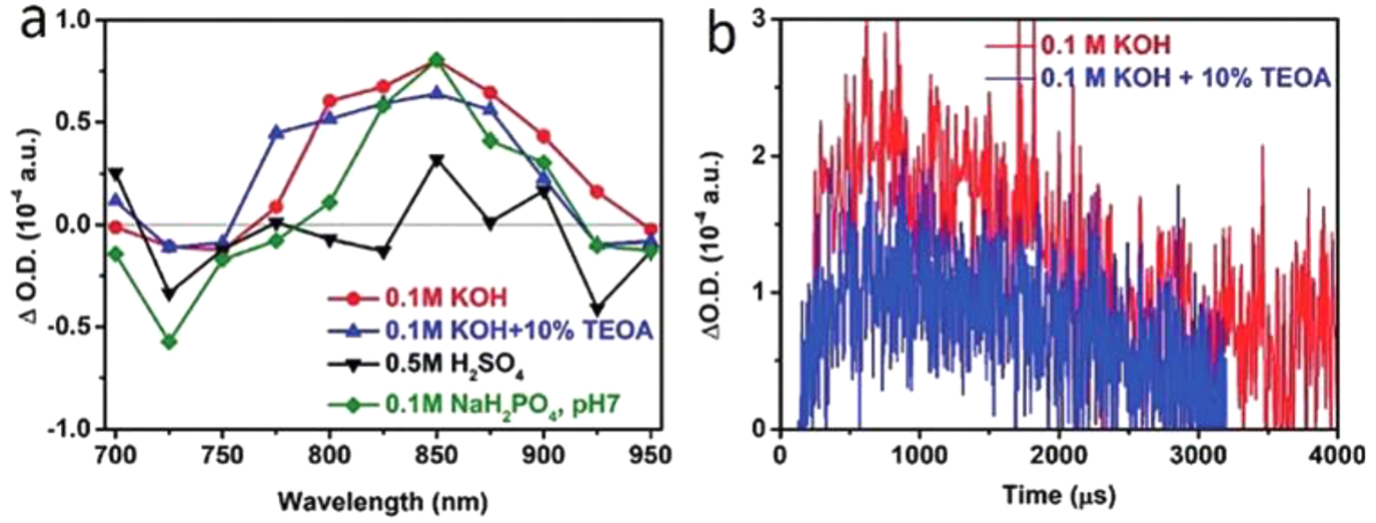
(a) TAS spectra (delay time unspecified) of a CN_*x*_ film soaked in different aqueous solutions. (b) TAS decay of a CN_*x*_ film soaked in 0.1 M KOH, and 0.1 M KOH containing a 10% TEOA aqueous solution monitored at 850 nm. Adapted with permission from ref (95). Copyright 2018 Wiley-VCH.

Similarly, Shalom and co-workers reported a simple method to grow a densely packed CN_*x*_ film by crystallization of CN_*x*_ monomers on a FTO substrate, followed by thermal condensation.^95^ The photoanode prepared from melamine delivered a photocurrent of 0.116 mA/cm^2^ at 1.23 V vs RHE and up to a 1 V shift of the onset potential under one sun in 0.1 M KOH (pH = 13) aqueous solution with IPCE of 8.5% at 400 nm. However, the photocurrent decreases to 0.071 mA/cm^2^ in a neutral electrolyte (NaH_2_PO_4_, pH = 7) and further reduces to 0.064 mA/cm^2^ in an acidic electrolyte (H_2_SO_4_, pH = 0.2) at an onset potential below 0.3 V vs RHE. To elucidate the charge separation mechanism, electron lifetime, and hole extraction kinetics, TAS measurements of the CN_*x*_ film in various electrolytes at 1.23 V vs RHE were taken (Figure 34a). As could be expected from the increased photocurrents, the electron lifetime increases from 0.73 ms in an acidic electrolyte to 1.09 ms in a basic electrolyte. This suggests that the photoanode response displays progressive enhancement with increasing pH, similar to the behavior reported for hematite photoanodes.^336,337^ Generally, the surface charge density of semiconductors increases as pH decreases, and this affects the energetics of the semiconductor. As charges accumulate at the surface of the semiconductor, the Fermi level of the semiconductor equilibrates with these surface states instead of the redox couple of the solution, which reduces the extent of band bending^338^ and hence leads to poor charge separation. This may account for why photoanodic performance was maximized in the presence of the basic electrolyte and declined in the acidic electrolyte. As observed in Figure 34b, the presence of triethanolamine (TEOA) further increased the electron lifetime from about 1.09 to 2.26 ms because of a faster hole extraction, which suppressed electron–hole recombination. As a result, the photocurrent of the CN_*x*_ photoelectrode doubled to 0.245 mA/cm^2^ following the addition of 10% TEOA into the 0.1 M KOH.

An insight into carbon-based conjugated systems in relation to their catalytic activity is that the rate of exposure of the active sites on the catalyst is paramount, and it is linked to the dimensional state of the molecule. Higher-dimensional state systems would have limited exposure of their active sites/species as opposed to systems of lower-dimensional states.^338^ The easy manipulation of organic systems from one-dimensional state to the other via the introduction of new linker groups^339^ suggests the relevance of this to the catalysis community. The activity of substrate-bound polymer photoelectrodes is usually influenced by the interaction between the substrate and the photoelectrode. A strong combination between polymer photoelectrodes and substrates, which is achieved through irreversible chemical interactions, opposes resistance and promotes charge transportation at higher levels than those which are loosely bound via weak van der Waals forces.^120^ Efforts should therefore be directed at techniques that strongly bind photoelectrodes to their substrates to enhance PEC performance.

One question that remains unanswered despite the relevant information provided in this section is how material design strategies affect the behavior of charge carriers and their impact on device performance. In other words, the requisite information needed to fill the gap in our current understanding of the complexity of the charge carrier dynamics in relation to device performance is still lacking. The limited *operando* studies of organic-based photoelectrodes are immediately thought of as the main contributing factor to our present lack of understanding. Spectroscopic and electrochemical measurements that concurrently monitor key processes of charge separation, charge transfer, and recombination alongside the photocurrent density of organic-based photoelectrodes have been rarely reported. There are not enough studies that use techniques such as trPL and TAS to afford us information on these key processes and provide guidance for material and system optimization. Interestingly, of the material design strategies of junction formation, porosity, and crystallinity, only the charge carrier dynamics of junctions were characterized by both trPL and TAS measurements. Neither of these techniques was used to describe the charge carrier dynamics of porous systems. Only TAS was utilized to establish that in crystalline CN_*x*_ photoanode charge separation is enhanced, whereas electron–hole recombination declines as pH increases, leading to an increase in photoanodic performance.^95^ Most of the measurements performed on polymer photoelectrodes to describe the behavior of charges were found to employ electrochemical-based techniques. However, analyses of these measurements to give information on charge recombination kinetics other than charge transfer kinetics are yet to be reported. For instance, EIS measurement was utilized to establish that charge transfer at the photocatalyst|electrolyte interface of a porous photoelectrode material proceeds at a faster rate compared with its nonporous counterpart.^87^ In contrast, the recombination kinetics of these charges was not reported. Expanded analyses of EIS responses and other modulated electrooptical techniques applied to organic photoelectrodes will greatly improve our understanding.^104,320,323,340^ Additionally, coupling two or more spectroscopic techniques to explore and monitor the excited state structural and carrier populations, alongside the different photochemical/photophysical processes within these polymer photoelectrodes will provide rich information that will improve our understanding of their inner workings. For instance, performing integrated time-resolved Raman and TAS measurements on polymer photoelectrodes will afford us information on the excited state structural dynamics^341^ and charge carrier dynamics, respectively, which are key considerations for the architecture of next-generation efficient photoelectrodes.

## 6 Theoretical Modeling on Organic/Polymer-Based Semiconductors

The organic semiconductor materials used in polymer-based photoelectrodes are significant members of the family of functional materials, with great performance which sometimes supersedes that of their inorganic counterparts. However, the best performances of these organic materials are usually subject to addressing certain challenges at the atomic, molecular, and morphological length scales. This presents a daunting but exciting task to the computational community, including chemists, physicists, materials, and data scientists. In view of this, various methods and models have been developed with wide applications in the potential quantification and rationalization of the electronic properties of organic materials.

Generally, the interplay between the chemistry of the molecular core and the intermolecular factors of organic semiconductor materials dictate their performance and key electronic properties.^342^ These properties are manipulative and, hence, serve as an inspiration to both experimentalists and theorists. The common pitfalls encountered by organic and polymer semiconductors are their instability and lower charge mobility, which are influenced by either chemical or physical factors. Thus, the advanced understanding of the chemical and physical factors that determine the properties and performance of organic and polymer-based semiconductors is crucial toward improving their stability.^342^

For many π-conjugated polymer-based semiconductors, the magnitude of their charge transport properties is a function of the strength of the electronic coupling between adjacent cores, which has been shown to be consistent with the exchange component of the total interaction energy.^343,344^ As such, the fundamental challenges that have been identified in this arena are (1) the lack of universal theory of the charge transport and (2) the role of material’s morphology on the charge transport.^345−347^ Models of inorganic crystalline and amorphous solids have been used to discuss the charge carrier dynamics in organic semiconductors in the past. However, several limitations arise in the use of these models, particularly due to the differences in interaction between nuclear and electronic degrees of freedom that exist in both organic and inorganic solids.^345^ In response, newly developed methods for modeling charge transport in organic and polymer-based semiconductor materials turn to consolidate conventional quantum chemical methods and traditional methods used in soft-matter modeling.^345^ These methods are phenomenological theories, which are based on a simplified model of transport. Then a correlation between device response (e.g., current, mobility, voltage, or electric field) and parameters that can be used in the context of describing experimental results is deduced. The applicability of soft-matter modeling methods to organic semiconductor emanates from the fact that crystalline organic solids, held together by van der Waals forces are relatively soft and this softness impacts the charge transport. This modeling strategy is mostly used for organic electronics such as liquid crystals (LCs)^348^ and hence will not be critically looked at in this review.

**Figure 35:**
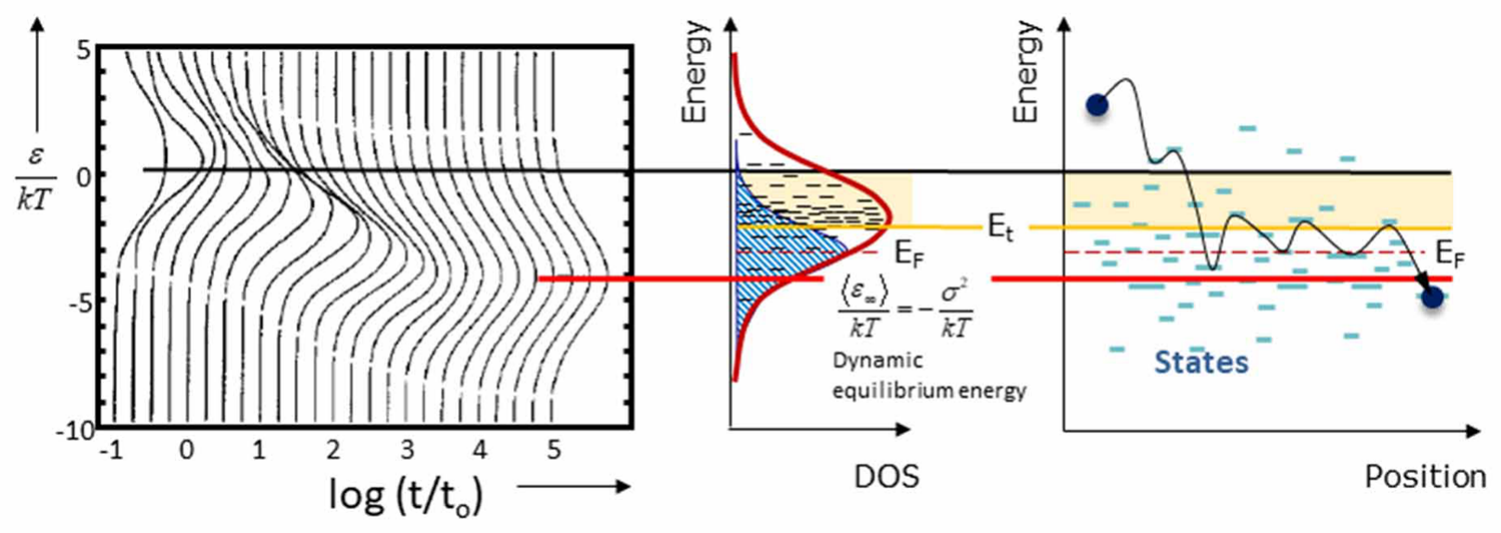
Relaxation of an injected charge carrier as a function of time in Gaussian DOS (left). Adapted with permission from ref (351). Copyright 1993 Wiley-VCH. Hopping transport of injected charge carrier via the states at thermal energy in Gaussian DOS (middle) and space (right). Adapted with permission from ref (354). Copyright 2021 IOP Publishing, Ltd.

The Gaussian disorder model (GDM) and its recent improvements^349^ and the multiple trapping model^350^ have emerged as the widely used models for describing the charge transport in polymeric semiconductors. The GDM describes the hopping transport across a disordered single or multiple component organic solid where charge transporting elements, which can either be the molecules participating in transport or segments of a main chain polymer that are separated by topological defects, are identified as sites whose energies of their hole or electron transporting states are subject to a Gaussian distribution of energies.^351^ This model has been developed from numerical simulations based on Monte Carlo (MC) simulations to an analytical approach.^352^ GDM based on numerical simulations has revealed that there is the likelihood of charge carrier hopping within the Gaussian DOS of a diluted system to accomplish a random walk subsequent to a relaxation to a dynamic equilibrium energy below the center of DOS (Figure 35). From the analytical approach in the GDM framework, this hopping is initiated from the energy levels above the transport energy (*E*_t_) and proceeds downward to spatially neighboring sites with lower energies under quasi-equilibrium conditions as displayed in Figure 35. However, a hop to a site below *E*_t_ is followed by an exponentially faster upward hopping mediated by thermal activation in the vicinity of *E*_t_ with a sequential hop to deeper energy levels below *E*_t_.^353^

**Figure 36:**
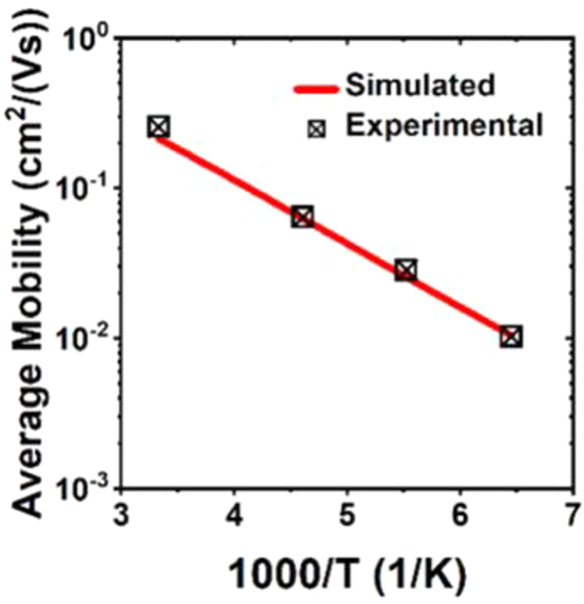
Simulated and experimental average mobilities of the diketopyrrolopyrrole-naphthalene copolymer (PDPP-TNT) for various temperatures. Adapted with permission from^359^. Copyright 2020 American Institute of Physics.

The multiple trapping model includes a mobility edge (ME) energy with an exponential density of shallow traps extending to lower energies.^350^ The ME model adopts the principle that there is a defined energy (the ME) in the density of states (DOS) that segregates mobile states from localized states. This suggests that immobile trapped carriers are temporarily made mobile if they reach mobile states by the thermal excitation. It is essential to note that when thermal activation predominates, carriers do not necessarily hop to the mobility edge but strictly to the energy at which the vibration of the wave function decreases toward the Boltzmann factor.^350^ The ME model is applicable to materials that are polycrystalline, where the mobile states are extended band states of the crystallites and the trapped states are located in the disordered regions between the crystallites. Thus, hopping directly between trapped states is a competing transport mechanism.^355^ The multiple trapping model further describes hopping of photoexcited carriers in amorphous materials such as *a*-As_2_Se_3_^356^ and *a*-Si:H,^357^ as one which occurs directly between localized, band-tail states giving rise to a new path for energy deactivation at low temperatures. At times beyond the segregation time (time expended for the separation of mobile and localized states), e.g., 1 μs in *a*-As_2_Se_3_ and *a*-Si:H, the mobility is characterized by thermal excitation leading to multiple trapping. However, at lower times, charges move directly to lower energy states.^355^ Typically, the rate at which carriers hop away from deep or shallow states is smaller for deeper initial energies based on the exponential dependence of the rate on energy difference (effectively an activation energy) and on wave function overlap on energy. This invokes energy-induced state divisions which could be either fast or slow. Shallower states have fast detrapping rates, and carriers will depart as fast as they arrive and accumulate in deeper states where detrapping rates are slow and hence, carrier population is high. The energy which separates these fast and slow states is time- and temperature-dependent and is referred to as demarcation energy.^355^ The demarcation energy is the energy that defines the instance where the hopping-away rate is equivalent to the inverse of observation time. The mobility values obtained from the multiple trapping model of organic-based semiconductors respond quickly to small variations of trap characteristics when carrier concentrations are small. Again, the mobility shows temperature dependence (Arrhenius relation of mobility), and this has been demonstrated by both experimental and theoretical findings for the diketopyrrolopyrrole-naphthalene copolymer (PDPP-TNT)^358^ as displayed in Figure 36.

Polymer-based semiconductors are usually characterized by a π-conjugated backbone. Typically, charge transport in polymer-based semiconductors is affected by conformational disorder in the polymer backbone^356^ and chemical defects.^357^ The pronounced conformational disorder in polymers, especially those containing aromatic rings causes deviations from planarity in the polymer backbone, and this leads to a decrease in charge mobility along the polymer chains. The above phenomenon has been investigated by Choi et al.,^356^ using PANI- and PPy-doped with perchlorate as conducting polymers. They reported that distortions in the aromatic ring orientations of PPy and PANI are driven by temperature and film thickness, respectively. Within 220–240 K, the PPy doped with a perchlorate counterion exhibited structural changes which induced ring orientation and subsequent modification of charge mobility. Thicker films of PANI on gold displayed an out-of-plane tilt of the aromatic backbone with different conductance. Also, defects in molecules may act as trap states^358^ which largely affects charge mobility. The dependence of charge mobility on morphological features of polymer semiconductors have also been reported.^360^ Morphological studies performed on regioregular P3HT has revealed that low molecular weight (MW) films have a higher degree of crystallinity than high MW films. In contrast, high MW, less-ordered films favor high charge mobility because the films are longer than the domains, thereby minimizing the effects of grain boundaries by bridging neighboring grains. The charge transfer between polymer chains and the persistence length are dependent on the molecular packing.^361^ Hence, accounting for the physical nature of the interactions behind the molecular packing and their subsequent connection to the charge transport properties is essential for modeling and designing crystalline organic semiconductors.^362^ The molecular packing can be tuned by various intermolecular interactions including hydrogen bonding, halogen bonding, and π–π stacking.^344^ The influence of intermolecular interactions on molecular packing and the consequent effect on charge transport has been demonstrated and reported for perylene diimide dyes (PDIs).^362^ The intermolecular electrostatic O-π and O–H interactions in PDIs cumulatively impact the solid-state/molecular packing and the dimensionality and directionality of the charge percolation network. Charge percolation is a transport process in anisotropic polymer materials where there are different charge transport paths.^363^ Two-dimensional π-stacking motifs in PDIs is beneficial to high electron transport unlike one-dimensional π-stacking motifs which have obstructed electron transport due to dynamic disorders in intermolecular couplings and energies.

These different properties of polymer semiconductors have been studied using quantum mechanical and molecular simulation methods^364^ to gain insights into the optical and transport properties of π-conjugated polymers,^365^ thereby serving as a guide for the design of next generation systems. Theoretical simulations have aided in the computation of effective mass tensor for relevant bands which are useful in the estimation of the exciton binding energy associated with the lowest electronic LUMO–HOMO transition in π-conjugated polymeric semiconductors.^365^ The associated binding energy of an effective mass of 0.09*m*_e_ is about 0.2 eV and an exciton radius of about 20 Å. These details are integral to the understanding of the optoelectronic properties of the polymer. Recently, DFT calculations have been used to derive a transport model for polycrystalline polymer-based semiconductor by combining the contributions of the electronic structure of the crystalline domain with a model of interaction at grain boundaries (interface between two crystallites).^366^ The study focused on crystalline poly(3-alkylthiophene) (P3AT), an organic polymer with high charge mobility, to construct a reasonable density of states model combined with transport mechanisms for easy calculation and comparison of its conduction. The model adopted three assumptions viz., (1) transport is confined in a single plane of the lamella; hence, a two-dimensional density of states is used for the ordered lamella, (2) an exponential band tail of localized states represents the disordered regions between the ordered grains, and (3) transport in the ordered region/lamella is characterized by constant mobility. The main findings revealed that structural ordering in polymers has two significant effects. These include a higher mobility transport path accessible to holes due to the presence of lamella in the polymer structure. Second, the volume of amorphous material is decreased, causing a reduction in associated localized-state density. Hence, even if transport is dominated by hopping in the disordered material, the conductivity will increase because the Fermi energy moves further up the DOS for a given gate voltage, and the exponential increase in hopping conductivity increasingly offsets the reduced volume of material.

Other models such as atomistic molecular dynamic (MD) simulations and static lattice (SL) calculations have jointly been used to investigate the structure of poly(3-butyltiophene) (P3BT) as a function of temperature and pressure.^367^ In P3BT, the thiophene rings are connected at the two carbon atoms in the 1-position relative to the sulfur atom, with alkyl substituents sitting at one of the two 3-positions, and this conforms to the general lattice structure of P3ATs.^368^ It has been established through atomistic simulation that the butyl side chain in P3BT can assume cis and gauche conformations which are thermally accessible. This may induce torsional distortions that trigger the deformation of the π-conjugated polymer backbone by energy transmission from thermally induced sources stored in the alkyl side chain and can be observed as a dilation of the forbidden gap.^368^ Similarly, atomistic MD and SL simulations conducted on P3AT have revealed that at ca. 6–7 GPa, planarization of the polymer main chains is initiated. This process is usually accompanied by a spontaneous tilting in the alkyl side chain. However, it is archetypal of the polymer to exhibit a reduction in this “tilting” at pressures beyond 10 GPa.^369^ Thus, it is evidential that temperature and pressure largely affect the structure of π-conjugated systems, and therefore, the scientific community can capitalize on this to compound the existing database on the structure activity relationship (SAR) of organic polymers. The static considerations adopted in modeling are gradually being replaced and/or complemented by dynamic considerations where the time-dependent nuclear motions inform the overall charge dynamics of the material.^370^ For instance, the intramolecular transport of charge carriers in poly(phenylenevinylene) (PVP) and P3HT was calculated by tight-binding approximation combined with static disorder along the chain.^371^ The polymers were modeled by a chain of sites that are consistent with the monomer repeat-units, and therefore, charge migration on this chain is described by the Hamiltonian.^370^ Next, the results revealed that the distinct effects of static and dynamic disorder on particle motion trigger two stages of particle propagation. In that, at short times the particles move ballistically (i.e., exhibits maximum velocity over a short period of time), while at lengthy times their propagation is diffusive with higher dispersions for higher degree of static disorder. Basically, static disorder creates a friction for the particle propagation, whereas the dynamic disorder may drive or impede the intersite transfer depending on the degree of static disorder.^371^

Despite the inability of the current developed modeling methods to quantitatively predict the charge transport characteristics of the polymeric semiconductor materials used in making the photoelectrodes, the major progress that has been made in this field so far cannot be overlooked. This has paved the way for researchers and experimentalists in the community to study an array of plausible materials and their properties prior to their use as the main semiconductor for the design of photoelectrodes. Increasing the diversity of the polymers investigated experimentally and computationally is needed to help generate a more holistic understanding of the relationship between physical, chemical, and electronic properties of conjugated polymers.

## 7 Conclusion and Outlook

Solar fuel synthesis stands to make a significant impact on a sustainable future. Especially, the PEC approach for producing H_2_ from water and high value-added chemicals such as methanol and ethanol by utilization of CO_2_ waste has the potential to develop a zero carbon-emission economy. The limitations of inorganic materials for engineering photoanodes and photocathodes were discussed in the review, and the advantages such as low cost, earth abundance, ease of band position tuning, and sustainability of the polymer photoelectrodes were detailed. As the polymer-based materials have been extensively studied for particle suspension-based photocatalysis, our focus in this review is to bring attention to emerging polymer-based photoelectrodes for PEC reactions, which generates reduction and oxidation products separately, thus avoiding the back reaction and mitigating the risk of explosion *in situ*.

The emerging polymers including CN_*x*_, COF, MOF, P3HT, etc. have shown noticeable photocurrent density for either the reduction or oxidation reaction under one sun irradiation. A few strategies have been reviewed to improve the photocurrent density, i.e., the intimate contact between the polymer and the substrate, appropriate defects engineering, microstructure, doping, and junction structure. Furthermore, the key role of the molecular/nanoparticle cocatalyst has been highlighted in conjunction with the preparation methods, including both activity improvement and stability enhancement. Among these, engineering BHJ has been considered as one of the key strategies to enhance the photocurrent density.^147^

Engineering the polymer BHJ, especially using P3HT:PCBM (donor:acceptor) and sandwiching it between electron and hole acceptor layers, has been considered an efficient way to increase the photocurrent density of the photocathode for the reduction half-reaction. For instance, a benchmark photocathodic current density of 8 mA/cm^2^ at 0 V vs RHE was achieved using the P3HT:PCBM BHJ architecture positioned between the CuI hole selective layer and the Pt-decorated TiO_2_ electron acceptor layer.^148^ A similar approach was used to engineer the photoanode as well using benzodithiophene-based polymer PBDTTTPD as the electron donor and naphthalenediimide-based polymer PNDITCVT as the electron acceptor, which achieved a high water oxidation photocurrent density of 2 mA/cm^2^ at 1.23 V vs RHE, in which mesoporous ZnO was used as the electron transport layer and Co_3_O_4_ as the water oxidation cocatalyst.^118^ Furthermore, a BHJ between p-type polymer PBDB-T and n-type polymer ITIC showed the record oxidation photocurrent density of 15 mA/cm^2^ at 1.23 V vs RHE, in the presence of oxidation cocatalyst NiFe-LDHs.^116^

It must be noted that with the exception of the above system, the general performance of BHJ photoanodes lags behind that of BHJ photocathodes.^317^ This is owed to the fact that polymers are usually n-type semiconductors, thus having favorable energetic alignment to match the electrochemical potential needed for efficient HER other than OER. Also, the high kinetic overpotential involved in OER^372^ further disfavors polymer-based semiconductors to meet the electrochemical potential requirement for efficient OER. The HOMO level position of polymer-based semiconductors is not positive enough to generate the needed thermodynamic driving force for water oxidation compared with the best performing metal oxide inorganic semiconductors. Typically, the composition of the VB maximum of metal oxide semiconductors is dominated by O 2p orbitals.^81^ The orbitals that dominate the HOMO of polymer-based semiconductors, analogous to the VB of metal oxide semiconductors, usually vary depending on the structure of the polymer. For instance, based on density functional theory (DFT) calculation, it has been established that the HOMO of heptazine which is the building block of CN_*x*_ is localized on those N atoms in the heptazine ring that are directly bonded to two carbon atoms (N_2C_).^373^ This suggests that the HOMO of CN_*x*_ comprises N 2p orbitals which are positioned at a more negative electronic potential than the O 2p orbitals.^374^ Thus, the O 2p orbitals in metal oxide semiconductors are positioned at a more positive electronic potential which readily favors water oxidation, as opposed to polymer-based semiconductors. Nonetheless, lateral bay extension of rylene diimides can lower the HOMO (shift to more positive potentials)^375^ sufficiently to thermodynamically allow water oxidation, as exemplified by the recently reported chlorinated rylene diimide-based acceptors.^376^ These acceptors were used in BHJ photoanodes with thiophenedicarboximide-benzodithiophene (TPD-BDT) donors and notably sustained PEC performance over 3 h under sacrificial electron donor conditions. Although all these aforementioned benchmarks demonstrate the significance of junctions and synergy between junctions and cocatalysts for PEC fuel synthesis, the challenge for the optimization of the general performance of polymer-based photoelectrodes, especially photoanodes, cannot be overemphasized.

Although many organic or polymer semiconductors have been widely used in organic solar cells and light-emitting diodes, few were used in PEC for solar fuel production. One major reason is the poor contact between the polymer semiconductors and the aqueous electrolyte^377^ at the interface or bad hydrophilicity of the polymer surface, which makes the reactant adsorption and charge injection into the reactant molecule challenging. The deposition of a layer of hydrophilic functional group would help to sort out this issue; however, it might screen the light absorption. In some cases, organic molecular dyes have been used as light-harvesting materials, which is further coated with hole and electron separation layers of inorganic metal oxides. In the view of energy levels, only limited polymer semiconductors have either suitable LUMO levels to drive the reduction reaction at the water interface or appropriate HOMO levels to produce high photocurrent.^378^ The next major reason is the short lifetime of the excitons in the polymer semiconductors, leading to a poor charge carrier separation and diffusion in the polymers.^379^ To increase the degree of polymer crystallinity improves the charge carrier separation, such as by controlled annealing in different atmosphere.^71^ Moreover, there are many steps involved during the PEC process, such as adsorption of water and protons, charge injection, bond breaking, intermediate formation, and desorption of the products, hence the mechanistic investigation is crucial but these are hardly investigated for organic polymer semiconductors as we discussed in Section 5. Another important limiting factor is the poor stability of the polymer semiconductors in aqueous solution under the strong light irradiation condition.

Polymer-based photoelectrodes have advantages in terms of cost, earth-abundance, ease of synthesis, etc. However, the PEC performance of polymer-based photoelectrodes, except the P3HT and PBDB-T-based ones listed above, is mainly 10 times smaller than their inorganic counterparts. There are many reasons demonstrated for not being competitive to the inorganic semiconductor-based photoelectrodes at present, including poor hydrophilicity and stability, moderate efficiency, a limited number of polymer semiconductors, and poor understanding of charge separation.^58,380^ However, demonstrations such as those by Comas Rojas et al.^148^ and Bourgeteau et al.^145^ have established that the photocurrent obtained by BHJ polymer-based photocathodes can be potentially comparable to that of their inorganic counterparts. Therefore, with the urgent need for an efficient and low-cost photoelectrode and taking into account the very recent fast development of polymer photoelectrodes, herein we would like to highlight several strategies to improve the PEC performance of the polymer-based photoelectrodes for solar fuel synthesis and elaborate on challenges remaining in the field that impede progress.

First tuning the bandgap by, for example, appropriate doping, modifying the polymer structure, and forming a junction is important to enhance light harvesting. Then the polymer photoelectrode and electrolyte interface has to be optimized by experimental and theoretical approaches, as it is crucial to determine the charge injection to the adsorbate for transforming small molecules into useful fuels and high-value chemicals. The thickness of the polymer film plays a crucial role as the charges in most of the polymers have a short diffusion length (between nanometer and micrometer). While a thick film can maximize light absorption, the desired thickness should balance the light absorption and charge diffusion, which can be achieved by optimizing the concentration/amount of precursors, and polymerization time during synthesis.

Next, the resistance of charge transfer has to be reduced. To achieve this, the contact between a substrate and the film must be improved, such as by depositing the film using appropriate pretreatment and deposition techniques. The PEC performance of the photoelectrodes also depends on the nature of the film (e.g., dense and degree of crystallinity) and the structure of the polymer, the film thus should be optimized by the film deposition conditions. With respect to the surface reaction, engineering the different facets to gain more active sites and better charge separation are also essential. To increase the contact area of the polymer–electrolyte interface can improve the adsorption of small molecules and enhance the surface reaction, which can be achieved by preparing the nanoarchitecture of the polymer film and/or porous surface morphology. More importantly, the stability of the polymer-based photoelectrode can be prolonged by using a transparent low-cost protective coating while it should not inhibit the photoabsorption of the polymer. The suitable electrocatalyst or cocatalyst thin layer can also provide better protection to the photoactive polymer film besides catalyzing the surface reaction.

This last point on stability can be seen as the “elephant in the room” for polymer-based photoelectrodes. While efforts have been made in the design of systems with robust interfacial layers and protective strategies to improve photocurrent stability, current systems are still short of necessary operational times. There is a clear need to have photocurrent stability beyond hundreds of minutes to make systems viable.^381^ Presently, lifetime testing of PEC cells beyond 48 h have rarely been reported.^382^ Generally, the instability of polymer-based PEC systems arises from poor stability of polymer-based semiconductor materials when in contact with an aqueous electrolyte. The inherent partial permeability of organic polymers to water^383^ exposes them to dissolution and subsequent delamination during practical application. Haro et al.^146^ revealed that the common organic hole transport layer PEDOT:PSS in a polymer photocathode dissolved in the presence of an aqueous electrolyte, causing delamination of the organic active layer. Notably, all OER systems highlighted in this report exhibit deactivation by manifesting gradual photocurrent decay over relatively shorter time scales unlike their inorganic counterparts.^384^ Of note, most of these systems were those without stability control mechanisms in the form of an additional protective layer made up of inorganic materials.^58^ For instance, the best photoanode reported herein is ITO/PBDBT/ITIC/GaIn@Ni/NiFe-LDHs, which displayed a photocurrent of 15.1 mA/cm^2^ at 1.23 V vs RHE. A stability test performed at 1.3 V vs RHE for 10 h revealed that 90% of the initial photocurrent density are retained on the passivated photoactive layer, whereas the same photoactive layer without passivation showed poor activity within a few minutes.^116^ This result highlights the benefits of a passivation layer, which appears to be necessary for protection of at least some of the polymer materials used.

It is without doubt that data on the lifetime of an electrode would support reliable technoeconomic analyses for PEC systems in the context of realistic lifetime projections. The PEC cell life span in addition to the solar-to-hydrogen (STH) efficiency has been shown to be an integral parameter in the life cycle energy balance of a PEC system.^385^ It is therefore necessary to develop standard lifetime testing of PEC cells to serve as a reference point in assessing the different organic and inorganic based prototypes that are being developed.^50^ While the utilization of protective coatings can be effective, it is essential to acknowledge that the current materials which are corrosion-sensitive may make large-scale production challenging since any irregularity in the protective layer would amount to increased production cost as a result of elevated failure rates and, hence, a compromise on the economic viability of the production technique.^58^ As has been established in Section 2 of this report, the fabrication process of the thin films dictates their microstructural properties which in turn influence their performance. It is required that the thin films exhibit an intimate contact with the conducting substrate for efficient charge transfer and better photoelectrode performance. If the contact between the photoelectrode and the conducting substrate is not strong, there is the likelihood of catalyst detachment during practical application, which is detrimental to photoelectrode response.^86^ Several strategies including a solvothermal method have been used to obtain intimate contact between the photoelectrode and the conducting substrate. The less durable physisorption approach^386^ of immobilizing molecular catalysts onto the photoabsorber has now been replaced with a chemical immobilization strategy which proceeds via strong anchor groups such as −COOH^387^ and PO_3_H_2_^388^ to ensure that the catalyst is covalently bound to the electrode surface.^389^ However, there are still reports of photocurrent instability assigned to catalyst detachment.^390,391^ It is unclear whether this is occasioned by interfacial behavior during practical application since information on photocurrent degradation in relation to the structure of the photoelectrode/catalyst is elusive in the field. Without knowledge of the photocurrent degradation mechanism, targeted improvements cannot be made. For example, it has been determined that the photocurrent degradation exhibited by solution-processed organohalide perovskite solar cells during practical application is an effect of the formation of light-activated metastable deep-level trap states.^392^ This limitation can immediately be addressed by operating the device at a lower temperature (0 °C) since photocurrent degradation normally exhibits a steep temperature-dependence.^392^ 2D perovskites structures have also been used to greatly enhance the stability of typical 3D perovskites^393^ by inhibiting moisture-induced degradation at grain boundaries^394^ and improving crystallinity to reduce the number of active defects that can lead to degradation.^395^ Similar insights into the mechanism of photocurrent degradation of polymer photoelectrodes are needed to move the field closer to viable devices.

The application of polymer photoelectrodes as photoabsorbers for H_2_ generation by splitting water can be as efficient as the existing fossil fuel-based technologies for large-scale H_2_ production.^396^ However, the development of polymer-based photoelectrodes for large-scale H_2_ production and subsequent commercialization is still at the early stage. The most important factor to an immediate commercialization of this technology is the knowledge of the overall STH efficiency since the higher the STH efficiency, the better the energy return commensurable to their commercialization. Other important factors including the energy used to fabricate the PEC cell, the lifespan of the PEC cell relative to the energy payback time, and the cost of the energy output must be assessed. Today, the base-case conditions for full commercialization of PEC technology for H_2_ production include a 10-year lifetime for photoelectrodes with 10% STH and within a levelized cost range of 2–4 USD per kg of H_2_ as mentioned before.^54^ It must be noted that the new cost targets by 2031 are lower than the previous ones as brought forward by the US Department of Energy Hydrogen Shot, part of their Energy Earthshots program. For an inorganic semiconductor-based PEC cell with STH efficiency of 20%, it is estimated that the time span within which the cell can produce energy equivalent to its manufacturing energy (energy payback time) is 3 years.^397^ Even though polymer-based photoelectrodes can be carefully optimized to increase STH efficiency, it is unclear if the current optimization strategies may incur further cost and/or compromise the lifespan of the resulting PEC cells. Also, the commercial deployment of polymer-based photoelectrodes can only be an option in the foreseeable future if they can be appropriately tuned to deliver a stable performance over the extended lifetime of the resulting PEC device.^50^ The techno-economic analysis published very recently^398^ concluded that based on the levelized cost of hydrogen (LCOH_2_), PEC systems were not cost-competitive with existing alternatives on the market. However, it is important to note that the PEC system considered utilized an inorganic semiconductor photoactive layer (c-Si) which is usually cost-intensive. The PEC module cost for a Si-based system with 10% STH efficiency was estimated at 154 USD/m^2^. While the fabrication process cost was not detailed, the price of the c-Si light absorber was 31% (48 USD/m^2^) of the module cost.^398^ Another techno-economic analysis based on inorganic PEC to produce H_2_ determined that the PEC cell was ∼80% of the total project cost.^399^ The LCOH_2_ was at best ∼2 USD/kg, but the minimum cost of the PEC cell was 700 USD/m^2^. We are not aware of published techno-economic analyses of polymer-based PECs at this time but we can look at those of OPV to gain further insights. The cost of mass-produced OPV modules has been estimated to be in the vicinity of 10 USD/m^2^,^400−402^ much lower than the costs considered for the inorganic PECs. While a direct comparison cannot be made due to the different cell/module design (e.g., the additional need for membranes and cocatalysts for the PEC), it seems reasonable to expect that optimized organic PEC cells could cost <100 USD/m^2^ and lower the LCOH_2_ to the target of 1 USD/kg. More efforts should be channeled into the techno-economic analysis of PEC H_2_ production systems that utilizes polymer-based photoelectrode active layers and cost-effective cocatalysts.

Despite the appreciable amount of work over the past decades regarding the application of organic-based photoelectrodes for solar-driven reactions, only a few reports have presented comprehensive fundamental understanding, combining spectroscopic and electrochemical data that provide useful information on the behavior of the charge carriers generated in the photoelectrode and at the interface of photoelectrode and electrolyte. This is reflected in the rather limited number of papers we could identify that investigated the charge carrier dynamics in organic photoelectrodes. Section 5 is the comprehensive review of those we found. Notably, we did not identify any papers that describe the charge carrier dynamics of CO_2_ reduction by polymer PEC devices.

Regardless of the different mechanistic pathways assigned to the reduction reactions in organic and inorganic systems, it is incontrovertible that these reactions are highly selective to specific chemical sites/species within the system.^403^ For example, for metal-oxide inorganic photoelectrodes, HER proceeds via a homolytic or heterolytic path involving a metal hydride intermediate. In contrast, the HER mechanism on organic-based photoelectrodes tends not to be as straightforward as metal-oxide photoelectrodes, which have a similar general surface structure. The possible active sites of the organic material are broad, varying upon polymer structures and presenting some difficulty in assigning a general mechanism to HER. The *operando* EC-Raman spectroscopic studies with carbon-based conjugated polymer show that the active sites/species involved in HER may not necessarily be metal-centered catalytic sites but rather excited state antibonding orbitals with localized electrons.^308^ The previous studies^404,405^ on metal-centered inorganic electrocatalysts revealed that the physical location of reactive species could extend beyond the atomic surface of the metal center to some degree into the bulk material. This suggests that the number of reactive species and their intrinsic activity determines the overall catalytic activity of the electrocatalyst. We think that this concept may hold for organic systems, in particular for porous polymer photoelectrodes, albeit it is yet to be demonstrated and reported.

In line with an outlook on the development of cutting-edge techniques toward the engineering of highly efficient next-generation organic-based or polymer PEC devices, we propose 5 avenues to focus on. (1) There is a need for remarkable spectroscopic and electrochemical-based *operando* studies to fully describe the behavior of photogenerated charge carriers and their corresponding impact in a real device. This will deepen our understanding of the function of each component and provide effective strategies for photoelectrodes design. (2) Extend the study of charge carrier dynamics of organic-based PEC devices to other photoreactions, e.g., CO_2_ reduction or N_2_ reduction to fully understand the role of interfacial charge transfer and to take advantage of the tunability of organic materials. (3) Advancement of studies into charge carrier dynamics of PEC systems built from π-conjugated organic or polymer materials other than CN_*x*_ and thiophene. As has been demonstrated with continued efforts in the understanding of inorganic photoelectrodes and organic PV systems, studying a breadth of materials will allow us to develop general design guidelines that link the effects of material design strategies with the performance of organic-based photoelectrodes. (4) Modeling is a powerful tool to screen photoelectrode and cocatalyst candidates, which not only save researchers time but also avoid wasting raw materials. (5) A promising technique toward the acceleration of polymer materials discovery is high-throughput experiments combined with artificial intelligence (AI).^406−410^ The increased rate at which materials can be tested and discovered holds tangible prospects in revolutionizing scientific discovery, with key impacts already being made in material science.^411^ The fast development of the discovery of polymer photocatalysts provides a large database. Utilizing such a large database, AI^412^ can also predict the stability issues such as due to the Ostwald ripening, particle migration, and coalescence of the photocatalyst or cocatalyst nanoparticles during the reaction, which will save the time for trial-and-error experimentation and enable lab-to-fab translation.

Finally, an important note for this society is that apart from the measurement of photocurrent density of polymer-based photoelectrodes, faradaic efficiency, IPCE, and STH should be reported in parallel in order to rationalize and fairly compare the performance of different photoelectrodes. On the basis of these, we believe that the performance of organic photocathodes is very promising and would gradually advance to levels, which are superior to, or at least comparable to their inorganic counterparts while by a very low-cost process.
